# Control of Oriented Tissue Growth through Repression of Organ Boundary Genes Promotes Stem Morphogenesis

**DOI:** 10.1016/j.devcel.2016.08.013

**Published:** 2016-10-24

**Authors:** Stefano Bencivenga, Antonio Serrano-Mislata, Max Bush, Samantha Fox, Robert Sablowski

**Affiliations:** 1 Cell and Developmental Biology Department, John Innes Centre, Norwich Research Park, Norwich NR4 7UH, UK

## Abstract

The origin of the stem is a major but poorly understood aspect of plant development, partly because the stem initiates in a relatively inaccessible region of the shoot apical meristem called the rib zone (RZ). We developed quantitative 3D image analysis and clonal analysis tools, which revealed that the *Arabidopsis* homeodomain protein REPLUMLESS (RPL) establishes distinct patterns of oriented cell division and growth in the central and peripheral regions of the RZ. A genome-wide screen for target genes connected RPL directly to many of the key shoot development pathways, including the development of organ boundaries; accordingly, mutation of the organ boundary gene *LIGHT-SENSITIVE HYPOCOTYL 4* restored RZ function and stem growth in the *rpl* mutant. Our work opens the way to study a developmental process of importance to crop improvement and highlights how apparently simple changes in 3D organ growth can reflect more complex internal changes in oriented cell activities.

## Highlights

• Image and sector analysis revealed 3D growth patterns in early stem development
• *Arabidopsis RPL* controls oriented cell division and growth in the rib meristem
• RPL interacts with many of the key genes that regulate shoot organogenesis
• *RPL* controls oriented growth by directly repressing organ boundary genes

## Graphical Abstract

**Figure:**
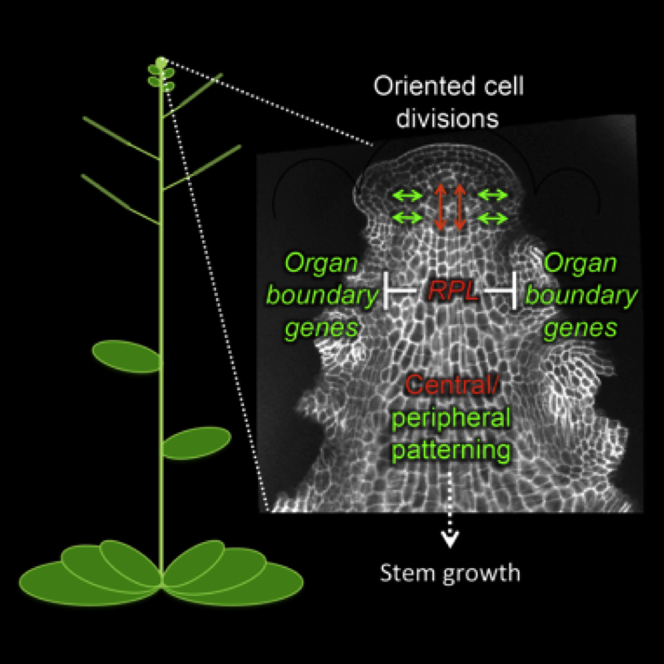

## Teaser

The origin of the stem is a major gap in our understanding of plant development. Bencivenga et al. show that during stem initiation in *Arabidopsis*, the regulatory gene *REPLUMLESS* controls three-dimensional patterns of cell division and growth through repression of organ boundary genes.

## Introduction

Virtually all plant growth is sustained by stem cell populations located within the apical meristems (Aichinger et al., 2012). Decades of intense study have revealed much about how the meristems form roots, leaves, and floral buds. In contrast, little is known about how the stem is initiated in the subapical region of the shoot meristem and how regulatory genes that function in this region influence stem size and shape. The origin of the stem is not only a major aspect of plant development that has been relatively neglected, but is also of great importance in crop improvement: genes that modify stem development have played a key role in yield increases in the last 50 years (Khush, 2001), but the developmental basis for their effects on plant architecture remains unclear.

**Figure 1:**
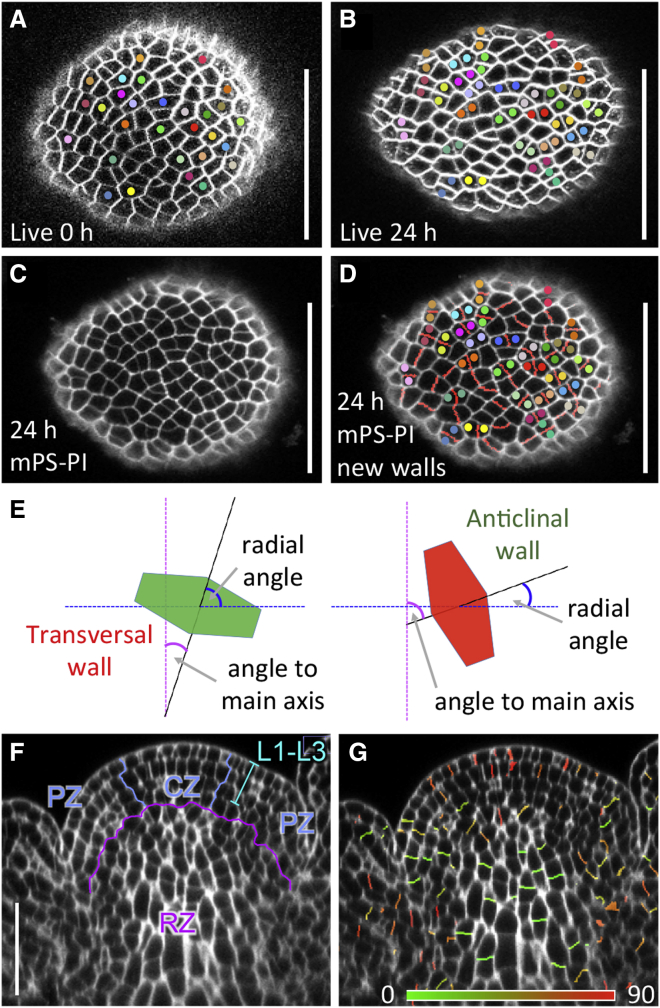
Automated Detection of the 3D Orientation of Cell Divisions in the Shoot Apex (A and B) Confocal sections through the outer layers of a live *Arabidopsis* (L-*er*) inflorescence meristem stained with FM4-64 at 0 hr (A) and 24 hr later (B). When cell divisions occurred, mother cells in (A) and their corresponding daughter cells in (B) were manually marked with dots of the same color. (C) Confocal section of the same meristem as in (B), after staining by modified pseudo-Schiff propidium iodide (mPS-PI), showing variable intensity of cell-wall signals. (D) Same section as in (C), overlaid with an image of segmented cell facets detected as new walls (red lines); colored dots mark recent divisions corresponding to (B). Note that new walls were correctly attributed for most of the recent divisions (27 out of 29), in addition to low-intensity walls that likely correspond to divisions completed more than 24 hr earlier. (E) Scheme of how the orientation of new walls was measured. Walls are represented by colored hexagons, with a line normal to their best-fitting plane shown in black; the magenta line is parallel to the main axis of the stem; the blue line is perpendicular to the to the main axis of the stem and passes through the wall's center of mass; angles to the main axis and radial angles are indicated by the magenta and blue arcs, respectively; the red and green walls correspond to transversal and anticlinal walls, as frequently seen in the RZ and in the outer meristem layers, respectively; anticlinal walls can have small to large radial angles, depending on whether they face the central axis or not. (F and G) Longitudinal slice through a stack of confocal images of mPS-PI-stained inflorescence apex, with (F) the outer meristem layers (L1–L3), central zone (CZ), peripheral zone (PZ), and rib zone (RZ) indicated. (G) Image corresponding to (F), overlaid with an image of cell facets detected as new walls and colored according to the angle to the main axis of the stem. Scale bars, 50 μm. See also Figure S1.

The shoot apical meristem, which produces leaves, flowers, and the stem, has distinct zones with different functions (Fletcher, 2002) (Figure 1F). Leaves and floral buds are initiated in the peripheral zone (PZ), while long-term progenitors in the central zone (CZ) constantly replenish the PZ. The underlying rib zone (RZ) gives rise to the stem and includes a central region called the rib meristem (named after its distinct pattern of transversal cell divisions), which gives rise to the pith, and a peripheral region that appears continuous with the overlying PZ and gives rise to the epidermis, cortex, and vascular tissues of the stem (Sachs, 1965, Sanchez et al., 2012). Superimposed on this functional zonation, the shoot meristem has a layered structure; in angiosperms such as *Arabidopsis*, the cells in the outermost two to three layers divide mostly anticlinally (perpendicular to the meristem surface), so their descendants generally remain in the same layer (Fletcher, 2002). Growth of the different meristem regions can be controlled differentially: during the vegetative stage in *Arabidopsis*, the CZ and PZ sustain leaf production but the RZ is inhibited, whereas at the transition to flowering, activation of the RZ leads to rapid stem elongation while the CZ and PZ start to produce floral buds.

Parallels can be drawn between activation of stem growth at the shoot apex and the well-studied control of root growth at the opposite end of the plant's main axis (Aichinger et al., 2012), but there are important differences. In the root, terminal growth mostly precedes the emergence of lateral roots and the vast majority of cell growth and division is aligned with the main root axis, so growth rate is proportional to root meristem size and to the overall rate of cell proliferation (Beemster and Baskin, 1998). In contrast, development of the stem occurs simultaneously with that of lateral structures such as flower buds, and cell files in the RZ appear much less organized than in the root. The more complex structure of the RZ requires three-dimensional (3D) analysis of cell behavior and overall organ growth. A further complication is the relative inaccessibility of the RZ in comparison with the root meristem. Thus RZ growth and early stem development remain considerably less well understood than the root system, and illustrate the general challenge of describing and understanding the regulation of tissue growth in 3D structures with no obvious internal landmarks.

In *Arabidopsis*, one of the master regulators of stem growth is most often named *PENNYWISE* (*PNY*) (Smith and Hake, 2003), *REPLUMLESS* (*RPL*) (Roeder et al., 2003), and *BELLRINGER* (*BLR*) (Byrne et al., 2003); we used *rpl* mutant alleles and therefore adopted *RPL* for simplicity. *RPL* encodes a BEL1-like TALE homeodomain (BLH) transcription factor that controls multiple aspects of meristem and floral development, including meristem maintenance, the distribution of lateral organs around the meristem (phyllotaxis), the transition to flowering and the associated activation of stem development, and subsequently floral organ patterning (Byrne et al., 2003, Roeder et al., 2003, Smith and Hake, 2003, Arnaud et al., 2011). Based on its expression in the shoot meristem, extending into the RZ (Smith and Hake, 2003, Andrés et al., 2015), *RPL* likely affects stem growth by regulating the earliest steps in stem development, but the molecular and cellular processes controlled by *RPL* in the RZ are virtually unknown.

Here, we used quantitative 3D imaging and clonal analysis to reveal how *RPL* controls early stem development. Our findings indicate that *RPL* controls RZ function through oriented cell activities rather than local rates of cell proliferation. We also show that RPL directly interacts with many of the key regulatory genes in shoot organogenesis and that interaction with genes involved in organ boundary development are particularly important for the role of *RPL* in the RZ.

## Results

### RPL Is Required for Oriented Tissue Growth in the RZ

If *RPL* controls morphogenesis in the RZ, it would be expected to modify rates or orientations of tissue growth, or a combination of both. To verify this we would require new imaging and analysis methods, because tracking cells by live imaging (Serrano-Mislata et al., 2015) is not feasible in the deeper layers of the shoot meristem, whereas high-resolution 3D images of fixed apices (Schiessl et al., 2012) cannot provide temporal information. Instead, we exploited the fact that new cell walls are placed perpendicular to the mitotic spindle (Smith, 2001), thus retaining information about the orientation of recent cell divisions. To detect recent cell divisions, we cross-linked wall polysaccharides to propidium iodide (PI) (Truernit et al., 2008), which would be expected to produce lower fluorescence for thinner, more recently synthesized walls. After 3D segmentation the PI signal was measured in all facets between cells, and individual facets were identified as new walls if they had the weakest signal density for both adjacent cells (details in Supplemental Experimental Procedures and annotated source code in Data S1). This method accurately detected cell divisions identified by time-lapse imaging in both outer and inner meristem layers, and correctly detected the predominance of anticlinal divisions in the outer meristem layers and of transversal divisions in the RZ (Figures 1 and S1). Thus information about 3D patterns of oriented cell divisions can be extracted from single-time-point images of fixed shoot apices.

**Figure 2:**
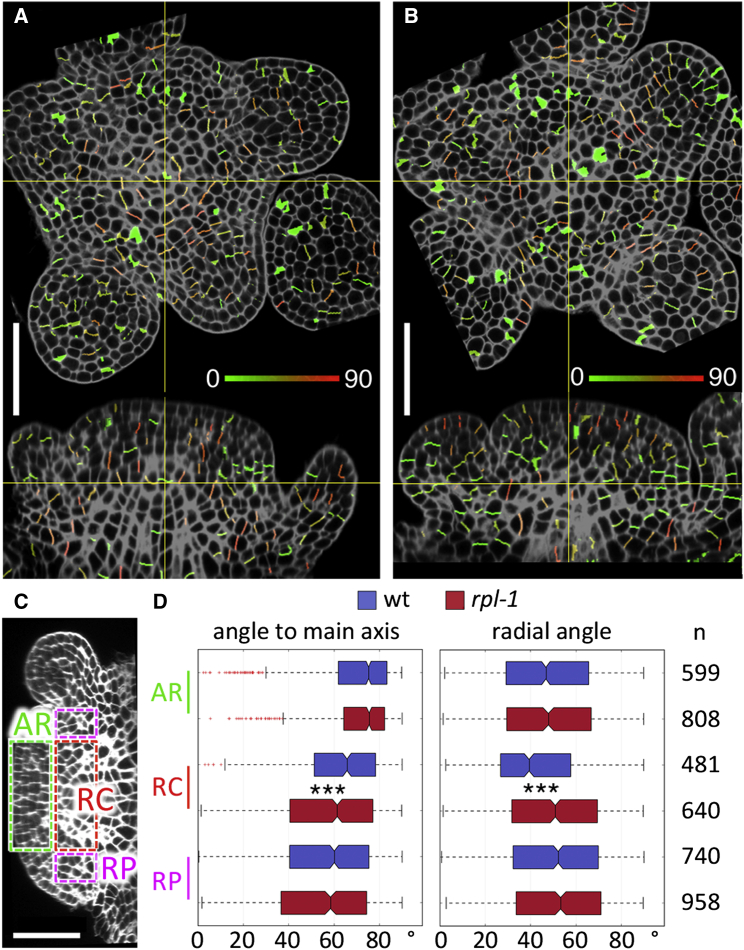
*rpl-1* Disrupts the Orientation of Cell Divisions in the RZ (A and B) Orthogonal views of confocal image stacks of wild-type (wt) (L-*er*) (A) and *rpl-1* (B) inflorescence apices stained by mPS-PI, overlaid with images of segmented cell facets detected as new walls, and colored according to their radial orientation (see Figure 1E). In each image, the yellow cross-hairs mark the same point in the top and side views; note the less organized RZ in *rpl-1* and the high frequency of radially oriented divisions in the periphery of the wild-type RZ, but not in the mutant. (C and D) Measurement of oriented divisions in wild-type and *rpl-1*. (C) Regions where new wall angles were measured (AR, apical region; RC, rib meristem core; RP, rib meristem periphery). (D) Boxplots show the distribution of new wall angles to the main stem axis or radial angles (see Figure 1E). n indicates the number of new walls in each set (combined data from four apices for each genotype); asterisks indicate statistically significant differences (^∗∗∗^p < 0.001, Mann-Whitney test). In the boxplots, the box extends from the lower to upper quartile values with a line at the median; whiskers extend to 1.5 times the interquartile range, and outlier points beyond the whiskers are shown in red. Scale bars, 50 μm. See also Figure S2.

We next used the method to compare shoot apices of wild-type and *rpl-1* mutant plants. The RZ of the wild-type apex showed a well-defined rib meristem with cell divisions perpendicular to the main stem axis, while the peripheral region was enriched for radial cell divisions, which potentially increase RZ width and may contribute to elongating the basal region of floral pedicels (Figure 2A). In contrast, *rpl-1* appeared to have a less organized RZ and the difference between the central and peripheral regions was less obvious (Figure 2B). To quantify the differences between wild-type and *rpl-1*, we compared the orientation of recent cell walls in the region where the rib meristem originates (“RM core” [RC], within 30–60 μm of the meristem summit and 0–40 μm of the main axis), in the overlying CZ and PZ cells (“apical region” [AR], within 30 μm of the meristem summit and within 40 μm of the shoot main axis), and in the PZ surrounding the RM (“RM periphery” [RP], within 30–60 μm of the summit and 40–50 μm of the main axis) (Figures 2C and 2D). Significant differences were detected in the RC, where *rpl-1* showed a pattern more similar to that in the RP, with more variable angles to the main axis and more radially oriented divisions; these differences were seen not only in data from combined apices but also across individual apices (Figure S2).

**Figure 3:**
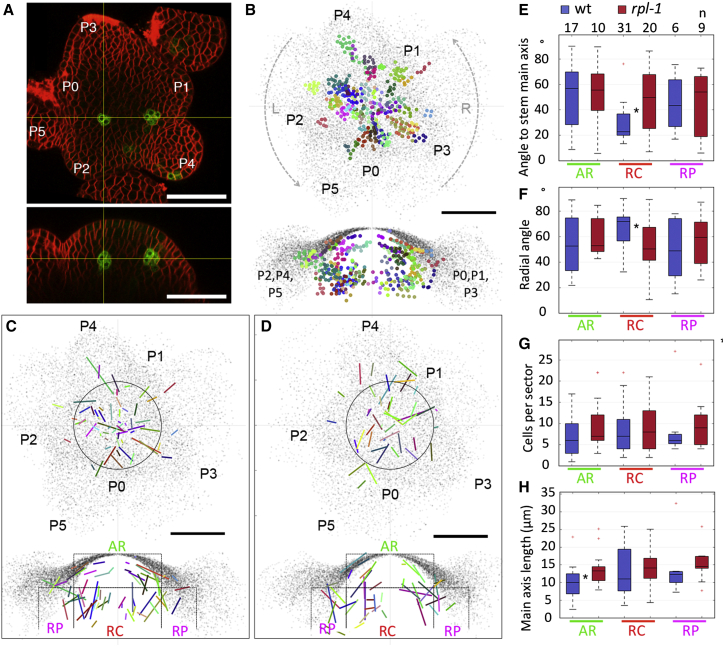
Clonal Analysis Shows that RPL Controls Orientation, but Not Rates of Growth, in the Central Region of the RZ (A) Orthogonal views of a confocal images stack of a wild-type inflorescence apex with two mGFP5-ER-marked clones (green), 3 days after Cre-catalyzed recombination. P1 to P5 mark the positions of successive floral bud primordia. (B) Vertical (top) and radial (bottom) projections of superimposed GFP-marked clones from 15 wild-type apices. Each clone is marked in a different color, with the position of individual cells indicated by dots. Each clone was projected onto a plane containing its center of mass and the stem main axis to produce the radial projection. Clones on the right and left sides of the vertical projection (arrows marked R and L) are shown, respectively, on the right and left sides of the radial projection. (C) Vertical (top) and radial (bottom) projections of the main axes of clones shown in (B). On the radial projection, the AR, RC, and RP regions are defined as in Figure 2C. (D) Projections of the main axes of clones from 15 superimposed *rpl-1* apices, produced as described for the wild-type clones in (B) and (C). (E–H) Boxplots showing the vertical angles of the main axes of clones (relative to the stem main axis) (E), radial angles (relative the shortest line between the clone's center of mass and the stem main axis) (F), number of cells per clone (G), and length of the clone main axes (H). AR, RC, and RP correspond to the regions shown in (C) and (D); n indicates the number of sectors in each region; asterisks indicate statistically significant differences (^∗^p < 0.01, Mann-Whitney test). In the boxplots, the box extends from the lower to upper quartile values with a line at the median; whiskers extend to 1.5 times the interquartile range, and outlier points beyond the whiskers are shown in red. Scale bars, 50 μm.

The orientation of cell divisions can respond to cell geometry, which reflects principal directions of growth, or to the direction of mechanical stress, which can accumulate during growth of interconnected cells, and these physical signals can also be overruled by chemical signals (Kwiatkowska, 2004, Besson and Dumais, 2011, Yoshida et al., 2014). To test whether oriented divisions corresponded to directions of tissue growth and to obtain information about growth rates, we used a Cre-*lox*P recombination system (Gallois et al., 2004) to mark individual cells with GFP expression and track their descendants in the shoot apex (Figure 3A). To overlap marked sectors from multiple apices and analyze them in 3D, we landmarked cells within each sector manually and used custom scripts to align the images and measure the position, size, and orientation of the main axis for each sector (details in Supplemental Experimental Procedures and annotated source code in Data S1). As expected from the anticlinal cell divisions in the outer layers of the meristem, sectors in these layers were oriented tangentially to the meristem surface (Figures 3B and 3C). The wild-type sectors also confirmed the expectation that the RP originates from the overlying PZ of the meristem, where lateral organs are also initiated. Sectors in the RC grew vertically and more slowly than in the surrounding region, and based on their orientation appeared to originate from a region below the CZ progenitors that sustain the initiation of lateral organs (Aichinger et al., 2012) (Figure 3C). Based on cell number and length of sectors, growth rates were not significantly different between wild-type and *rpl-1*; in contrast, the orientation of *rpl-1* sectors was different from the wild-type specifically within the RC, and as seen in the analysis of recent cell walls, was more similar to the pattern seen in the RP region (Figures 3E–3H).

Based on the combined analyses of new cell walls and marked clones, we conclude that *RPL* regulates oriented tissue growth and establishes distinct growth patterns in the central and peripheral regions of the RZ.

### RPL Directly Binds to Key Genes that Regulate Meristem Function, Organ Patterning, and Growth

**Figure 4:**
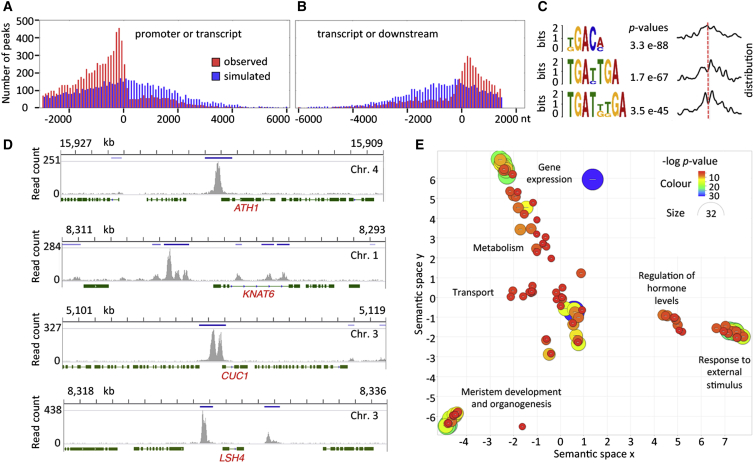
Genome-wide RPL Targets Identified by ChIP-Seq (A and B) Enrichment of RPL binding sites within promoter regions (A) and downstream regions (B) compared with transcribed regions, for the 2,917 high-confidence RPL candidate targets (Table S1). Red bars show the frequency of peak regions (overlap of peaks from three ChIP-seq replicates) centered at the indicated nucleotide positions relative to transcript start (A) or end (B); blue bars show how simulated peaks at random genome positions were distributed within the set of high-confidence RPL targets. Based on 10,000 simulations, the observed RPL binding sites were significantly enriched (p < 10^−4^) in both promoter and downstream regions compared with transcribed regions. (C) Enrichment for sequence motifs within 75 nt of the RPL binding sites analyzed in (A) and (B), detected using the MEME Suite (Bailey et al., 2009). Curves on the right show the frequency distribution of enriched motifs, relative to the center of the peak region (red line). (D) Representative raw ChIP-seq peaks (replicate 1 only) for control genes (*ATH1*, *KNAT6*) and two key organ boundary genes (*CUC1*, *LSH4*). Dark-blue bars show regions of overlap between peaks detected in three replicates (peak regions mentioned in A–C); green bars and lines are exons and introns, respectively; numbers above each graph show chromosome position in kilobases. All genes are oriented 5′ (left) to 3′ (right). (E) Semantic clustering (Supek et al., 2011) of GO categories enriched in the set of 2,917 candidate RPL targets (Table S2). The diameter and color of each circle reflect the p value for individual GO terms within the cluster; the broad terms used to describe each cluster are attributed to specific GO terms in Table S2. See also Figure S3; Tables S1 and S2.

As a transcriptional regulator, *RPL* is expected to affect patterns of growth indirectly through its downstream target genes. To reveal genes and processes regulated by *RPL* in the RZ, we first used chromatin immunoprecipitation (ChIP) sequencing (ChIP-seq) to detect loci bound by RPL within the inflorescence apex. As internal controls, we used genes previously reported to interact with *RPL* genetically or by ChIP, including close partners or repressors of *RPL* function, such as *BREVIPEDICELLUS* (*BP*), *POUND-FOOLISH* (*PNF*), *ARABIDOPSIS THALIANA HOMEOBOX GENE 1* (*ATH1*), *KNOTTED1-LIKE HOMEOBOX GENE 6* (*KNAT6*), *BLADE ON PETIOLE 1* (*BOP1*), and *BOP2* (Smith and Hake, 2003, Smith et al., 2004, Khan et al., 2012, Khan et al., 2015, Ragni et al., 2008, Khan et al., 2015), as well as genes that interact with *RPL* during flowering, floral organ, and fruit development, such as *LEAFY* (*LFY*), *AGAMOUS* (*AG*), *APETALA 1* (*AP1*), *SEPALLATA 3* (*SEP3*), *APETALA 2* (*AP2*), *SHATTERPROOF 1* (*SHP1*), *SHP2*, *FRUITFULL* (*FUL*), and *MIR156A*, *C*, and *E* (Lal et al., 2011, Roeder et al., 2003, Smaczniak et al., 2012, Andrés et al., 2015). Anti-GFP antibodies were used to pull down DNA bound by RPL-GFP expressed as a genomic fusion (*pRPL*:*RPL-GFP*) that mirrored the endogenous *RPL* expression and complemented the *rpl-1* mutant (Figure S1). ChIP-seq peaks with a false discovery rate of less than 0.001 and consistently detected in three RPL-GFP replicates but not in wild-type replicates were selected and associated with genes that contained a peak within 3 kb upstream and 1.5 kb downstream of their coding sequences (see examples in Figure 4D). From the list of genes that satisfied these conditions, we selected a set of 2,917 high-confidence candidates (Table S1) that showed a peak enrichment at least as high as the positive control gene with the lowest enrichment (*APETALA2*).

Within the high-confidence targets set, ChIP-seq peaks were depleted in transcribed regions but enriched in the immediately adjacent regions, as expected for the role of RPL as a transcriptional regulator (Figures 4A, 4B, and 4D). BLH proteins function with a KNOX homeodomain partner (Bellaoui et al., 2001), which is BP in the case of RPL (Smith and Hake, 2003). Accordingly, sequences in the vicinity of the peak summits were significantly enriched for short motifs containing TGAC/T (Figure 4C), similar to the binding sites previously described for BLH and KNOX proteins (Smith et al., 2002). Gene ontology (GO) analysis (Falcon and Gentleman, 2007) followed by semantic clustering (Supek et al., 2011) revealed clusters of highly enriched functional categories (Figure 4E and Table S2). As for other master regulatory genes (Kaufmann et al., 2009, Schiessl et al., 2014), the most highly enriched terms were related to transcriptional control (Table S1). The second most highly enriched cluster of GO terms corresponded to meristem functions, early organogenesis, and reproductive development, as detailed below. Additional sets of enriched terms were related to hormone metabolism and responses (particularly involving auxin, gibberellin, and jasmonic acid), ion and sugar transport, and responses to external stimuli (e.g., pathogens and light) (Figure 4E).

Genes in the “meristem development and organogenesis” cluster revealed direct links to many well-known players in shoot development. Reflecting the role of *RPL* in meristem establishment, its targets included genes involved in maintaining the stem cell niche: *SHOOT MERISTEMLESS*, *CLAVATA 1*, *A-TYPE RESPONSE REGULATOR 7* (*ARR7*), *ARR15*, and *ARGONAUTE 10* (Aichinger et al., 2012). Based on its antagonism with *ATH1*, *BOP1*, *BOP2*, and *KNAT6*, which are expressed at the boundary between lateral organs the stem, *RPL* has been proposed to oppose organ boundary development (Khan et al., 2015); accordingly, RPL interacted directly with the majority of known organ boundary genes, including *CUP-SHAPED COTYLEDONS 1* (*CUC1*), *CUC3*, *CUC*-repressing microRNAs (*miR164B* and *miR164C*), known downstream components of the *CUC* pathway *LATERAL ORGAN FUSION 1* (*LOF1*), *LOF2*, *LIGHT-DEPENDENT SHORT HYPOCOTYL 3* (*LSH3*), *LSH4*, and multiple members of the *LATERAL ORGAN BOUNDARIES* (*LOB*) *DOMAIN* family, including *LOB* and *JAGGED LATERAL ORGANS* (Žádníková and Simon, 2014, Hepworth and Pautot, 2015). Furthermore, the ChIP-seq results revealed links between *RPL* and a large number of genes involved in shoot organogenesis, including genes that control abaxial/adaxial identity, organ growth, cell cycle, cell-wall functions, and vascular development (Table S1). In summary, the ChIP-seq results placed *RPL* in a central hub connecting many of the key regulatory pathways in shoot development.

### RPL Promotes Rib Meristem Function by Antagonizing the Organ Boundary Gene *LSH4*

**Figure 5:**
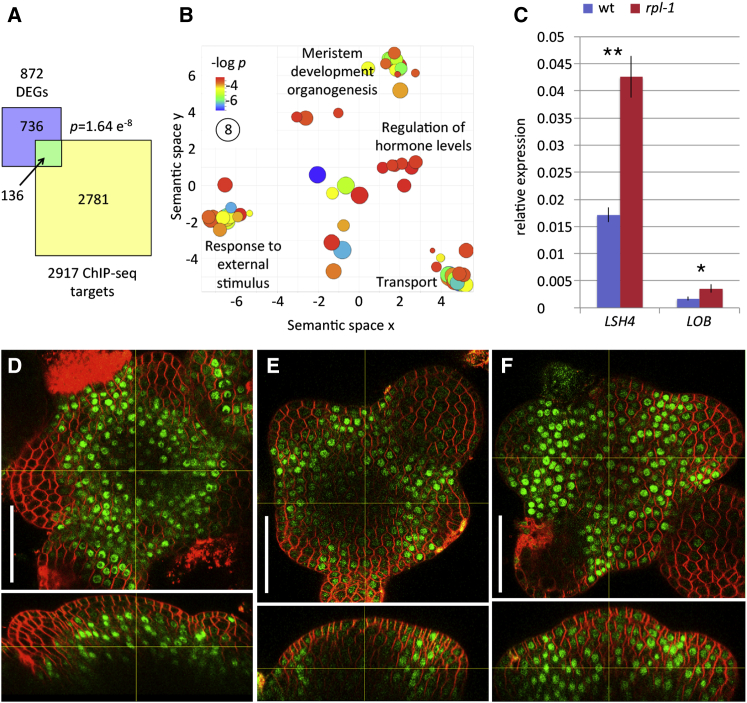
Genes Regulated by RPL Include the Boundary Gene *LSH4* (A) Overlap between ChIP-seq results and genes that were differentially expressed (DEGs) in wild-type and *rpl-1* inflorescence apices (Table S3). The overlap of both sets was larger than expected by chance (p = 1.64 × 10^−8^, Fisher's exact test). (B) Semantic clustering (Supek et al., 2011) of GO categories enriched in the set of 136 direct RPL target genes. The diameter and color of each circle reflect the p value for individual GO terms within the cluster; broad terms used to describe each cluster are attributed to specific GO terms in Table S4. (C) qRT-PCR measurement of *LSH4* and *LOB* expression levels in wild-type and *rpl-1* inflorescence apices. Bars and lines show means and SD (three biological replicates), with asterisks indicating significant difference (Student's t test; *LSH4*: ^∗∗^p < 0.01, *LOB*: ^∗^p < 0.05). (D–F) orthogonal views of confocal image stacks of inflorescence apices showing the expression pattern of *pRPL-RPL-GFP* (D), *pLSH4*:*LSH4-GFP* in wild-type (E), and *pLSH4*:*LSH4-GFP* in *rpl-1* (F). In each image, the yellow cross-hairs mark the same point in the top and side views. Scale bars, 50 μm. See also Tables S3 and S4.

Many of the target genes mentioned above are likely regulated by both *RPL* and its close homolog *PNF*, since these two genes function redundantly in meristem establishment and in the control of the floral transition. To narrow down the list of genes that could mediate the role of *RPL* in the RZ, we took advantage of the fact that some processes, such as stem elongation and fruit development, are preferentially affected in the *rpl* single mutant (Smith and Hake, 2003, Roeder et al., 2003, Byrne et al., 2003). To filter the ChIP-seq data for genes relevant to stem development, we looked for transcriptome changes in dissected inflorescence apices of *rpl-1* compared with the wild-type (Table S3). Although the majority of differences in mRNA abundance were expected to result from indirect, steady-state effects of *RPL*, the set of differentially expressed genes (DEGs) was significantly enriched for direct RPL targets based on ChIP-seq (p = 1.64 × 10^−8^, Fisher's exact test; Figure 5A). The 136 directly regulated targets included approximately equal numbers of upregulated (67) and downregulated genes (69) (Table S3), indicating that RPL can function as both a transcriptional activator and repressor. GO analysis of these genes showed clusters similar to those in the ChIP-seq experiments, highlighting meristem development and organogenesis, regulation of hormone levels, responses to hormones and external stimuli, and transport of ions and sugar (Figure 5B and Table S4). The meristem and organogenesis cluster included components of the core RPL regulatory module (*ATH1*, *KNAT6*), genes that regulate meristem function (*STM*, *ARR7*, *AGO10*, *HAM3*), and genes implicated in organ boundary development (*LSH4*, *LOB*) (Table S3).

Of the known regulators of shoot development present in the set of directly regulated targets, *LSH4* showed the most significant differential expression (Table S3). We next focused on this gene, considering that *LSH4* functions downstream of *CUC* genes, which control not only organ boundary development but also stem development (Vroemen et al., 2003, Hibara et al., 2006). The higher expression of *LSH4* in the mutant, seen in the transcriptome profiling, was verified by RT-PCR, and similar results were obtained for *LOB* (Figure 5C). To determine the spatial localization of LSH4, we used a genomic fusion with GFP (*pLSH4*:*LSH4-GFP*) to visualize the expression pattern in apical meristems. In wild-type apices we observed GFP around the base of floral buds and in the peripheral region of the RZ (Figure 5D), similar to the previously described expression pattern for *LSH4* (Takeda et al., 2011). In contrast, in the *rpl-1* mutant, the region of *pLSH4*:*LSH4-GFP* expression extended into the central region of the RZ (Figure 5E). Comparable results were obtained with the *pCUC1*:*CUC1-GFP* organ boundary reporter (Baker et al., 2005), confirming that *RPL* represses a suite of organ boundary genes in the RZ (Figure S4).

**Figure 6:**
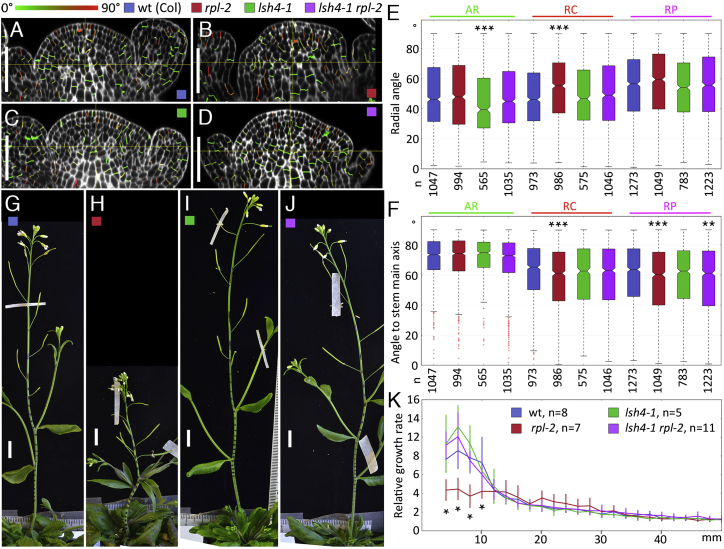
*LSH4* Expression Causes the *rpl* Defects in RZ Development and Stem Growth (A–D) Longitudinal sections through confocal image stacks of inflorescence apices stained by mPS-PI, overlaid with images of segmented cell facets detected as new walls and colored (color scale above A) according to their radial orientation as in Figure 2A. (A) Wild-type (Columbia); (B) *rpl-2*; (C) *lsh4-1*; (D) *rpl-2 lsh4-1*. (E and F) Boxplots showing the distribution of new wall radial angles (E) or angles to the main stem axis (F). Colors correspond to the genotypes indicated above (A and B). n indicates the number of new walls in each set (combined data from four apices for each genotype); asterisks indicate statistically significant differences (^∗∗^p < 0.01, ^∗∗∗^p < 0.001, Mann-Whitney test). (G–J) Inflorescences of wild-type Columbia (G), *rpl-2* (H), *lsh4-1* (I), and *lsh4-1 rpl-2* (J), 4 days after the first flower self-pollinated and black marks were placed on the stem at 2-mm intervals to track growth rates. (K) Relative growth of different stem regions, measured by tracking landmarks placed on the stem as in (G)–(J). The graph shows mean and SD; the number of replicates is indicated on the color legend for each genotype; asterisks indicate statistically significant differences relative to the wild-type (^∗^p < 0.01, Student's t test). The horizontal axis shows the original distance of landmarks to the apex, before growth. In the boxplots, the box extends from the lower to upper quartile values with a line at the median; whiskers extend to 1.5 times the interquartile range, and outlier points beyond the whiskers are shown in red. Scale bars, 50 μm (A–D) and 1 cm (G–J). See also Figure S6.

To test the functional relevance of *LSH4* repression by RPL, we crossed the *rpl-2* and *lsh4-1* mutants (both strong alleles in the Columbia accession) (Takeda et al., 2011, Roeder et al., 2003). Similar to our observations for *rpl-1*, cells in the RZ appeared less ordered in *rpl-2* than in wild-type apices (Figures 6A and 6B), with significant differences in the orientation of new walls in the central region (RC), relative to both the radial axes (Figure 6E) and the stem main axis (Figure 6F). We did not observe differences in the RC of the *lsh4-1* single mutant compared with the wild-type, although the mutant did show an increase in radially oriented divisions in the AR of the meristem (Figure 6E). In the double mutant *rpl-2 lsh4-1*, the RC was visibly more ordered than in *rpl-2* (Figure 6D) and the orientation of cell divisions was restored to the wild-type pattern in the RC, although not in the surrounding RP (Figures 6E and 6F). The defect in stem elongation of *rpl-2* was also suppressed in *rpl-2 lsh4-1* due to restoration of elongation rates close to the inflorescence apex (Figures 6G–6K), while defects in fruit development were not suppressed in the double mutant (Figure S5).

In conclusion, ectopic *LSH4* expression caused most of the defects in RZ function and stem growth seen in the *rpl* mutant. Mutations in *BOP1*, *BOP2*, *ATH1*, and *KNAT6* have been shown to restore all wild-type functions in the *rpl* mutant, including flowering time and fruit development, indicating that these genes function within the same central regulatory node as *RPL* (Khan et al., 2012, Khan et al., 2015, Ragni et al., 2008, Khan et al., 2015). In contrast, *lsh4-1* suppressed a subset of the *rpl-2* phenotypes, suggesting a more specialized role for organ boundary functions in the control of stem growth by *RPL*. In accordance with a role for *LSH4* downstream of the *BOP1*/*BOP2*/*ATH1*/*KNAT6* module, suppression of the *rpl-2* defects by *knat6-2* included restoration of *LSH4* repression and rescue of oriented divisions in the RZ (Figure S6). At the same time, the almost complete rescue of RZ function and stem growth in the *rpl lsh4* double mutant (Figure 6) suggests that if additional organ boundary genes are relevant to the control of RZ function by *RPL*, these genes function within a module that requires *LSH4* activity.

## Discussion

Our results provide insight into the 3D patterns of growth and cell division in the deep layers of the shoot apical meristem, a region crucial for the development of new stem tissues. We reveal that *RPL* controls RZ function not through the rate of cell growth and proliferation but by repressing organ boundary genes to allow the establishment of central and peripheral regions, which have characteristic patterns of oriented cell division.

Organ boundaries are considered regions of reduced growth (Hepworth and Pautot, 2015), so activation of boundary genes in the RZ could be expected to inhibit tissue growth (Žádníková and Simon, 2014, Hepworth and Pautot, 2015). However, our clonal analysis did not reveal reduced growth rates in tissues that expressed *LSH4*, i.e., in the RP region in the wild-type and *rpl-1*, or in the RC region of *rpl-1* (Figure 6). Instead, the most obvious change caused by *LSH4* was in the orientation of cell divisions. Ectopic *LSH4* expression in the *rpl* mutant may have narrowed the rib meristem because of the lower frequency of radial divisions observed in the RC region, where the rib meristem is initiated (Figures 2 and 6), or may have induced inappropriate radial divisions during subsequent growth of the rib meristem. Either way, within the region of the developing stem that overlaps the *RPL* expression domain (Smith and Hake, 2003) (Figure S3), the primary consequence of losing *RPL* function was not a reduction in overall growth, but a defect in establishing distinct central and peripheral regions. The reduced stem growth seen within a few millimeters of the apex in the *rpl* mutant is likely an indirect consequence of the early RZ defects. One possibility is that an abnormal or displaced boundary between the central and peripheral RZ might affect development of the stem vasculature and interfascicular fibers, which form at this boundary, are affected in the *rpl* mutant, and have been proposed to mechanically constrain stem elongation (Muñiz et al., 2008, Mele et al., 2003, Smith and Hake, 2003).

An important question is by which mechanism *RPL* and *LSH4* could affect oriented cell growth and division. Mechanical stress during tissue growth feeds back to influence the orientation of microtubule arrays and cell division (Hamant et al., 2008), so a possible mechanism would be that the visibly thicker cell walls of the rib meristem (e.g., Figures 2A and 6C) could impose mechanical constraints on the surrounding tissues. Alternatively, *RPL* and *LSH4* could modulate auxin signaling or transport. The orientation of cell divisions responds to auxin (Yoshida et al., 2014), perhaps through regulation of the molecular mechanism that orients the mitotic spindle, or perhaps indirectly by setting the direction of cell growth (Sassi and Traas, 2015). Furthermore, auxin transport is regulated at organ boundaries to create a low-auxin environment (Heisler et al., 2010, Wang et al., 2014, Hepworth and Pautot, 2015). A role in regulating auxin functions is also suggested by our ChIP-seq results showing direct interaction between RPL and multiple genes involved in auxin transport and signaling (Vanneste and Friml, 2009), e.g., *PIN-FORMED 5* (*PIN5*), *PIN6*, *LIKE AUXIN 1* (*LAX1*), *LAX3*, *AUXIN RESPONSIVE FACTOR 4* (*ARF4*), *ARF6*, *ARF8*, *ARF10*, *ARF11*, and *ARF17* (Table S1).

At first sight it could be expected that the rate of stem elongation would simply reflect the rate of cell growth and proliferation in the RZ, just as root elongation reflects the rate at which new cells are produced by the root meristem (Beemster and Baskin, 1998). Contrary to this expectation, our results emphasize the regulation of axial growth through orientation, rather than rates of cell growth and division. In an analogous way, it has been assumed that elongation of the vertebrate limb results from a proximodistal gradient of cell proliferation, but recent 3D imaging and mathematical modeling highlighted the role of oriented cell activities (Boehm et al., 2010). In addition to providing insight into the internal cell behavior required for growth of a 3D structure, our work opens the way to study and modify a developmental process that influences plant traits with key practical importance.

## Experimental Procedures

### Plant Material

Plants were grown on JIC *Arabidopsis* Soil Mix at 16°C under continuous light (100 μE). *Arabidopsis thaliana* Landsberg-*erecta* (L-*er*) and Columbia (Col) were used as wild-types; *rpl-1* (Roeder et al., 2003), *rpl-2* (Roeder et al., 2003), *lsh4-1* (Takeda et al., 2011), *knat6-2* (Ragni et al., 2008), *pCUC1*:*CUC1-GFP* (Baker et al., 2005), and *hsp18.2*:*Cre* (Sieburth et al., 1998) have been described. Transgenic lines were generated by floral dip transformation (Clough and Bent, 1998).

PCR primers used to create DNA constructs are listed in Supplemental Experimental Procedures. For construction of *pRPL*:*RPL-GFP*, *RPL* was amplified from Col genomic DNA and fused in-frame with sGFP(S65T) (Chiu et al., 1996), and cloned into pPZP222 (Hajdukiewicz et al., 1994). For construction of *pLSH4*:*LSH4-GFP*, *LSH4* was amplified from Col genomic DNA and the sGFP(S65T) cDNA was inserted in-frame at the end of the *LSH4* coding sequence before assembly into pCambia 1300 (CAMBIA). The *35S*:*loxCFPloxGFP* was created by Golden Gate cloning in the vector pAGM4723 (Addgene #48015) as described by Weber et al. (2011), using synthesized DNA (Lifetech) for the 35S promoter, *loxP* reverse, CFP-ER, 35S terminator, *loxP* reverse, GFP-ER, and the actin terminator (see Supplemental Experimental Procedures for sequences).

### Imaging and Image Analysis

Dissection and live imaging of inflorescence apices, including time-lapse experiments, and imaging of apices stained by the modified pseudo-Schiff propidium iodide (mPS-PI) method were performed as described previously (Serrano-Mislata et al., 2015, Truernit and Haseloff, 2008). For generation of Cre-*lox*P sectors, plants hemizygous for *hsp18.2*:*Cre* and *35S*:*loxCFPloxGFP* were heat-shocked by immersing their inflorescence apices in a water bath at 38.5°C for 70 s and returned to standard growth conditions for 3 days before dissection and live imaging.

For 3D segmentation, cell measurements, and matching cells at different time points, 3D_meristem_analysis was used (Serrano-Mislata et al., 2015), with additional scripts added to detect and analyze the 3D orientation of new cell walls, to landmark, align, and measure Cre-*lox*P sectors from different apices (Data S1).

### Chromatin Immunoprecipitation/High-Throughput Sequencing and Data Analysis

ChIP was performed on dissected inflorescence apices as described by Schiessl et al. (2014) (details in Supplemental Experimental Procedures). Six Illumina TruSeq ChIP-seq libraries (three *pRPL*: *RPL-GFP* replicates and three wild-type controls) were produced as described by Kaufmann et al. (2009) and sequenced (50-bp single-end reads) using a HiSeq 2500 (Rapid-Run mode) as described by the manufacturer (Illumina). Reads from three replicate treatments and three replicate controls were aligned against the TAIR10 Col-0 reference sequence with Bowtie2 (v2-2.1.0; Langmead and Salzberg, 2012), data were sorted and indexed with SAMtools (Li et al., 2009), and MACS 2.0.10 (Feng et al., 2012) was used to call peaks and calculate fold enrichments and q values, comparing the combined replicates with combined controls. For selection of peaks that were consistently detected across replicates, peak calling with MACS 2.0.10 was applied to individual replicates and overlapping peak regions were accepted if they had q values of 10^−3^ or lower in each *RPL*:*RPL-GFP* replicate, were not detected in any of the negative controls, and the overlapping region was at least 50 nt long. After this filtering step, peaks were attributed to gene models within 3 kb upstream or 1.5 kb downstream of the corresponding coding sequence, without intervening coding sequences. For peak overlaps and association to gene models, the script Overlap_MACS2_files.py was used (details in annotated source code, associated gene models, and annotation tables in Data S2). ChIP-seq data were visualized using the Integrative Genomics Viewer (Robinson et al., 2011).

To analyze the distribution of peaks within genes we used the script *peak_statistics.py*, which also includes details of the Monte Carlo method used to estimate the p value for the hypothesis that these frequencies correspond to a random distribution of peaks within genes (Data S2). For detection of enrichment for sequence motifs, MEME-ChIP (http://meme-suite.org/tools/meme-chip) (Bailey et al., 2009) was used in discriminative mode, comparing the sequences around observed peaks with a control set of sequences around a 10-fold larger number of random peaks; both sets were produced with script *peak_sequences.py* (Data S2). To test for overrepresented GO terms, we used the hypergeometric test of the GOstats package (Falcon and Gentleman, 2007) with the org.At.tair.db annotation package (Gentleman et al., 2004), and Revigo (Supek et al., 2011) was used to cluster enriched terms.

Raw and processed data have been deposited in the NCBI Gene Expression Omnibus (Edgar et al., 2002) under accession number GEO: GSE78727.

### Transcriptome Analysis

Ten micrograms of RNA was extracted from inflorescence apices of wild-type L-*er* and *rpl-1* (three replicates each) with Trizol (Sigma) and purified using Qiagen RNAeasy columns (Qiagen) according to the manufacturer's instructions. AGRONOMICS1 arrays (Affymetrix) were hybridized with labeled cDNA following the manufacturer's instructions. To detect DEGs, we used the affylmGUI package (http://bioinf.wehi.edu.au/affylmGUI/about.html) to run LIMMA (Linear Models for MicroArray data) (Smyth, 2005) using a chip description format (CDF) file for the AGRONOMICS1 array and TAIR10 (Müller et al., 2012). Raw and processed data have been deposited in the NCBI Gene Expression Omnibus (Edgar et al., 2002) under accession number GEO: GSE78511.

### qPCR

qRT-PCR was performed as published by Schiessl et al. (2012) (details in Supplemental Experimental Procedures).

### Measurements of Stem Growth

Plants were grown as described above; when the first flower self-pollinated, ink dots were manually placed on the stem at 2-mm intervals and photographed next to a ruler. After a further 4 days of growth, the stems were photographed again. The ink marks and positions on the rules were landmarked manually on the images using the Point Picker plugin of Fiji (Schindelin et al., 2012). Distances between landmark coordinates were measured, graphs were plotted, and Mann-Whitney U tests and Student's t tests were performed using standard functions in matplotlib (http://matplotlib.org), Python 2.7, and Scientific Python (http://www.scipy.org).

## Author Contributions

Conceptualization, S.B. and R.S.; Investigation, S.B., A.S.-M., and M.B.; Resources, S.F.; Software, Formal Analysis, and Data Curation, R.S.; Writing – Original Draft, R.S.; Writing – Review & Editing, S.B., S.F., A.S.-M., M.B., and R.S.; Funding Acquisition, R.S; Supervision, R.S.

## Accession Numbers

Raw and processed data have been deposited in the NCBI Gene Expression Omnibus under accession numbers GEO: GSE78727 and GSE78511.

## Supplemental Information

Document S1. Supplemental Experimental Procedures and Figures S1–S6 Document S1. Supplemental Experimental Procedures and Figures S1–S6

Table S1. High-Confidence RPL ChIP-Seq Targets; Genes Considered as Positive Controls Due to Known Interactions with RPL Are Marked in Red, Related to Figure 4 Table S1. High-Confidence RPL ChIP-Seq Targets; Genes Considered as Positive Controls Due to Known Interactions with RPL Are Marked in Red, Related to Figure 4

Table S2. Enrichment of GO Terms—Biological Process—in the Set of High-Confidence RPL ChIP-Seq Targets, Related to Figure 4 Table S2. Enrichment of GO Terms—Biological Process—in the Set of High-Confidence RPL ChIP-Seq Targets, Related to Figure 4

Table S3. Genes Differentially Expressed between Wild-Type and *rpl-1* Inflorescences Table S3. Genes Differentially Expressed between Wild-Type and rpl-1 Inflorescences

Table S4. Enrichment of GO Terms—Biological Process—in the Overlap between High-Confidence ChIP-Seq Targets and Genes that Showed Differential Expression between *rpl-1* and Wild-Type, Related to Figure 5 Table S4. Enrichment of GO Terms—Biological Process—in the Overlap between High-Confidence ChIP-Seq Targets and Genes that Showed Differential Expression between rpl-1 and Wild-Type, Related to Figure 5

Data S1. Annotated Source Code and Instructions for Installation and Use of Scripts for Image Analysis, Related to Experimental Procedures Data S1. Annotated Source Code and Instructions for Installation and Use of Scripts for Image Analysis, Related to Experimental Procedures

Data S2. Annotated Source Code, Instructions for Installation and Use of Scripts for ChIP-Seq Analysis, and Gene Annotations Used, Related to Experimental Procedures Data S2. Annotated Source Code, Instructions for Installation and Use of Scripts for ChIP-Seq Analysis, and Gene Annotations Used, Related to Experimental Procedures

Document S2. Article plus Supplemental Information Document S2. Article plus Supplemental Information
