# Supplementary material for: Control of Oriented Tissue Growth through Repression of Organ Boundary Genes Promotes Stem Morphogenesis
Source: Dev Cell. 2016 Oct 24;39(2):198–208. doi: 10.1016/j.devcel.2016.08.013 (PMC5084710; doi:10.1016/j.devcel.2016.08.013)
Supplement: Document S1. Supplemental Experimental Procedures and Figures S1–S6 [file mmc1.pdf]

Developmental Cell, Volume *39*

## Supplemental Information

# Control of Oriented Tissue Growth

# through Repression of Organ Boundary Genes

# Promotes Stem Morphogenesis

**Stefano Bencivenga, Antonio Serrano-Mislata, Max Bush, Samantha Fox, and Robert Sablowski**

**Supplemental Information**

**Inventory:**

**Figure S1:** Recent divisions in inner meristem layers detected by cell tracking and corresponding new cell walls detected based on mPS-PI signal. Overlap of confocal images and segmentation images of tracked cells and new cell walls; related to Figure 1.

**Figure S2:** Differences in cell division orientation in the rib zone are consistent across individual apices. Boxplots, statistical analysis of division angles and confocal images of inflorescence apices, related to Figure 2.

**Figure S3** *pRPL:RPL-GFP* complements *rpl-1* and is expressed in the the RZ. Confocal images of inflorescence apices and fruit expressing *pRPL:RPL-GFP* and images of wild-type, *rpl* mutant and complemented plants; related to Figure 4.

**Figure S4:** The boundary marker *pCUC1:CUC1-GFP* is ectopically expressed in the RZ of *rpl-1*. Confocal images of inflorescence apices, related to Figure 6.

**Figure S5:** The *lsh4-1* mutation did not suppress the replum phenotype of *rpl-2* mutant. Scanning electron micrographs of developing fruits and histogram of replum width in wild-type, *rpl*, *lsh4* and *rpl lsh4* mutants; related to Figure 6.

**Figure S6:** Suppression of *rpl-2* by *knat6-2* includes restoration of *LSH4* repression and of cell division orientation in the rib zone. Related to Figure 6.

**Supplemental Table legends**

**Supplemental Data File legends**

**Supplemental Experimental Procedures**

**Supplemental References**

**Figure S1**: Recent divisions in inner meristem layers detected by cell tracking and corresponding new cell walls detected based on mPS-PI signal. Related to Figure 1.

The orthogonal views correspond to image stacks of a wild-type Arabidopsis inflorescence apex; the yellow cross-hairs mark the same point in the top and side views; size bars: 50 µm. A, B: tracking of cell divisions in the live meristem imaged at 0h (A) and 24h later (B); cells that could be tracked are shown in the same color in the two images; green asterisks indicate cells of layers 2 or 3 that divided. C, D: Matching of cells from live imaging at 0h (C) and imaged by mPS-PI at 24h (D); matching cells are shown in the same color and asterisks indicate cells that divided. E, F: images corresponding to E, F, but showing only the FM4-64 signal for the live image (E) and showing cell facets detected as recent walls (F); asterisks indicate the new walls corresponding to the cell divisions indicated in A-D.

Figure S1

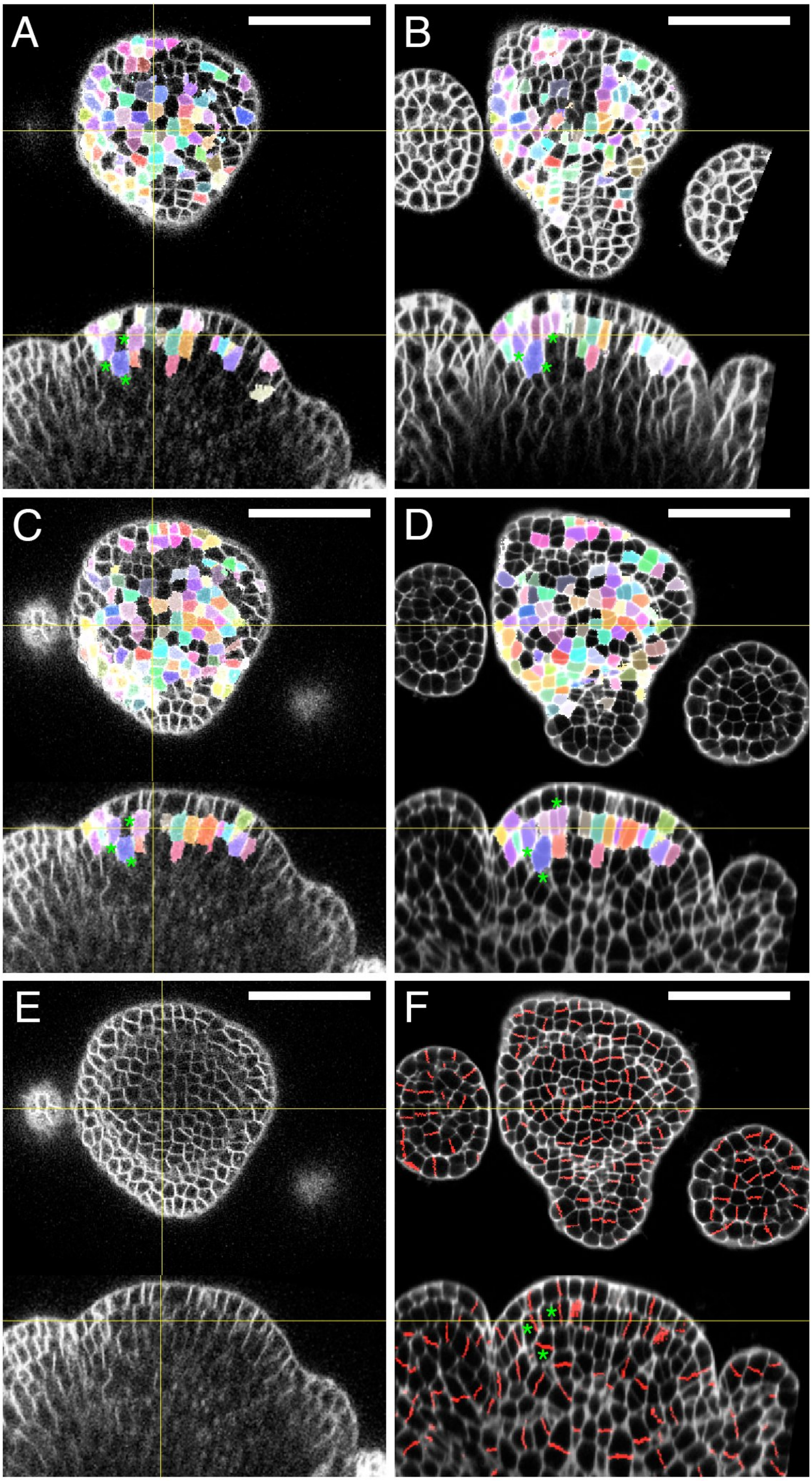

**Figure S2:** Differences in cell division orientation in the rib zone are consistent across individual apices. Related to Figure 2.

A: Analysis of the orientation of new cell walls in six wild-type (L-*er*) and four *rpl-1* apices were analysed. In the boxplots, the box extends from the lower to upper quartile values, with a line at the median, and whiskers extend to 1.5 times the interquartile range. The Kruskal-Wallis test *p*-values are for the null hypothesis that all apices have the same median orientation of cell divisions. The Mann-Whitney test *p*-values are for the null hypothesis that a particular replicate has the same median as the combined replicates for the other genotype (i.e., each wt was compared with the combined *rpl* data, and each *rpl* was compared with the combined wt data). AR, RC and RP correspond to the meristem regions shown in Figure 2C.

B, C: : Orthogonal views of confocal image stacks of inflorescence apices stained by mPS-PI. In each image, the yellow cross-hairs mark the same point in the top and side views. B: three apices each of wild-type (L-*er* and Col). C: three apices each of two different *rpl* alleles in the L-*er* and Col backgrounds (*rpl-1* and *rpl-2*, respectively). Note the less organized RZ (encircled region) in the *rpl* mutants compared to wt controls.

Figure S2

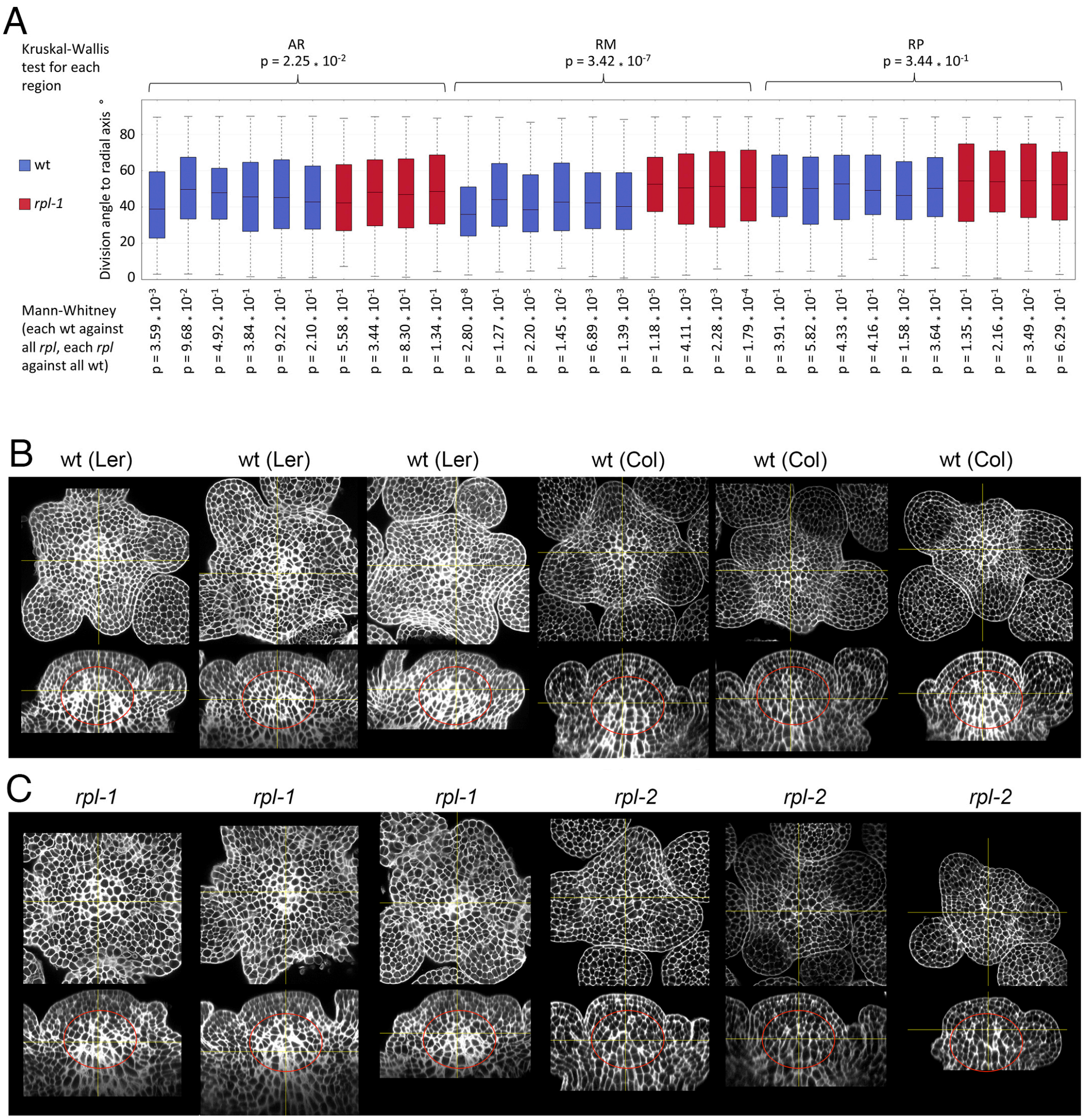
A
Kruskal-Wallis test for each region
AR $p = 2.25 \cdot 10^{-2}$
RM $p = 3.42 \cdot 10^{-7}$
RP $p = 3.44 \cdot 10^{-1}$
wt
rpl-1
Division angle to radial axis °
80
60
40
20
0
Mann-Whitney (each wt against all rpl, each rpl against all wt)
$p = 3.59 \cdot 10^{-3}$
$p = 9.68 \cdot 10^{-2}$
$p = 4.92 \cdot 10^{-1}$
$p = 3.84 \cdot 10^{-1}$
$p = 9.22 \cdot 10^{-1}$
$p = 2.10 \cdot 10^{-1}$
$p = 5.58 \cdot 10^{-1}$
$p = 3.44 \cdot 10^{-1}$
$p = 8.30 \cdot 10^{-1}$
$p = 1.34 \cdot 10^{-1}$
$p = 2.80 \cdot 10^{-8}$
$p = 1.27 \cdot 10^{-1}$
$p = 2.20 \cdot 10^{-5}$
$p = 1.45 \cdot 10^{-2}$
$p = 6.89 \cdot 10^{-3}$
$p = 1.39 \cdot 10^{-3}$
$p = 1.18 \cdot 10^{-5}$
$p = 4.11 \cdot 10^{-3}$
$p = 2.28 \cdot 10^{-3}$
$p = 1.79 \cdot 10^{-4}$
$p = 3.91 \cdot 10^{-1}$
$p = 5.82 \cdot 10^{-1}$
$p = 4.33 \cdot 10^{-1}$
$p = 4.16 \cdot 10^{-1}$
$p = 1.58 \cdot 10^{-2}$
$p = 3.64 \cdot 10^{-1}$
$p = 1.35 \cdot 10^{-1}$
$p = 2.16 \cdot 10^{-1}$
$p = 3.49 \cdot 10^{-2}$
$p = 6.29 \cdot 10^{-1}$
B
wt (Ler)
wt (Ler)
wt (Ler)
wt (Col)
wt (Col)
wt (Col)
C
rpl-1
rpl-1
rpl-1
rpl-2
rpl-2
rpl-2

**Figure S3**:

*pRPL:RPL-GFP* complements *rpl-1* and is expressed in the the RZ. Related to Figure 4.

A: confocal images of *pRPL:RPL-GFP rpl-1* inflorescence apex showing expression in the subapical region of the meristem; the yellow cross-hairs mark the same point in the orthogonal views. B: expression in the replum of *pRPL:RPL-GFP rpl-1* silique.

C-E: Arabidopsis plants at the stage when the third silique has elongated; C: wild type (Landsberg-*erecta*); D: *rpl-1*; E: *RPL:RPL-GFP rpl-1*. Scale bar: 50 µm (A, B), 6mm (C-E).

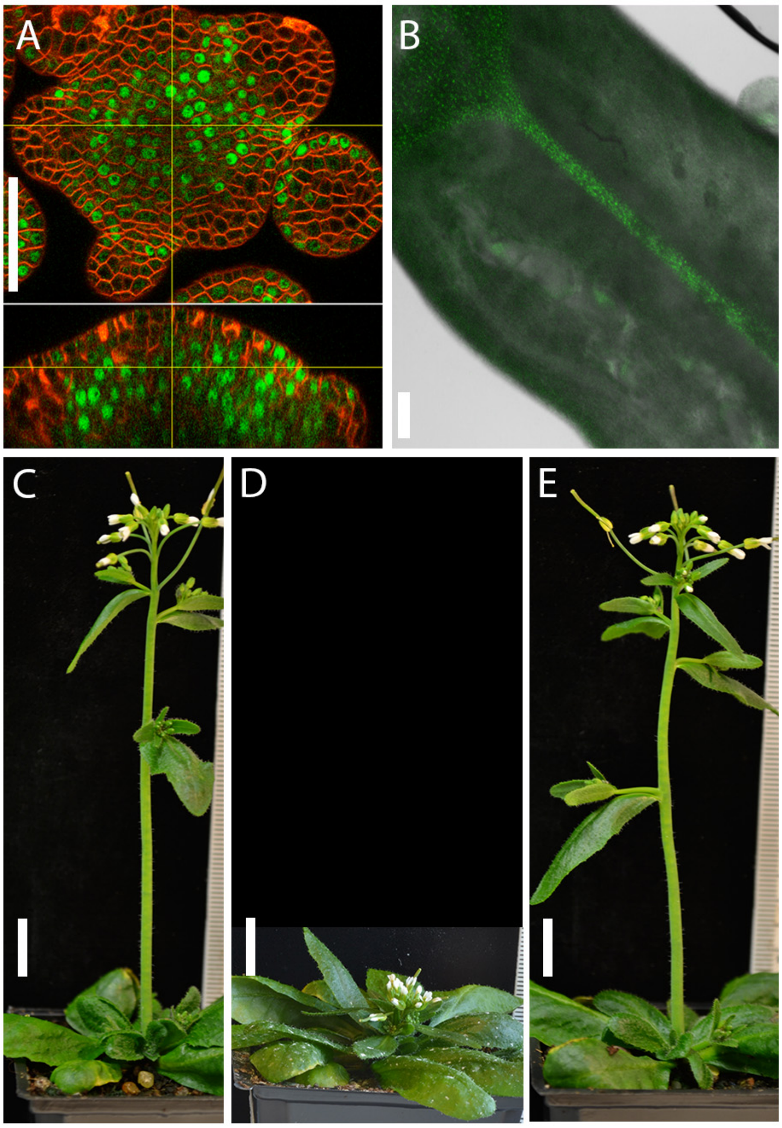

**Figure S4**: the boundary marker *pCUC1:CUC1-GFP* is ectopically expressed in the RZ of *rpl-1*. Related to Figure 6.

Orthogonal views of confocal image stacks showing *pCUC1:CUC1-GFP* expression in wild-type (A) and *rpl-1* (B) inflorescence apices; in each image, the yellow cross-hairs mark the same point in the top and side views. Scale bar: 50 µm

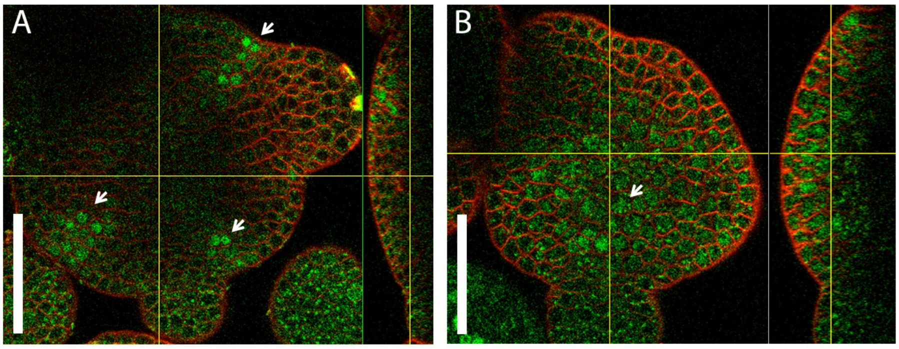

**Figure S5**: the *lsh4-1* mutation did not suppress the replum phenotype of *rpl-2* mutant. Related to Figure 6.

A-D: SEM images of developing siliques, showing the valves (V) and the replum (R) in the wild type (A), *rpl-2* (B), *lsh4-1* (C) and *rpl-2 lsh4-1* (D); white lines and black boxes show measurements of replum width. E: average (bars) and standard deviation (lines) for replum width in the genotypes shown in A-D. Scale bars: 20 µm.

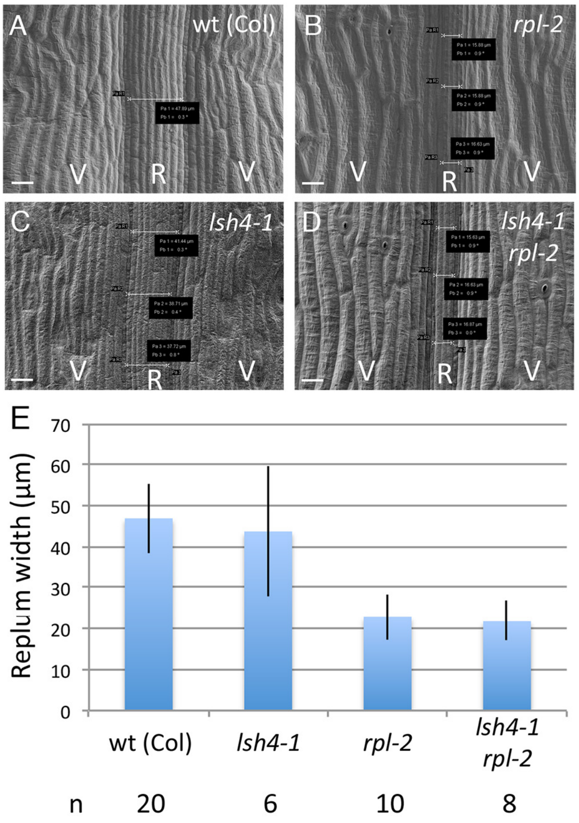

**Figure S6**: Suppression of *rpl-2* by *knat6-2* includes restoration of *LSH4* repression and of cell division orientation in the rib zone. Related to Figure 6.

A-B: representative inflorescences of wild type Columbia (A) and *knat6-2 rpl-2* (B) after the first 2-3 flowers matured. C-D: longitudinal sections through confocal image stacks of inflorescence apices of wild-type (Columbia)(C) and *knat6-2 rpl*-2 (D), with new walls colored according to radial orientation as in Figure 2A (compare with wt and *rpl-2* in Figure 6 A,B); E: expression of *LSH4* in inflorescence apices of *rpl-2*, wt (Columbia) and *knat6-2 rpl-2*, measured by qRT-PCR (average and standard deviation of 3 biological replicates per genotype; asterisk indicates significant difference to wt, $p < 0.05$, *t*-test). F-G: boxplots showing the distribution of new wall angles to the radial axis (F) and main stem axis (G) (compare with wt and *rpl-2* in Figure 6 E,F); colors correspond to the genotypes indicated in E; n indicates the number of new walls in each set (combined data from 4-5 apices for each genotype); asterisks indicate statistically significant differences ($p < 0.001$, Mann-Whitney test). Scale bars: 1 cm (A, B), 50 µm (C, D). In the boxplots, the box extends from the lower to upper quartile values with a line at the median; whiskers extend to 1.5 times the interquartile range and outlier points beyond the whiskers are shown in red.

Figure S6

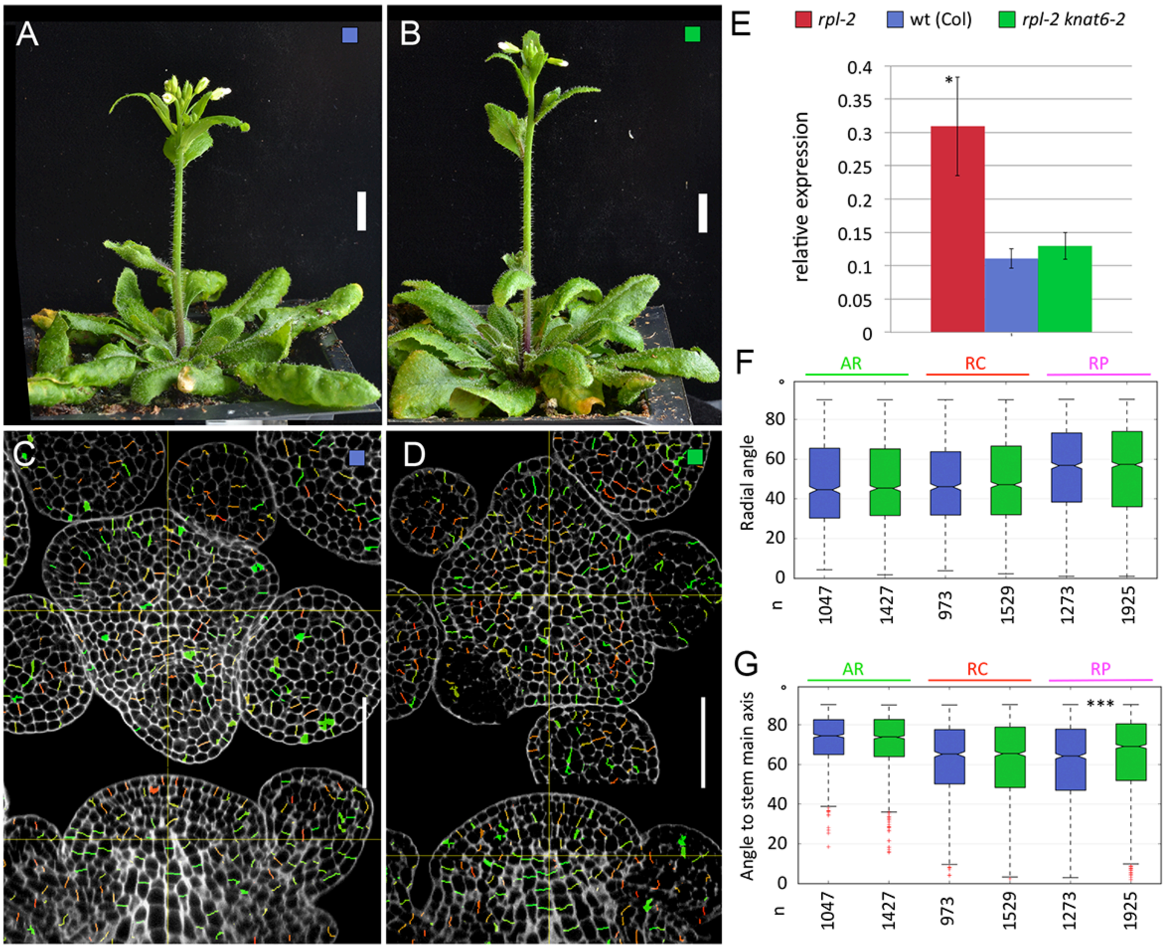

A
B
C
D
E
rpl-2
wt (Col)
rpl-2 knat6-2
relative expression
0.4
0.35
0.3
0.25
0.2
0.15
0.1
0.05
0
*
F
AR
RC
RP
Radial angle
°
80
60
40
20
0
n
1047
1427
973
1529
1273
1925
G
AR
RC
RP
Angle to stem main axis
***
°
80
60
40
20
0
n
1047
1427
973
1529
1273
1925

**Supplemental Table legends**

**Table S1**: High-confidence RPL ChIP-seq targets; genes considered as positive controls due to known interactions with RPL are marked in red (Related to Figure 4).

**Table S2**: Enrichment of GO terms (biological process) in the set of high-confidence RPL ChIP-seq targets (Related to Figure 4).

**Table S3:** Genes that were differentially expressed between wild-type and *rpl-1* inflorescences. A: Differentially expressed genes also present in the list of high-confidence ChIP-seq targets (Table S1). B: Differentially expressed genes absent from the list of high-confidence ChIP-seq targets in Table S1. (Related to Figure 5).

**Table S4:** Enrichment of GO terms (biological process) in the overlap between high-confidence ChIP-seq targets and genes that showed differential expression between *rpl-1* and the wild type. (Related to Figure 5).

**Supplemental Data File legends**

**Supplemental Software 1**: zip file containing annotated source code and instructions for installation and use of scripts used for image analysis; related to Experimental Procedures.

**Supplemental Software 2**: zip file containing annotated source code, instructions for installation and use of scripts for ChIP-seq analysis and gene annotations used; related to Experimental Procedures.

**Supplemental experimental procedures**

***DNA sequences used for constructs***

To produce *35S:loxCFPloxGFP,* the full sequence of the synthetic DNA inserted into pAGM4723 (Addgene) is shown below, with each component color-coded as follows:

35S promoter, loxP reverse, CYPET-ER, 35S terminator, GFP-ER, Actin terminator, Golden Gate scar sequences.

5'gtcaacatggtggagcacgacactctggtctactccaaaaatgtcaaagatacagtctcagaagatcaaagggctattgagactt
ttcaacaaaggataatttcgggaaacctcctcggattccattgcccagctatctgtcacttcatcgaaaggacagtagaaaaggaag
gtggctcctacaaatgccatcattgcgataaaggaaaggctatcattcaagatctctctgccgacagtggtcccaaagatggacccc
cacccacgaggagcatcgtggaaaaagaagaggttccaaccacgtctacaaagcaagtggattgatgtgacatctccactgacgt
aagggatgacgcacaatcccactatccttcgcaagacccttcctctatataaggaagttcatttcatttggagaggacacgctactga
cctaataacttcgtatagcatacattatacgaagttatattaagggttgaatgaaaactaatctttttctctttctcatcttttcacttctc
ctatcattatcctcggccgaattcggaggtgtgagcaagggagaggaactgttcggcggcatcgtgcccatcctggtggagctggag
ggcgacgtgaacggccacaagttcagcgtgagcggcgagggcgagggcgacgccacctacggcaagctgaccctgaagttcatct
gcaccaccggcaagctgcccgtgccctggcccaccctggtgaccaccctgacctggggcgtgcagtgcttcagccggtaccccgac
cacatgaagcagcacgacttcttcaagagcgtgatgcccgagggctacgtgcaggagcggaccatcttcttcaaggacgacggca
actacaagacccgggccgaggtgaagttcgagggcgacaccctggtgaaccggatcgagctgaagggcatcgacttcaaggagg
acggcaacatcctgggccacaagctggagtacaactacatcagccataacgtgtacatcaccgccgacaagcagaagaacggcat
caaggccaacttcaaggcccggcacaacatcaccgacggcagcgtgcagctggccgaccactaccagcagaacacccccatcggc
gacggccccgtgatcctgcccgacaaccactacctgagcacccagagcgccctgagcaaggaccccaacgagaagcgggaccat
atggtgctgctggagttcgtgaccgccgccggcatcacccacggcatggacgaactgtacaaaggtggccatgatgagctttaagct
tctctagctagagtcgatcgacaagctcgagtttctccataataatgtgtgagtagttcccagataagggaattagggttcctatagg

gtttcgctcatgtgttgagcatataagaaacccttagtatgtatttgtatttgtaaaatacttctatcaataaaatttctaattcctaaaa ccaaaatccagtactaaaatccagatcgctgacctaataacttcgtatagcatacattatacgaagttatattaagggttgaatgaa aactaatctttttctctttctcatcttttcacttctcctatcattatcctcggccgaattcggaggtatggtgagcaagggcgaggagct gttcaccggggtggtgcccatcctggtcgagctggacggcgacgtaaacggccacaagttcagcgtgtccggcgagggcgagggc gatgccacctacggcaagctgaccctgaagttcatctgcaccaccggcaagctgcccgtgccctggcccaccctcgtgaccaccctg acctacggcgtgcagtgcttcagccgctaccccgaccacatgaagcagcacgacttcttcaagtccgccatgcccgaaggctacgtc caggagcgcaccatcttcttcaaggacgacggcaactacaagacccgcgccgaggtgaagttcgagggcgacaccctggtgaacc gcatcgagctgaagggcatcgacttcaaggaggacggcaacatcctggggcacaagctggagtacaactacaacagccacaacg tctatatcatggccgacaagcagaagaacggcatcaaggtgaacttcaagatccgccacaacatcgaggacggcagcgtgcagct cgccgaccactaccagcagaacacccccatcggcgacggccccgtgctgctgcccgacaaccactacctgagcacccagtccgccc tgagcaaagaccccaacgagaagcgcgatcacatggtcctgctggagttcgtgaccgccgccgggatcactctcggcatggacga gctgtacaagggtggccatgatgagctttaagcttgctctcaagatcaaaggcttaaaaagctggggttttatgaatgggatcaaag tttcttttttttcttttatatttgcttctccatttgtttgtttcatttccctttttgttttcgtttctatgatgcacttgtgtgtgacaaactctctg ggtttttacttacgtctgcgtttcaaaaaaaaaaaccgctttcgttttgcgttttagtcccattgttttgtagctctgagtgatcgaattg atgcctctttattccttttgttccctataatttctttcaaaactcagaagaaaaaccttgaaactctttgcaatgttaatataagtattgt ataagatttttattgatttggttattagtcttacttttgctacctccatcttcacttggaactgatattctgaatagttaaagcgttacatg tgttccattcacaaatgaacttaaactagcacaaagtcagatattttaagatcgcaccatttcgct 3’.

To amplify the *RPL* genomic sequence described in Experimental Procedures, the primers ATCTGGATCCGTATCGATAAGCGGATCCTTATTT and AAGGTCTAGATCTTTGGACCTACAAAATCATGTAGAAACTG were used.

To amplify sGFP(S65T) for in-frame fusion with *RPL* at the BamHI site, the primers used were: ATCTGGATCCATGGTGAGCAAGGGCGAGGA and ATCTGGATCCTTACTTGTACAGCTCGTCCA.

To amplify genomic *LSH4* fragments for fusion with sGFP(S65T), the following primer pairs were used: GTGGTCTCAGGAGTTTTACCATGCCTCCTTGCTC and GTGGTCTCTCACCATTAGGGCTACTTGAAATCGC; GTGGTCTCAGCTTCATTAAGTCATGAGTAAGATGT and GTGGTCTCAAGCGTCACACGCTCTCACCGTCTC.

To amplify sGFP(S65T) for in-frame fusion with *LSH4* at the *Bsa*I site, the primers used were: GTGGTCTCAGGTGGCATGGTGAGCAAG and GTGGTCTCAAAGCTTACTTGTACAGCT.

***Chromatin Immunoprecipitation (ChIP)***

*pRPL:RPL-GFP rpl-1* and wild-type L-*er* control plants were used. For each replicate, 1.3-1.5 g of inflorescence apices were fixed under vacuum for 20 min in 35 ml of ice-cold fixation buffer (0.4 M sucrose, 10mM Tris pH 8, 1 mM EDTA pH 8.5, 1% formaldehyde, 100 µM PMSF). 100 µM glycine was added for 10 min on ice to stop cross-linking, followed by two washes with water. The tissue was blotted dry and frozen in liquid nitrogen, Nuclei were purified (49) and re-suspended in 1 ml of sonication buffer (500 mM Hepes, 150 mM NaCl, 5 mM MgCl2, 10% TRITON X-100) with half a tablet of protease inhibitor cocktail complete Mini, EDTA-free (Roche). After sonication in a Bioruptor sonicator at 4°C (2 x 5 min high power level with 30 sec on/30sec off cycles), producing an average fragment size of 500 bp, the samples were centrifuged, the supernatant was mixed with 500 ul of immunoprecipitation buffer (0.5M Hepes, 150 mM NaCl, 5 mM MgCl2 , 10% TRITON X-100, 1 mg/ml BSA) and 25 µl of anti-GFP µMACS Microbeads (Milteyi Biotec). After 30 min on ice, the samples were loaded on a µ Column (Milteyi Biotec) equilibrated with immunoprecipitation buffer, then placed into a magnetic µMACS separator (Milteyi Biotec). After washing twice with 400 µl and twice with 200 µl of immunoprecipitation buffer, twice

with 200 µl TE (100 mM Tris pH 8, 10 mM EDTA pH 8), the DNA was eluted once with 20 µl and twice with 50 µl of preheated (96° C) elution buffer (50 mM Tris pH8, 10 mM EDTA, 50 mM DTT and 1% SDS). 100 µl of TE-buffer and 9 µl of 25 mg/ml Proteinase K (Sigma) were added to the eluted samples and to the input control samples. Crosslinking was reverted by incubation at 37°C overnight, addition of 9 µl of 25 mg/ml Proteinase K and 8 h incubation at 65° C. After phenol-chloroform extraction, precipitation with ethanol overnight at -20° C, and washing with 70% ethanol, the air-dried DNA was re-suspended in 100 µl PCR-grade water (Roche), purified using a PCR purification Kit (18104, Qiagen) and stored at -80° C.

***Analysis of ChIP-seq peaks (using Supplemental Software 2)***

The protocol below was used after calling ChIP-seq peaks with MACS2 as described in the Experimental Procedures to filter ChIP-seq peaks for reproducibility across replicates, to associate peaks to gene models, to analyze the distribution of peaks within associated genes and to select input sequences for MEME-ChIP.

**1. Installation**

To use the scripts, expand the folder *Peaks_analysis* (Supplemantal Software 2) and place it on the Desktop. The scripts were written in Python 2.7.3 on an Apple computer running MacOS X 10.9.4 - changes may be needed to install and run them on a different platform. Dependencies are Numerical Python (http://www.numpy.org), matplotlib (http://matplotlib.org) and Tcl/tk (https://www.python.org/download/mac/tcltk/).

**2. Filtering peaks for reproducibility across replicates and attributing gene models**

ChIP-seq reads from three replicate treatments and three replicate controls were aligned against the genome and peaks called as described in Experimental Procedures). To calculate fold enrichments and *q*-values, the combined replicates were compared with the combined controls using MACS 2.0.10 (Feng et al., 2012); this generated the files RPL-GFP_fused_peaks.narrowPeak and wt_fused_peaks.narrowPeak.gz. In addition, MACS 2.0.10 was applied to individual replicates to select for consistency across replicates; this generated files RPL-GFP1_peaks.narrowPeak, RPL-GFP2_peaks.narrowPeak, RPL-GFP3_peaks.narrowPeak, wt1_peaks.narrowPeak, wt2_peaks.narrowPeak, wt3_peaks.narrowPeak. These files are deposited at NCBI (http://www.ncbi.nlm.nih.gov/geo/query/acc.cgi?&acc=GSE78727) and need to be placed together in a local folder for the analysis below.

To filter for consistency across replicates, the script Overlap_MACS2_files.py is called by double-clicking on the shell script ~/Peaks_analysis/shell_scripts/Overlap_MACS2_files.sh. The script searches for overlaps between peak regions listed in selected replicates (GFP1_peaks.narrowPeak, RPL-GFP2_peaks.narrowPeak, RPL-GFP3_peaks.narrowPeak files), and accepts the overlaps if they are absent from all controls, have q-values of a specified value or lower in each replicate, and the overlapping region is at least of a specified length.

After the filtering step above, peaks are attributed to gene models and an annotated table is produced. For this, tables with gene coordinates and with gene annotations are selected - see ~/Peaks_analysis/TAIR10_tables/TAIR10_AGI_location.txt and TAIR10_functional_descriptions.txt. Based on these tables, peaks are associated

with gene models within specified distances upstream and downstream of the transcribed regions, without intervening genes.

Inputs are selected interactively:

path to folder containing narrowPeak files

path to table containing gene positions

path to table containing gene annotations

Parameters are set directly on the script (using a suitable editor such a IDLE):

n is the minimum width in nucleotides for accepting the overlap between MACS peaks (default n = 50)

prom is the length in nucleotides for the upstream regulatory region for each gene model (used to associate peaks with genes); default prom = 4000

utr is the length in nucleotides for the downstream regulatory region for each gene model (used to associate peaks with genes) default utr = 1500

chromosomes specifies chromosome names and lengths in nucleotides

default chromosomes = np.array([['Chr1', 30500000], ['Chr2', 19700000], ['Chr3', 23500000], ['Chr4', 18600000], ['Chr5', 27000000]])

fc_cutoff sets the position of bedgraph line for fold change; default fc_cutoff = 3

q_cutoff = 3 sets cutoff q value for overlapping peaks from different replicates; default q_cutoff = 3

Outputs are:

A tab-delimited text table with the MACS2 statistics, gene models and annotation associated with each overlapping peak region (named Overlapping_peaks_AGI.txt, saved in the same folder as the narrowPeak files).

A tab-delimited text table with the nucleotide position of the center of each overlapping peak region (named "Peak_positions.txt" and saved in the same folder as the narrowPeak files).

bedgraph files (which can be opened in the IGV browser) for peak fold changes (Overlapping_peaks_fold_change.bedgraph) and for peak q-values (Overlapping_peaks_q_values.bedgraph), both placed in the same directory as the narrowPeak files.

**3. Analyzing the distribution of peaks within associated genes**

To statistically analyze peak locations within genes, the script peak_statistics.py is called by double-clicking on the shell script ~/Peaks_analysis/shell_scripts/Peak_statistics.sh. This script interactively selects the file with the position of overlapping peak regions and the file with associated gene models (both produced in step 2 above), the table with gene coordinates (~/Peaks_analysis/TAIR10_tables/TAIR10_AGI_location.txt), and asks for the number of replicates used when scoring the positions of simulated, random peaks.

The script scores the frequency of observed peak regions centered on the transcribed, upstream and downstream regions, then uses a Monte Carlo method to estimate the *p*-value for the hypothesis that these frequencies correspond to a random distribution of peaks within genes. A tab-delimited text table with the results is produced, in addition to histograms showing the frequency of observed and simulated (random) peaks at different distances to the start and end of transcribed regions (Peak_position_statistics.txt, Peaks_histogram_upstream.png,

Peaks_histogram_downstream.png, all saved in the same folder as the selected file with peak positions).

Set parameters chromosomes, which specify chromosome names and lengths in nucleotides; default values are:

chromosomes = np.array([['Chr1', 30500000], ['Chr2', 19700000], ['Chr3', 23500000], ['Chr4', 18600000], ['Chr5', 27000000]])

**4. Selecting input sequences for MEME-ChIP**

To detect enrichment for sequence motifs within overlapping peak regions, MEME-ChIP (http://meme-suite.org/tools/meme-chip) was used in discriminative mode, comparing the sequences around observed peaks with a control set of sequences around a ten-fold larger number of random peaks (Experimental Procedures). To produce both sets of sequences, the script peak_sequences.py is called by double-clicking on the shell script ~/Peaks_analysis/shell_scripts/Peak_sequences.sh.

The script interactively selects the file with the positions of overlapping peak regions and the file with associated gene models (both produced in step 2 above), the table with gene coordinates (~/Peaks_analysis/TAIR10_tables/TAIR10_AGI_location.txt), then asks for the size of the region to include on each side of the center of each peak region. A folder is also selected with chromosome sequences. This is not included here and must be downloaded from public databases (e.g. TAIR, http://www.arabidopsis.org) - one file for each chromosome, with a single sequence in FASTA format; the files must be named "TAIR10_chr1.fas", "TAIR10_chr2.fas" etc.

To produce a control set of sequences for discriminative MEME, the script generates random peak positions in each chromosome. The size of the control set (how many times larger than the observed set) is set interactively.

The outputs are a list of sequences flanking the centers of observed peak regions (Peak_sequences.txt) and a list of sequences flanking random peak centers (Random_sequences.txt), both in FASTA format, saved in the same folder containing the input file with the positions of peak regions.

Set parameters chromosomes, which specify chromosome names and lengths in nucleotides; default values are:

chromosomes = np.array([['Chr1', 30500000], ['Chr2', 19700000], ['Chr3', 23500000], ['Chr4', 18600000], ['Chr5', 27000000]])

***Quantitative reverse transcription PCR***

Quantitative reverse transcription-polymerase chain reaction (qRT-PCR) was performed with the LightCycler 480 System and SYBR Green I (Roche) and the primers listed below. Enrichment from ΔCt values (Ct immunoprecipitated DNA – Ct input DNA) using the 2-ΔΔCt method as described (Livak and Schmittgen, 2001). Data were normalized to *ACTIN2* amplified with the primers below. For each biological replicate, the average value of three technical replicates was used; two-tailed Student's t-tests were used to test for statistical significance of differences between sets of biological replicates.

The following primers were used: for *LSH4*, ACCAATTCGGCAAGACTAAGGTTC and AGCAGCTCTAAGACGGCCAATG; for *LOB*, TGCGTCGGAGCCATCTCTTATC and AGTCAGCATTAGCTGCGTCGAG; for *ACTIN 2*, GCACCCTGTTCTTCTTACCG and AACCCTCGTAGATTGGCACA.

*Confocal imaging*

mPS-PI (modified pseudo-Schiff-propidium iodide) imaging was modified from (Truernit et al., 2008). Inflorescence tips (terminal 0.5 cm) were fixed for 15 min each in 15%, 30%, 50%, 70%, 85%, 95% and 100% ethanol. After 16h in ethanol, all floral buds older than stage 3 (Smyth et al., 1990), the samples were rehydrated through the same ethanol series, washed in water and incubated at 37°C overnight in alpha-amylase (Sigma) 0.3 mg/mL in phosphate buffer 20 mM pH7.0, 2 mM NaCl, 0.25 mM $CaCl_2$. The apices were then rinsed in water, treated 30 min in 1% periodic acid, washed twice in water and incubated 2 h in Schiff-PI reagent, cleared with chloral hydrate solution and mounted in Hoyer's medium (Truernit et al., 2008), before imaging with a Zeiss LSM780 confocal microscope with excitation at 488 nm, emission filters set to 572-625 nm, using a X40/1.0 dipping objective; image resolution was 0.42 X 0.42 X 0.5 µm.

*Image analysis (using Supplemental Software 1)*

The protocol below explains how to install and use a set of Python scripts and Fiji macros used to analyze the orientation of recent cell divisions in plant tissues and for the analysis of marked clones in three dimensions.

These scripts are based on a previously published set of scripts for 3D segmentation, cell measurements and cell tracking (Serrano-Mislata et al., 2015). Only new scripts are described below; for details of published scripts, please see the corresponding instructions (Serrano-Mislata et al., 2015).

Fiji (Schindelin et al., 2012) is used to visualize and interact with processed images (e.g. to select landmarks on the image) and Fiji macros are used to facilitate this. It is assumed that the user is familiar with Fiji in general and in particular with the plugins 3D

Viewer (Schmid et al., 2010) and Pointpicker. For instructions on how to use these, please refer to:

http://fiji.sc/Getting_started

http://fiji.sc/3D_Viewer

http://bigwww.epfl.ch/thevenaz/pointpicker/

**1. Installation**

To use the scripts, expand the folder *Rib_zone_analysis* (Supplemental Software 1) and place it on the Desktop (other locations will work, but will require editing the paths mentioned below). To read and write images in the correct location, the path to images must be edited in the Fiji macro *~/Rib_meristem_analysis/Fiji_macros/confocal_to_TIF.ijm*; open the script with Fiji and edit the path attributed to the variable “ANALYSIS_PATH” in the first line; use the path leading to the Rib_meristem_analysis folder (e.g. in my case "/Users/Author/Desktop/Rib_meristem_analysis/")

The scripts require Numerical Python (http://www.numpy.org), Scientific Python (http://www.scipy.org), matplotlib (http://matplotlib.org) and SimpleITK (http://www.simpleitk.org). To install these dependencies using MacOS X 10, open a Terminal session and type after the “$” sign (you will need an administrator password):

sudo easy_install numpy

sudo easy_install scipy

sudo easy_install matplotlib

sudo easy install SimpleITK

To check that the required dependencies are in place, open a Python session in Terminal (type “python”) and try to import the packages by typing the lines below after the “>>>” prompt; if no error message appears, you will be ready to run the image analysis scripts.

import numpy

import scipy

import matplotlib

import SimpleITK

For the Fiji macros, install the latest version of Fiji (http://fiji.sc/Fiji). Make sure that the plugin 3D Viewer is listed in the plugins menu; if not, download and install (http://fiji.sc/3D_Viewer). The plugin Pointpicker is also required; download (http://bigwww.epfl.ch/thevenaz/pointpicker/) and install. Specific lookup tables (LUT) are also required to visualize the images: go to Applications, open the Fiji folder (it may be necessary to use ctrl click, “Show package contents”), then copy into the folder “LUT” the following files:

~ */Rib_meristem_analysis/LUTs/seg.lut*

~ */Rib_meristem_analysis/LUTs/walls_hm.lut*

After copying the LUTs, re-start Fiji and check that the LUTs appear in the pull-down menu *Image>Lookup Tables.*

The scripts and macros were written in Python 2.7.3 on an Apple computer running MacOS X 10.9.4 and Java 6 - changes may be needed to install and run them on a different platform.

**2. Setting up the images table**

The scripts are managed through a table that specifies where the input and output images are located, and what scripts are needed for the analysis. This allows the analysis to be customized. Another advantage of using the images table is that it serves as a database of all images analyzed.

To set up the images table, use *~/Rib_meristem_analysis/processed_images/images_table.csv* as the template (~ indicates the path leading to the *Rib_meristem_analysis* folder in the computer where the scripts are installed). The scripts will look for this table path and name, so both must remain unchanged.

The table provided already contains lines to process test images. These are meant just as examples of how to fill in the table and will not work with the scripts because the corresponding image folders are absent from *~/Rib_meristem_analysis/processed_images/*. If you would like to create the required folders to test run the scripts, the test images can be found in https://open-omero.nbi.ac.uk (username "shared", password "Op3n-4cc0unt" - note that all non-image files, such as Metadata, data tables, list of landmark coordinates, will be found as attachments of the image ending with "_seg.tif")

You can use e.g. Excel or TextEdit to add new lines to the table for new images to be analyzed, but make sure that the table is saved as comma-separated values (.csv).

The following fields are filled in for each new image stack:

- Your name (optional)

- Notebook number and page for experiment (optional)

- Date (optional)

- Path to Rib_meristem_analysis folder (required - in the example lines, this is set to /Users/Author/Desktop/ Rib_meristem_analysis/processed_images/; please change this to the actual path leading to the files in the computer where the scripts will be used)

- Folder name (required - this is a unique identifier for the images, which is called rootname in the rest of this protocol)

Additional columns track the progress through the scripts mentioned below, which will be called sequentially by clicking on the shell script *~/Rib_meristem_analysis/shell scripts/Rib_meristem_analysis*. Whenever marked "0", the image has not yet been processed by that script; after processed, the column is automatically marked "1". If a script needs to be run again on an image, just change the value for that script back to "0" before running the batch script again. If a script is not relevant to the analysis of a particular image, the corresponding position on the table should be marked with any other character, e.g. "x".

**3. Selecting and cropping confocal images**

The image analysis starts with separate stacks for each confocal channel and a metadata file with information such as voxel sizes. To create the stacks, use the Fiji macro *~/Rib_meristem_analysis/Fiji_macros/confocal_to_TIF.ijm* (select using

Fiji>Plugins>Macros>Run). Follow the instructions to open the confocal image, specify the rootname for the images, crop the image and split the channels. The macro creates a folder within *~/Rib_meristem_analysis/processed_*images, named with the given rootname and containing TIF stacks for each of the channels selected: rootname_R.tif for FM4-64/PI channel, rootname_G.tif for GFP channel, if used. In addition, a Metadata.txt file is created, with information about the file name, path to original confocal stack and voxel sizes. The Metadata file will also be updated with the specific parameters used by each subsequent script used to analyse the images. The folders with test images are provided with the output of c *confocal_to_TIF.*ijm, i.e. Metadata, _R.tif and _G.tif files.

**4. Landmarking**

Landmarks need to be added as reference points to select different regions (such as meristem and buds), to find the main axis of the apex etc. These landmark files need to be given specific names to be called by the Python scripts. For this, run the Fiji macro landmarks_3D.ijm (select using Fiji>Plugins>Macros>Run). Follow the instructions on the screen; typically the image to open is rootname_R.tif.

When processing images to measure cell wall orientations (see below), the main axis (e.g. of the stem) must be defined. For this, select 2 points must be selected in the order: top (e.g. inflorescence meristem summit), and bottom (e.g. center of stem near the bottom of the image). Save landmarks as rootname_axis.

When processing images to analyze Cre-loxP sectors, landmarks are placed at the boundaries of floral buds to find the main axis and to subsequently align and superimpose multiple images. For this, select a point at the center of the boundary

between the meristem and a floral bud, for the 5 youngest buds. Save landmarks as rootname_boundaries.

For the sectors analysis, it is also necessary to manually landmark the cells in each sector using the Fiji macro *mark_GFP_sectors.ijm* (Fiji>Plugins>Macros>Run). Follow the instructions on the terminal to open the image of cell outlines (rootname_R.tif) and the image with GFP signal (rootname_sectors_G.tif). Adjust its brightness of the GFP image on the B&C window, click on "Apply", say "Yes" to applying to the whole stack, then click on the "OK" button in the window "Please adjust brightness and contrast". You will see a Point Picker window with the images fused and a yellow square on the top left corner. Scroll up and down the image, choose a sector and click only once in each cell of the sector; you will see crosses appearing on the marked cells. Before moving to the next sector, click on the square in the corner - this is important to create a marker point to separate each sector. Save points as rootname_sectors (use the button with a page icon on the Fiji menu bar), then click on "Click OK when done".

**5. Segmentation and cell measurements**

Once the rootname_R.tif, rootname_G.tif (if applicable), Metadata.txt and landmarks (.points) files are in place, the images can be segmented and the cells measured. For segmentation, measurement of cell volumes and position relative to the main axis and to the apex, the scripts *watershed_segmentation.py, cell_data_table.py* and *rib_zone.py* are called. Details of these scripts, input parameters and output are given in the instructions for 3D_meristem_analysis (Serrano-Mislata et al., 2015). To run these scripts and the new cell wall scripts

below, set the corresponding columns in the images table to "0" and double click on the shell script *~/Rib_meristem_analysis/shell scripts/Rib_meristem_analysis.*

To visualize segmented images, open them in Fiji and select the LUT “seg” on the pull down menu *Images>Lookup tables*. If the colors are not displayed correctly, it is necessary to re-set the LUT; for this, run the Fiji macro reset_LUT (select using Fiji>Plugins>Macros>Run).

**6. Finding new walls and their orientation**

After running *watershed_segmentation.py, cell_data_table.py* and *rib_zone.py*, it is possible to identify newly deposited cell walls and their orientation by calling the scripts:

*new_walls.py*

This script detects newly deposited cell walls in images of tissue stained by mPS-PI (Truernit and Haseloff, 2008), assuming that mPS-PI signal is proportional to wall thickness. A new wall is flagged when two neighboring cells share the same wall as their wall with lowest intensity. To correct for diminishing intensity with increasing depth in the confocal stack, the mPS-PI image in each plane is normalized using the average signal within the segmented cells. The normalized cell wall intensities are then corrected for the bias introduced by the fact that walls parallel to the imaging plane appear weaker; for this, a corrective function is obtained by plotting all wall intensities as a function of their angle to the imaging plane. The corrected cell wall intensities are added to the walls data table and an image of new walls is saved.

Inputs are the mPS-PI confocal stack (_R.tif) and the corresponding segmented image (_seg.tif). If available, an image of cell walls and the corresponding walls data table are read, otherwise they are created.

Default parameters are: cs = 20 (size of the image cropped around each cell wall during processing); wmin = 165 (minimum number of voxels in accepted cell walls); wmax = 2000 (maximum number of voxels in accepted cell walls). If needed, these parameters can be changed directly on the script with a standard script editor.

Outputs are the walls data csv table containing corrected cell wall intensities and with new walls marked, and a 16 bit TIF image stack of the new walls.

*cell_wall_orientation.py*

This script takes an image of newly deposited cell walls (produced by the script "new_walls.py") and calculates the orientation of each new wall in relation to the image main axis. "Angle to the main axis" is the angle between the given vector for the image main axis and the vector normal to plane best fitting the wall; "Angle to the radial axis" is the angle between the vector normal to best fitting plane and a vector perpendicular to the main axis that crosses the wall's center of mass.

Input files are the segmented image, corresponding image of cell walls, image or landmarks for the main axis, cell data table and cell walls table. The calculated angles are added to the walls data table (produced by the script "new_walls.py") and the cell data tables. Images are produced in which the new walls are given values proportional to the angles to the main axis or radial axis (heat map images"_new_wall_angles_main_axis.tif" and"_new_wall_angles_radial_axis.tif").

Images are also saved in which the two cells flanking each new wall are given values proportional to the angles to the main axis or radial axis (heat map images"_new_cell_angles_main_axis.tif" and"_new_cell_angles_radial_axis.tif"). Default parameters are: cs = 20 (size of the image cropped around each cell wall during processing) bar_min = 0 (minimum values for angle in heat map images) bar_max = 90 (maximum values for angle in heat map images). If necessary, these parameters can be changed directly on the script with a standard script editor.

To visualize the heat map images, open in Fiji and choose the LUT "walls_hm" on the pull-down menu *Image>Lookup Tables.* To visualize the image of segmented cell walls, open in Fiji and select the LUT “seg” on the pull down menu *Images>Lookup tables*; if the colors are not displayed correctly, run the Fiji macro reset_LUT (select in Fiji>Plugins>Macros>Run).

**7. Analysis of Cre-loxP sectors**

To detect Cre-loxP sectors and their 3D orientation, it is necessary first to landmark the organ boundaries, manually mark the cells in each sector, segment and measure the cells (see steps 4 and 5 above). After this is done, make sure that the columns for the scripts below are set to "0". Select only the images that you want to merge if different genotypes are compared, each group of images has to be overlapped and analyzed in a different run. After the images table is set up and saved, run the shell script *~/Rib_meristem_analysis/shell scripts/Clonal_analysis.* This will call the following scripts:

*cell_layers.py*

This script attributes cells to tissue layers. This is necessary because the centers of mass of epidermal cells are used to produce an outline of the superimposed apices with marked sectors. This script is described in detail in the instructions for 3D_meristem_analysis (Serrano-Mislata et al., 2015).

*sectors_merge.py*

This script will save in each rootname folder an image (rootname_sector_landmarks.tif), in which dots labeled with a unique number are placed in the coordinates of the landmarks found in the file rootname_sectors.txt. The centers of epidermal cells are also marked to produce an outline of the apex. A similar image is produced in which the same landmark dots are labelled with sector numbers. A table is produced (rootname_landmarks_data.csv), listing the landmarks, their sector numbers, coordinates, distance to the apex and main axis, and measurements of the corresponding cells listed in the table (rootname_cell_data.csv) produced by *cell_data_table.py*.

In addition, three new images and a table are placed in the path *~/Rib_meristem_analysis/processed_images/*. In the image Merged_landmarks.tif, each of the rootname_sector_landmarks.tif image was rotated to align the main axis of the stem with the central axis of the image (running vertically through the centre), shifted to place the meristem summit point on plane 10 and rotated around the main axis to align the bud landmarks found in the file rootname_boundaries.points (bud P0 is placed at position 12 o'clock and older buds are aligned clockwise).

Merged_landmarks.tif is a similar overlap of all the aligned

rootname_sector_landmarks.tif images. Merged_boundaries.tif is another overlap of aligned images, but showing the positions of the landmarks placed on the bud boundaries in each image (to check how well the alignment worked). The table Merged_sectors_data.csv lists all the landmarks in Merged_landmarks.tif, the rootname of the image from which they originated, their coordinates, and the corresponding data from each of the rootname_cell_data.csv tables.

Inputs are:

images_table.csv; segmented image (rootname_seg.tif); image of main axis of the stem (rootname_main_axis.tif, produced by script rib_zone.py); file with coordinates of landmarks placed on each cell in sectors (rootname_sectors.txt); file with coordinates of landmarks placed on bud boundaries (rootname_boundaries.points)

Default parameters are:

radius = 2 (size in micrometers of sphere used to label landmarks on image); cs = 20 (defines the size of the image cropped around each sector during processing); sm = 1000 (defines value to label summit point; has to be more than the total number of objects in the merged sectors image).

*sectors_analyse.py*

This script uses the coordinates of sector landmarks in the merged image produced by *sectors_merge.py* to calculate the main axis of each sector and its orientation. To calculate the vector of the main axis, the linalg.svd (single value decomposition) function of Numpy is used. To calculate the size of the main axis, a plane perpendicular to the vector is placed on the center of mass of the sector landmarks; the distance between each landmark and the plane is calculated, and the length of

the main axis on each side of the plane equals the maximum landmark distance for that side. The orientation of the sector main axes is calculated as angle to the main axis (central z on the merged images) and to a vector perpendicular to the main axis and crossing the center of mass of the sector landmarks.

Images are produced of the sector landmarks, labeled with the corresponding sector number (Sector_landmarks.tif), of the corresponding sector axes (Sector_axes.tif) and of the sector axes overlapped with sector landmarks, to verify that the axes have been calculated correctly (Sector_axes_and_landmarks.tif). A table is also produced (Sectors_analysis.csv) with the coordinates for the center of mass, main axis extremities, main axis orientation and size.

In addition, the script produces the images "Sectors_landmarks_radial.png", Sectors_landmarks_top.png ", "Sector_3D_axes_radial.png", "Sector_3D_axes_top.png". These correspond to top projections (along the z axis) and radial projections of the landmark and axis images. To make the radial projection, each sector/axis was projected onto a plane containing its center of mass and the main image axis; sectors/axes on the right and left sides of the vertical projection are placed respectively on the right and left sides of the radial projection.

Finally, a lut table is saved, listing the RGB colors attributed to each sector/axis on the projections. This lut table can be used imported by Fiji (Plugins/LUT_editing/LUT importer) to visualize the landmarks and axes in the images Sector_landmarks.tif, Sector_axes.tif and Sector_axes_and_landmarks.tif.

Inputs are:

table "Merged_sectors_data.csv" (produced by sectors_merge.py); image "Merged_sectors.tif" (produced by sectors_merge.py); text file "Merged_images_metadata.txt" (produced by sectors_merge.py)

Default parameters are:

sm = 1000 (value attributed to summit point when aligning images; must match the value used in sectors_merge.py); radius = 2 (size in micrometers of sphere used to label landmarks on images); cs = 20 (defines the size of the image cropped around each sector during processing)

After running these scripts for one set of images, remember to save the files with a different name or move them to a different folder, or they will be overwritten by the subsequent run.

**Supplemental References**


Feng, J., Liu, T., Qin, B., Zhang, Y. & Liu, X. S. 2012. Identifying ChIP-seq enrichment using MACS. *Nature Protocols,* 7**,** 1728-1740.

Livak, K. J. & Schmittgen, T. D. 2001. Analysis of relative gene expression data using real-time quantitative PCR and the 2(T)(-Delta Delta C) method. *Methods,* 25**,** 402-408.

Schindelin, J., Arganda-Carreras, I., Frise, E., Kaynig, V., Longair, M., Pietzsch, T., Preibisch, S., Rueden, C., Saalfeld, S. & Schmid, B. 2012. Fiji: an open-source platform for biological-image analysis. *Nat. Methods,* 9**,** 676-682.

Schmid, B., Schindelin, J., Cardona, A., Longair, M. & Heisenberg, M. 2010. A high-level 3D visualization API for Java and ImageJ. *BMC Bioinformatics,* 11**,** 274.

Serrano-Mislata, A., Schiessl, K. & Sablowski, R. 2015. Active control of cell size generates spatial detail during plant organogenesis. *Curr. Biol.,* 25**,** 2991-2996.

Smyth, D. R., Bowman, J. L. & Meyerowitz, E. M. 1990. Early flower development in Arabidopsis. *Plant Cell,* 2**,** 755-67.

Truernit, E., Bauby, H., Dubreucq, B., Grandjean, O., Runions, J., Barthelemy, J. & Palauqui, J.-C. 2008. High-resolution whole-mount imaging of three-dimensional tissue organization and gene expression enables the study of phloem development and structure in Arabidopsis. *Plant Cell,* 20**,** 1494-1503.

Truernit, E. & Haseloff, J. 2008. A simple way to identify non-viable cells within living plant tissue using confocal microscopy. *Plant Methods,* 4**,** 15.
