# Supplementary material for: Control of Oriented Tissue Growth through Repression of Organ Boundary Genes Promotes Stem Morphogenesis
Source: Dev Cell. 2016 Oct 24;39(2):198–208. doi: 10.1016/j.devcel.2016.08.013 (PMC5084710; doi:10.1016/j.devcel.2016.08.013)
Supplement: Data S1. Annotated Source Code and Instructions for Installation and Use of Scripts for Image Analysis, Related to Experimental Procedures [file mmc6.zip › Rib_zone_analysis/Instructions/Image_processing_instructions.docx]

Bencivenga et al.

**Supplementary Software 1 - Image analysis**

The protocol below explains how to install and use a set of Python scripts and Fiji macros used to analyze the orientation of recent cell divisions in plant tissues and for the analysis of marked clones in three dimensions.

These scripts are based on a previously published set of scripts for 3D segmentation, cell measurements and cell tracking [1]. Only new scripts are described below; for details of published scripts, please see the corresponding instructions [1].

Fiji [2] is used to visualize and interact with processed images (e.g. to select landmarks on the image) and Fiji macros are used to facilitate this. It is assumed that the user is familiar with Fiji in general and in particular with the plugins 3D Viewer [3] and Pointpicker. For instructions on how to use these, please refer to:

<http://fiji.sc/Getting_started>

<http://fiji.sc/3D_Viewer>

<http://bigwww.epfl.ch/thevenaz/pointpicker/>

**1. Installation**

To use the scripts, expand the folder *Rib_zone_analysis* and place it on the Desktop (other locations will work, but will require editing the paths mentioned below). To read and write images in the correct location, the path to images must be edited in the Fiji macro *~/Rib_meristem_analysis/Fiji_macros/confocal_to_TIF.ijm*; open the script with Fiji and edit the path attributed to the variable “ANALYSIS_PATH” in the first line; use the path leading to the Rib_meristem_analysis folder (e.g. in my case "/Users/Author/Desktop/Rib_meristem_analysis/")

The scripts require Numerical Python (http://www.numpy.org), Scientific Python (http://www.scipy.org), matplotlib (http://matplotlib.org) and SimpleITK (http://www.simpleitk.org). To install these dependencies using MacOS X 10, open a Terminal session and type after the “$” sign (you will need an administrator password):

sudo easy_install numpy

sudo easy_install scipy

sudo easy_install matplotlib

sudo easy install SimpleITK

To check that the required dependencies are in place, open a Python session in Terminal (type “python”) and try to import the packages by typing the lines below after the “>>>” prompt; if no error message appears, you will be ready to run the image analysis scripts.

import numpy

import scipy

import matplotlib

import SimpleITK

For the Fiji macros, install the latest version of Fiji (<http://fiji.sc/Fiji>). Make sure that the plugin 3D Viewer is listed in the plugins menu; if not, download and install (http://fiji.sc/3D_Viewer). The plugin Pointpicker is also required; download (<http://bigwww.epfl.ch/thevenaz/pointpicker/>) and install. Specific lookup tables (LUT) are also required to visualize the images: go to Applications, open the Fiji folder (it may be necessary to use ctrl click, “Show package contents”), then copy into the folder “LUT” the following files:

*~ /Rib_meristem_analysis/LUTs/seg.lut*

*~ /Rib_meristem_analysis/LUTs/walls_hm.lut*

After copying the LUTs, re-start Fiji and check that the LUTs appear in the pull-down menu *Image>Lookup Tables.*

The scripts and macros were written in Python 2.7.3 on an Apple computer running MacOS X 10.9.4 and Java 6 - changes may be needed to install and run them on a different platform.

**2. Setting up the images table**

The scripts are managed through a table that specifies where the input and output images are located, and what scripts are needed for the analysis. This allows the analysis to be customized. Another advantage of using the images table is that it serves as a database of all images analysed.

To set up the images table, use *~/Rib_meristem_analysis/processed_images/images_table.csv* as the template (*~* indicates the path leading to the *Rib_meristem_analysis* folder in the computer where the scripts are installed). The scripts will look for this table path and name, so both must remain unchanged.

The table provided already contains two lines, which are meant just as examples of how to fill in the table and will not work with the script because the folder with images is absent from *~/Rib_meristem_analysis/processed_images/*. If you would like to create the required folder to test run the scripts, the images can be found in https://open-omero.nbi.ac.uk (username “shared”, password “Op3n-4cc0unt” - note that all non-image files, such as Metadata, data tables, list of landmark coordinates, will be found as attachments of the image ending with “_seg.tif”)

You can use e.g. Excel or TextEdit to add new lines to the table for new images to be analysed, but make sure that the table is saved as comma-separated values (.csv).

The following fields are filled in for each new image stack:

- Your name (optional)

- Notebook number and page for experiment (optional)

- Date (optional)

- Path to Rib_meristem_analysis folder (required - in the example lines, this is set to /Users/Author/Desktop/ Rib_meristem_analysis/processed_images/; please change this to the actual path leading to the files in the computer where the scripts will be used)

- Folder name (required - this is a unique identifier for the images, which is called rootname in the rest of this protocol)

Additional columns track the progress through the scripts mentioned below, which will be called sequentially by clicking on the shell script *~/Rib_meristem_analysis/shell scripts/Rib_meristem_analysis*. Whenever marked “0”, the image has not yet been processed by that script; after processed, the column is automatically marked “1”. If a script needs to be run again on an image, just change the value for that script back to “0” before running the batch script again. If a script is not relevant to the analysis of a particular image, the corresponding position on the table should be marked with any other character, e.g. “x”.

**3. Selecting and cropping confocal images**

The image analysis starts with separate stacks for each confocal channel and a metadata file with information such as voxel sizes. To create the stacks, use the Fiji macro *~/Rib_meristem_analysis/Fiji_macros/confocal_to_TIF.ijm* (select using Fiji>Plugins>Macros>Run). Follow the instructions to open the confocal image, specify the rootname for the images, crop the image and split the channels. The macro creates a folder within *~/Rib_meristem_analysis/processed_*images, named with the given rootname and containing TIF stacks for each of the channels selected: rootname_R.tif for FM4-64/PI channel, rootname_G.tif for GFP channel, if used. In addition, a Metadata.txt file is created, with information about the file name, path to original confocal stack and voxel sizes. The Metadata file will also be updated with the specific parameters used by each subsequent script used to analyse the images.

The folders with test images are provided with the output of c *confocal_to_TIF.*ijm, i.e. Metadata, _R.tif and _G.tif files.

**4. Landmarking**

Landmarks need to be added as reference points to select different regions (such as meristem and buds), to find the main axis of the apex etc. These landmark files need to be given specific names to be called by the Python scripts. For this, run the Fiji macro landmarks_3D.ijm (select using Fiji>Plugins>Macros>Run). Follow the instructions on the screen; typically the image to open is rootname_R.tif.

When processing images to measure cell wall orientations (see below), the main axis (e.g. of the stem) must be defined. For this, select 2 points must be selected in the order: top (e.g. inflorescence meristem summit), and bottom (e.g. center of stem near the bottom of the image). Save landmarks as rootname_axis.

When processing images to analyze Cre-loxP sectors, landmarks are placed at the boundaries of floral buds to find the main axis and to subsequently align and superimpose multiple images. For this, select a point at the center of the boundary between the meristem and a floral bud, for the 5 youngest buds. Save landmarks as rootname_boundaries.

For the sectors analysis, it is also necessary to manually landmark the cells in each sector using the Fiji macro *mark_GFP_sectors.ijm* (Fiji>Plugins>Macros>Run)*.* Follow the instructions on the terminal to open the image of cell outlines (rootname_R.tif) and the image with GFP signal (rootname_sectors_G.tif). Adjust its brightness of the GFP image on the B&C window, click on "Apply", say "Yes" to applying to the whole stack, then click on the "OK" button in the window "Please adjust brightness and contrast". You will see a Point Picker window with the images fused and a yellow square on the top left corner. Scroll up and down the image, choose a sector and click only once in each cell of the sector; you will see crosses appearing on the marked cells. Before moving to the next sector, click on the square in the corner - this is important to create a marker point to separate each sector. Save points as rootname_sectors (use the button with a page icon on the Fiji menu bar), then click on "Click OK when done".

**5. Segmentation and cell measurements**

Once the rootname_R.tif, rootname_G.tif (if applicable), Metadata.txt and landmarks (.points) files are in place, the images can be segmented and the cells measured. For segmentation, measurement of cell volumes and position relative to the main axis and to the apex, the scripts *watershed_segmentation.py, cell_data_table.py*  and *rib_zone.py* are called. Details of these scripts, input parameters and output are given in the instructions for 3D_meristem_analysis [1]. To run these scripts and the new cell wall scripts below, set the corresponding columns in the images table to "0" and double click on the shell script *~/Rib_meristem_analysis/shell scripts/Rib_meristem_analysis.*

To visualize segmented images, open them in Fiji and select the LUT “seg” on the pull down menu *Images>Lookup tables*. If the colors are not displayed correctly, it is necessary to re-set the LUT; for this, run the Fiji macro reset_LUT (select using Fiji>Plugins>Macros>Run).

**6. Finding new walls and their orientation**

After running *watershed_segmentation.py, cell_data_table.py*  and *rib_zone.py*, it is possible to identify newly deposited cell walls and their orientation by calling the scripts:

*new_walls.py*

This script detects newly deposited cell walls in images of tissue stained by mPS-PI [4], assuming that mPS-PI signal is proportional to wall thickness. A new wall is flagged when two neighboring cells share the same wall as their wall with lowest intensity. To correct for diminishing intensity with increasing depth in the confocal stack, the mPS-PI image in each plane is normalized using the average signal within the segmented cells. The normalized cell wall intensities are then corrected for the bias introduced by the fact that walls parallel to the imaging plane appear weaker; for this, a corrective function is obtained by plotting all wall intensities as a function of their angle to the imaging plane. The corrected cell wall intensities are added to the walls data table and an image of new walls is saved.

Inputs are the mPS-PI confocal stack (_R.tif) and the corresponding segmented image (_seg.tif). If available, an image of cell walls and the corresponding walls data table are read, otherwise they are created.

Default parameters are: cs = 20 (size of the image cropped around each cell wall during processing); wmin = 165 (minimum number of voxels in accepted cell walls); wmax = 2000 (maximum number of voxels in accepted cell walls). If needed, these parameters can be changed directly on the script with a standard script editor.

Outputs are the walls data csv table containing corrected cell wall intensities and with new walls marked, and a 16 bit TIF image stack of the new walls.

*cell_wall_orientation.py*

This script takes an image of newly deposited cell walls (produced by the script "new_walls.py") and calculates the orientation of each new wall in relation to the image main axis. "Angle to the main axis" is the angle between the given vector for the image main axis and the vector normal to plane best fitting the wall; "Angle to the radial axis" is the angle between the vector normal to best fitting plane and a vector perpendicular to the main axis that crosses the wall's center of mass.

Input files are the segmented image, corresponding image of cell walls, image or landmarks for the main axis, cell data table and cell walls table. The calculated angles are added to the walls data table (produced by the script "new_walls.py") and the cell data tables. Images are produced in which the new walls are given values proportional to the angles to the main axis or radial axis (heat map images"_new_wall_angles_main_axis.tif" and"_new_wall_angles_radial_axis.tif"). Images are also saved in which the two cells flanking each new wall are given values proportional to the angles to the main axis or radial axis (heat map images"_new_cell_angles_main_axis.tif" and"_new_cell_angles_radial_axis.tif").

Default parameters are: cs = 20 (size of the image cropped around each cell wall during processing) bar_min = 0 (minimum values for angle in heat map images) bar_max = 90 (maximum values for angle in heat map images). If necessary, these parameters can be changed directly on the script with a standard script editor.

To visualize the heat map images, open in Fiji and choose the LUT "walls_hm" on the pull-down menu *Image>Lookup Tables.* To visualize the image of segmented cell walls, open in Fiji and select the LUT “seg” on the pull down menu *Images>Lookup tables*; if the colors are not displayed correctly, run the Fiji macro reset_LUT (select in Fiji>Plugins>Macros>Run).

**7. Analysis of Cre-loxP sectors**

To detect Cre-loxP sectors and their 3D orientation, it is necessary first to landmark the organ boundaries, manually mark the cells in each sector, segment and measure the cells (see steps 4 and 5 above). After this is done, make sure that the columns for the scripts below are set to "0". Select only the images that you want to merge if different genotypes are compared, each group of images has to be overlapped and analyzed in a different run. After the images table is set up and saved, run the shell script *~/Rib_meristem_analysis/shell scripts/Clonal_analysis.* This will call the following scripts:

*cell_layers.py*

This script attributes cells to tissue layers. This is necessary because the centers of mass of epidermal cells are used to produce an outline of the superimposed apices with marked sectors. This script is described in detail in the instructions for 3D_meristem_analysis [1].

*sectors_merge.py*

This script will save in each rootname folder an image (rootname_sector_landmarks.tif), in which dots labeled with a unique number are placed in the coordinates of the landmarks found in the file rootname_sectors.txt. The centers of epidermal cells are also marked to produce an outline of the apex. A similar image is produced in which the same landmark dots are labelled with sector numbers. A table is produced (rootname_landmarks_data.csv), listing the landmarks, their sector numbers, coordinates, distance to the apex and main axis, and measurements of the corresponding cells listed in the table (rootname_cell_data.csv) produced by *cell_data_table.py*.

In addition, three new images and a table are placed in the path *~/Rib_meristem_analysis/processed_images/*. In the image Merged_landmarks.tif, each of the rootname_sector_landmarks.tif image was rotated to align the main axis of the stem with the central axis of the image (running vertically through the centre), shifted to place the meristem summit point on plane 10 and rotated around the main axis to align the bud landmarks found in the file rootname_boundaries.points (bud P0 is placed at position 12 o'clock and older buds are aligned clockwise). Merged_landmarks.tif is a similar overlap of all the aligned rootname_sector_landmarks.tif images. Merged_boundaries.tif is another overlap of aligned images, but showing the positions of the landmarks placed on the bud boundaries in each image (to check how well the alignment worked). The table Merged_sectors_data.csv lists all the landmarks in Merged_landmarks.tif, the rootname of the image from which they originated, their coordinates, and the corresponding data from each of the rootname_cell_data.csv tables.

Inputs are:

images_table.csv; segmented image (rootname_seg.tif); image of main axis of the stem (rootname_main_axis.tif, produced by script rib_zone.py); file with coordinates of landmarks placed on each cell in sectors (rootname_sectors.txt); file with coordinates of landmarks placed on bud boundaries (rootname_boundaries.points)

Default parameters are:

radius = 2 (size in micrometers of sphere used to label landmarks on image); cs = 20 (defines the size of the image cropped around each sector during processing); sm = 1000 (defines value to label summit point; has to be more than the total number of objects in the merged sectors image).

*sectors_analyse.py*

This script uses the coordinates of sector landmarks in the merged image produced by *sectors_merge.py* to calculate the main axis of each sector and its orientation. To calculate the vector of the main axis, the linalg.svd (single value decomposition) function of Numpy is used. To calculate the size of the main axis, a plane perpendicular to the vector is placed on the center of mass of the sector landmarks; the distance between each landmark and the plane is calculated, and the length of the main axis on each side of the plane equals the maximum landmark distance for that side. The orientation of the sector main axes is calculated as angle to the main axis (central z on the merged images) and to a vector perpendicular to the main axis and crossing the center of mass of the sector landmarks.

Images are produced of the sector landmarks, labeled with the corresponding sector number (Sector_landmarks.tif), of the corresponding sector axes (Sector_axes.tif) and of the sector axes overlapped with sector landmarks, to verify that the axes have been calculated correctly (Sector_axes_and_landmarks.tif). A table is also produced (Sectors_analysis.csv) with the coordinates for the center of mass, main axis extremities, main axis orientation and size.

In addition, the script produces the images "Sectors_landmarks_radial.png", Sectors_landmarks_top.png ", "Sector_3D_axes_radial.png", "Sector_3D_axes_top.png". These correspond to top projections (along the z axis) and radial projections of the landmark and axis images. To make the radial projection, each sector/axis was projected onto a plane containing its center of mass and the main image axis; sectors/axes on the right and left sides of the vertical projection are placed respectively on the right and left sides of the radial projection.

Finally, a lut table is saved, listing the RGB colors attributed to each sector/axis on the projections. This lut table can be used imported by Fiji (Plugins/LUT_editing/LUT importer) to visualize the landmarks and axes in the images Sector_landmarks.tif, Sector_axes.tif and Sector_axes_and_landmarks.tif.

Inputs are:

table "Merged_sectors_data.csv" (produced by sectors_merge.py); image "Merged_sectors.tif" (produced by sectors_merge.py); text file "Merged_images_metadata.txt" (produced by sectors_merge.py)

Default parameters are:

sm = 1000 (value attributed to summit point when aligning images; must match the value used in sectors_merge.py); radius = 2 (size in micrometers of sphere used to label landmarks on images); cs = 20 (defines the size of the image cropped around each sector during processing)

After running these scripts for one set of images, remember to save the files with a different name or move them to a different folder, or they will be overwritten by the subsequent run.

**References:**

1. Serrano-Mislata, A., Schiessl, K., and Sablowski, R. (2015). Active Control of Cell Size Generates Spatial Detail during Plant Organogenesis. Current Biology *25*, 2991-2996.

2. Schindelin, J., Arganda-Carreras, I., Frise, E., Kaynig, V., Longair, M., Pietzsch, T., Preibisch, S., Rueden, C., Saalfeld, S., and Schmid, B. (2012). Fiji: an open-source platform for biological-image analysis. Nature Methods *9*, 676-682.

3. Schmid, B., Schindelin, J., Cardona, A., Longair, M., and Heisenberg, M. (2010). A high-level 3D visualization API for Java and ImageJ. BMC bioinformatics *11*, 274.

4. Truernit, E., and Haseloff, J. (2008). A simple way to identify non-viable cells within living plant tissue using confocal microscopy. Plant Methods *4*, 15.
