# Supplementary figures and images for: Control of Oriented Tissue Growth through Repression of Organ Boundary Genes Promotes Stem Morphogenesis
Source: Dev Cell. 2016 Oct 24;39(2):198–208. doi: 10.1016/j.devcel.2016.08.013 (PMC5084710; doi:10.1016/j.devcel.2016.08.013)

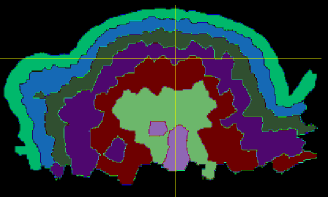

Supplement: Data S1. Annotated Source Code and Instructions for Installation and Use of Scripts for Image Analysis, Related to Experimental Procedures [file mmc6.zip › Rib_zone_analysis/Instructions/icon.png]
