# Supplementary material for: Control of Oriented Tissue Growth through Repression of Organ Boundary Genes Promotes Stem Morphogenesis
Source: Dev Cell. 2016 Oct 24;39(2):198–208. doi: 10.1016/j.devcel.2016.08.013 (PMC5084710; doi:10.1016/j.devcel.2016.08.013)
Supplement: Data S2. Annotated Source Code, Instructions for Installation and Use of Scripts for ChIP-Seq Analysis, and Gene Annotations Used, Related to Experimental Procedures [file mmc7.zip › Peaks_analysis/Instructions/Peaks_analysis_instructions.docx]

Bencivenga et al.

**Supplementary Software 2 - Filtering and analyzing ChIPseq peaks**

The protocol below was used after calling ChIP-seq peaks with MACS2 as described in the Experimental Procedures of Bencivenga et al (2016) to filter ChIP-seq peaks for reproducibility across replicates, to associate peaks to gene models, to analyze the distribution of peaks within associated genes and to select input sequences for MEME-ChIP.

**1. Installation**

To use the scripts, expand the folder *Peaks_analysis* and place it on the Desktop. The scripts were written in Python 2.7.3 on an Apple computer running MacOS X 10.9.4 - changes may be needed to install and run them on a different platform. Dependencies are Numerical Python (http://www.numpy.org), matplotlib (http://matplotlib.org) and Tcl/tk (https://www.python.org/download/mac/tcltk/).

**2. Filtering peaks for reproducibility across replicates and attributing gene models**

ChIP-seq reads from three replicate treatments and three replicate controls were aligned against the genome and peaks called as described in Experimental Procedures). To calculate fold enrichments and *q*-values, the combined replicates were compared with the combined controls using MACS 2.0.10 [59]; this generated the files RPL-GFP_fused_peaks.narrowPeak and wt_fused_peaks.narrowPeak.gz. In addition, MACS 2.0.10 was applied to individual replicates to select for consistency across replicates; this generated files RPL-GFP1_peaks.narrowPeak, RPL-GFP2_peaks.narrowPeak, RPL-GFP3_peaks.narrowPeak, wt1_peaks.narrowPeak, wt2_peaks.narrowPeak, wt3_peaks.narrowPeak. These files are deposited at NCBI (http://www.ncbi.nlm.nih.gov/geo/query/acc.cgi?&acc=GSE78727) and need to be placed together in a local folder for the analysis below.

To filter for consistency across replicates, the script Overlap_MACS2_files.py is called by double-clicking on the shell script ~/Peaks_analysis/shell_scripts/Overlap_MACS2_files.sh. The script searches for overlaps between peak regions listed in selected replicates (GFP1_peaks.narrowPeak, RPL-GFP2_peaks.narrowPeak, RPL-GFP3_peaks.narrowPeak files), and accepts the overlaps if they are absent from all controls, have q-values of a specified value or lower in each replicate, and the overlapping region is at least of a specified length.

After the filtering step above, peaks are attributed to gene models and an annotated table is produced. For this, tables with gene coordinates and with gene annotations are selected - see ~/Peaks_analysis/TAIR10_tables/TAIR10_AGI_location.txt and TAIR10_functional_descriptions.txt. Based on these tables, peaks are associated with gene models within specified distances upstream and downstream of the transcribed regions, without intervening genes.

Inputs are selected interactively:

path to folder containing narrowPeak files

path to table containing gene positions

path to table containing gene annotations

Parameters are set directly on the script (using a suitable editor such a IDLE):

n is the minimum width in nucleotides for accepting the overlap between MACS peaks (default n = 50)

prom is the length in nucleotides for the upstream regulatory region for each gene model (used to associate peaks with genes); default prom = 4000

utr is the length in nucleotides for the downstream regulatory region for each gene model (used to associate peaks with genes) default utr = 1500

chromosomes specifies chromosome names and lengths in nucleotides

default chromosomes = np.array([['Chr1', 30500000], ['Chr2', 19700000], ['Chr3', 23500000], ['Chr4', 18600000], ['Chr5', 27000000]])

fc_cutoff sets the position of bedgraph line for fold change; default fc_cutoff = 3

q_cutoff = 3 sets cutoff q value for overlapping peaks from different replicates; default q_cutoff = 3

Outputs are:

A tab-delimited text table with the MACS2 statistics, gene models and annotation associated with each overlapping peak region (named Overlapping_peaks_AGI.txt, saved in the same folder as the narrowPeak files).

A tab-delimited text table with the nucleotide position of the center of each overlapping peak region (named "Peak_positions.txt" and saved in the same folder as the narrowPeak files).

bedgraph files (which can be opened in the IGV browser) for peak fold changes (Overlapping_peaks_fold_change.bedgraph) and for peak q-values (Overlapping_peaks_q_values.bedgraph), both placed in the same directory as the narrowPeak files.

**3. Analyzing the distribution of peaks within associated genes**

To statistically analyze peak locations within genes, the script peak_statistics.py is called by double-clicking on the shell script ~/Peaks_analysis/shell_scripts/Peak_statistics.sh. This script interactively selects the file with the position of overlapping peak regions and the file with associated gene models (both produced in step 2 above), the table with gene coordinates (~/Peaks_analysis/TAIR10_tables/TAIR10_AGI_location.txt), and asks for the number of replicates used when scoring the positions of simulated, random peaks.

The script scores the frequency of observed peak regions centered on the transcribed, upstream and downstream regions, then uses a Monte Carlo method to estimate the p-value for the hypothesis that these frequencies correspond to a random distribution of peaks within genes. A tab-delimited text table with the results is produced, in addition to histograms showing the frequency of observed and simulated (random) peaks at different distances to the start and end of transcribed regions (Peak_position_statistics.txt, Peaks_histogram_upstream.png, Peaks_histogram_downstream.png, all saved in the same folder as the selected file with peak positions).

Set parameters chromosomes, which specify chromosome names and lengths in nucleotides; default values are:

chromosomes = np.array([['Chr1', 30500000], ['Chr2', 19700000], ['Chr3', 23500000], ['Chr4', 18600000], ['Chr5', 27000000]])

**4. Selecting input sequences for MEME-ChIP**

To detect enrichment for sequence motifs within overlapping peak regions, MEME-ChIP (http://meme-suite.org/tools/meme-chip) was used in discriminative mode, comparing the sequences around observed peaks with a control set of sequences around a ten-fold larger number of random peaks (Experimental Procedures). To produce both sets of sequences, the script peak_sequences.py is called by double-clicking on the shell script ~/Peaks_analysis/shell_scripts/Peak_sequences.sh.

The script interactively selects the file with the positions of overlapping peak regions and the file with associated gene models (both produced in step 2 above), the table with gene coordinates (~/Peaks_analysis/TAIR10_tables/TAIR10_AGI_location.txt), then asks for the size of the region to include on each side of the center of each peak region. A folder is also selected with chromosome sequences. This is not included here and must be downloaded from public databases (e.g. TAIR, http://www.arabidopsis.org) - one file for each chromosome, with a single sequence in FASTA format; the files must be named "TAIR10_chr1.fas", "TAIR10_chr2.fas" etc.

To produce a control set of sequences for discriminative MEME, the script generates random peak positions in each chromosome. The size of the control set (how many times larger than the observed set) is set interactively.

The outputs are a list of sequences flanking the centers of observed peak regions (Peak_sequences.txt) and a list of sequences flanking random peak centers (Random_sequences.txt), both in FASTA format, saved in the same folder containing the input file with the positions of peak regions.

Set parameters chromosomes, which specify chromosome names and lengths in nucleotides; default values are:

chromosomes = np.array([['Chr1', 30500000], ['Chr2', 19700000], ['Chr3', 23500000], ['Chr4', 18600000], ['Chr5', 27000000]])
