# Supplementary material for: Control of Oriented Tissue Growth through Repression of Organ Boundary Genes Promotes Stem Morphogenesis
Source: Dev Cell. 2016 Oct 24;39(2):198–208. doi: 10.1016/j.devcel.2016.08.013 (PMC5084710; doi:10.1016/j.devcel.2016.08.013)
Supplement: Document S2. Article plus Supplemental Information [file mmc8.pdf]

Article

Developmental Cell

Control of Oriented Tissue Growth through Repression of Organ Boundary Genes Promotes Stem Morphogenesis

Graphical Abstract

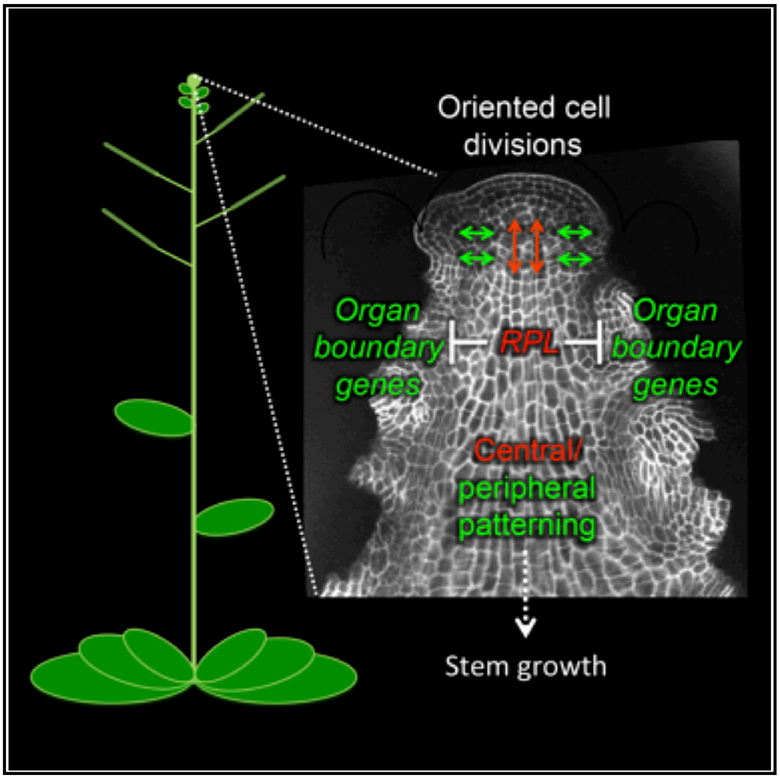

Authors

Stefano Bencivenga, Antonio Serrano-Mislata, Max Bush, Samantha Fox, Robert Sablowski

Correspondence

robert.sablowski@jic.ac.uk

In Brief

The origin of the stem is a major gap in our understanding of plant development. Bencivenga et al. show that during stem initiation in *Arabidopsis*, the regulatory gene *REPLUMLESS* controls three-dimensional patterns of cell division and growth through repression of organ boundary genes.

Highlights

- Image and sector analysis revealed 3D growth patterns in early stem development
- *Arabidopsis RPL* controls oriented cell division and growth in the rib meristem
- RPL interacts with many of the key genes that regulate shoot organogenesis
- *RPL* controls oriented growth by directly repressing organ boundary genes

Accession Numbers

GSE78727
GSE78511

Bencivenga et al., 2016, Developmental Cell *39*, 198–208
October 24, 2016 © 2016 The Author(s). Published by Elsevier Inc.
http://dx.doi.org/10.1016/j.devcel.2016.08.013

CellPress

Developmental Cell
Article

Control of Oriented Tissue Growth through Repression of Organ Boundary Genes Promotes Stem Morphogenesis

Stefano Bencivenga,[1] Antonio Serrano-Mislata,[1] Max Bush,[1] Samantha Fox,[1] and Robert Sablowski[1,2,*]
[1]Cell and Developmental Biology Department, John Innes Centre, Norwich Research Park, Norwich NR4 7UH, UK
[2]Lead Contact
*Correspondence: robert.sablowski@jic.ac.uk
http://dx.doi.org/10.1016/j.devcel.2016.08.013

SUMMARY

The origin of the stem is a major but poorly understood aspect of plant development, partly because the stem initiates in a relatively inaccessible region of the shoot apical meristem called the rib zone (RZ). We developed quantitative 3D image analysis and clonal analysis tools, which revealed that the *Arabidopsis* homeodomain protein REPLUMLESS (RPL) establishes distinct patterns of oriented cell division and growth in the central and peripheral regions of the RZ. A genome-wide screen for target genes connected RPL directly to many of the key shoot development pathways, including the development of organ boundaries; accordingly, mutation of the organ boundary gene *LIGHT-SENSITIVE HYPOCOTYL 4* restored RZ function and stem growth in the *rpl* mutant. Our work opens the way to study a developmental process of importance to crop improvement and highlights how apparently simple changes in 3D organ growth can reflect more complex internal changes in oriented cell activities.

INTRODUCTION

Virtually all plant growth is sustained by stem cell populations located within the apical meristems (Aichinger et al., 2012). Decades of intense study have revealed much about how the meristems form roots, leaves, and floral buds. In contrast, little is known about how the stem is initiated in the subapical region of the shoot meristem and how regulatory genes that function in this region influence stem size and shape. The origin of the stem is not only a major aspect of plant development that has been relatively neglected, but is also of great importance in crop improvement: genes that modify stem development have played a key role in yield increases in the last 50 years (Khush, 2001), but the developmental basis for their effects on plant architecture remains unclear.

The shoot apical meristem, which produces leaves, flowers, and the stem, has distinct zones with different functions (Fletcher, 2002) (Figure 1F). Leaves and floral buds are initiated in the peripheral zone (PZ), while long-term progenitors in the central zone (CZ) constantly replenish the PZ. The underlying rib zone (RZ) gives rise to the stem and includes a central region called the rib meristem (named after its distinct pattern of transversal cell divisions), which gives rise to the pith, and a peripheral region that appears continuous with the overlying PZ and gives rise to the epidermis, cortex, and vascular tissues of the stem (Sachs, 1965; Sanchez et al., 2012). Superimposed on this functional zonation, the shoot meristem has a layered structure; in angiosperms such as *Arabidopsis*, the cells in the outermost two to three layers divide mostly anticlinally (perpendicular to the meristem surface), so their descendants generally remain in the same layer (Fletcher, 2002). Growth of the different meristem regions can be controlled differentially: during the vegetative stage in *Arabidopsis*, the CZ and PZ sustain leaf production but the RZ is inhibited, whereas at the transition to flowering, activation of the RZ leads to rapid stem elongation while the CZ and PZ start to produce floral buds.

Parallels can be drawn between activation of stem growth at the shoot apex and the well-studied control of root growth at the opposite end of the plant's main axis (Aichinger et al., 2012), but there are important differences. In the root, terminal growth mostly precedes the emergence of lateral roots and the vast majority of cell growth and division is aligned with the main root axis, so growth rate is proportional to root meristem size and to the overall rate of cell proliferation (Beemster and Baskin, 1998). In contrast, development of the stem occurs simultaneously with that of lateral structures such as flower buds, and cell files in the RZ appear much less organized than in the root. The more complex structure of the RZ requires three-dimensional (3D) analysis of cell behavior and overall organ growth. A further complication is the relative inaccessibility of the RZ in comparison with the root meristem. Thus RZ growth and early stem development remain considerably less well understood than the root system, and illustrate the general challenge of describing and understanding the regulation of tissue growth in 3D structures with no obvious internal landmarks.

In *Arabidopsis*, one of the master regulators of stem growth is most often named *PENNYWISE* (*PNY*) (Smith and Hake, 2003), *REPLUMLESS* (*RPL*) (Roeder et al., 2003), and *BELLRINGER* (*BLR*) (Byrne et al., 2003); we used *rpl* mutant alleles and therefore adopted *RPL* for simplicity. *RPL* encodes a BEL1-like TALE homeodomain (BLH) transcription factor that controls multiple aspects of meristem and floral development, including meristem maintenance, the distribution of lateral organs around the meristem (phyllotaxis), the transition to flowering and the

 © 2016 The Author(s). Published by Elsevier Inc.
This is an open access article under the CC BY license (http://creativecommons.org/licenses/by/4.0/).

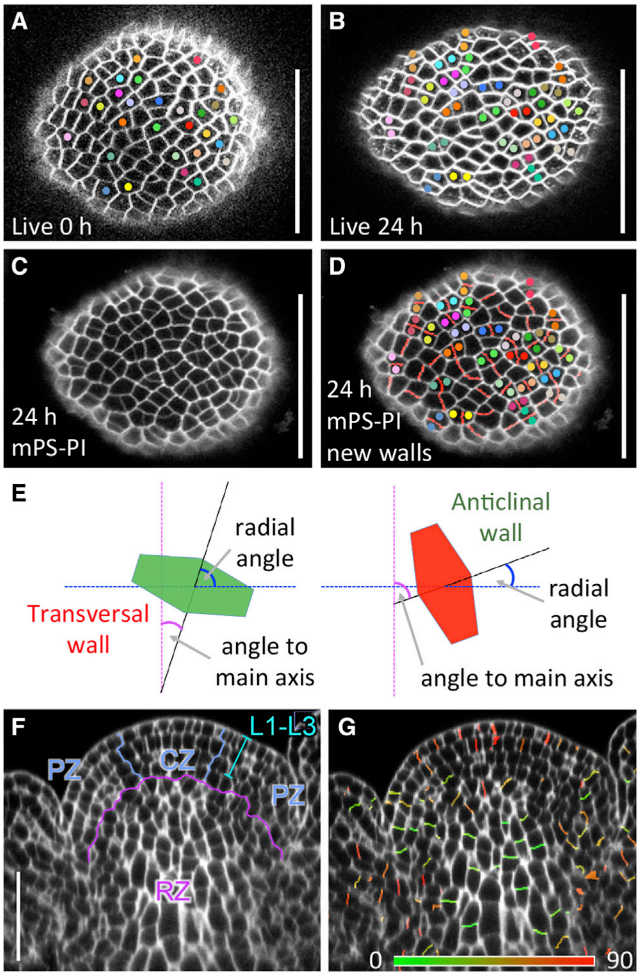

Figure 1. Automated Detection of the 3D Orientation of Cell Divisions in the Shoot Apex

(A and B) Confocal sections through the outer layers of a live *Arabidopsis* (L-*er*) inflorescence meristem stained with FM4-64 at 0 hr (A) and 24 hr later (B). When cell divisions occurred, mother cells in (A) and their corresponding daughter cells in (B) were manually marked with dots of the same color.

(C) Confocal section of the same meristem as in (B), after staining by modified pseudo-Schiff propidium iodide (mPS-PI), showing variable intensity of cell-wall signals.

(D) Same section as in (C), overlaid with an image of segmented cell facets detected as new walls (red lines); colored dots mark recent divisions corresponding to (B). Note that new walls were correctly attributed for most of the recent divisions (27 out of 29), in addition to low-intensity walls that likely correspond to divisions completed more than 24 hr earlier.

(E) Scheme of how the orientation of new walls was measured. Walls are represented by colored hexagons, with a line normal to their best-fitting plane shown in black; the magenta line is parallel to the main axis of the stem; the blue line is perpendicular to the to the main axis of the stem and passes through the wall's center of mass; angles to the main axis and radial angles are indicated by the magenta and blue arcs, respectively; the red and green walls correspond to transversal and anticlinal walls, as frequently seen in the RZ and in the outer meristem layers, respectively; anticlinal walls can have small to large radial angles, depending on whether they face the central axis or not.

(F and G) Longitudinal slice through a stack of confocal images of mPS-PI-stained inflorescence apex, with (F) the outer meristem layers (L1–L3), central zone (CZ), peripheral zone (PZ), and rib zone (RZ) indicated. (G) Image corresponding to (F), overlaid with an image of cell facets detected as new walls and colored according to the angle to the main axis of the stem.

Scale bars, 50 μm. See also Figure S1.

associated activation of stem development, and subsequently floral organ patterning (Byrne et al., 2003; Roeder et al., 2003; Smith and Hake, 2003; Arnaud et al., 2011). Based on its expression in the shoot meristem, extending into the RZ (Smith and Hake, 2003; Andrés et al., 2015), *RPL* likely affects stem growth by regulating the earliest steps in stem development, but the molecular and cellular processes controlled by *RPL* in the RZ are virtually unknown.

Here, we used quantitative 3D imaging and clonal analysis to reveal how *RPL* controls early stem development. Our findings indicate that *RPL* controls RZ function through oriented cell activities rather than local rates of cell proliferation. We also show that RPL directly interacts with many of the key regulatory genes in shoot organogenesis and that interaction with genes involved in organ boundary development are particularly important for the role of *RPL* in the RZ.

RESULTS

RPL Is Required for Oriented Tissue Growth in the RZ

If *RPL* controls morphogenesis in the RZ, it would be expected to modify rates or orientations of tissue growth, or a combination of both. To verify this we would require new imaging and analysis methods, because tracking cells by live imaging (Serrano-Mislata et al., 2015) is not feasible in the deeper layers of the shoot meristem, whereas high-resolution 3D images of fixed apices (Schiessl et al., 2012) cannot provide temporal information. Instead, we exploited the fact that new cell walls are placed perpendicular to the mitotic spindle (Smith, 2001), thus retaining information about the orientation of recent cell divisions. To detect recent cell divisions, we cross-linked wall polysaccharides to propidium iodide (PI) (Truernit et al., 2008), which would be expected to produce lower fluorescence for thinner, more recently synthesized walls. After 3D segmentation the PI signal was measured in all facets between cells, and individual facets were identified as new walls if they had the weakest signal density for both adjacent cells (details in Supplemental Experimental Procedures and annotated source code in Data S1). This method accurately detected cell divisions identified by time-lapse imaging in both outer and inner meristem layers, and correctly detected the predominance of anticlinal divisions in the outer meristem layers and of transversal divisions in the RZ (Figures 1 and S1). Thus information about 3D patterns of oriented cell divisions can be extracted from single-time-point images of fixed shoot apices.

We next used the method to compare shoot apices of wild-type and *rpl-1* mutant plants. The RZ of the wild-type apex showed a well-defined rib meristem with cell divisions perpendicular to the main stem axis, while the peripheral region was enriched for radial cell divisions, which potentially increase RZ width and may contribute to elongating the basal region of floral pedicels (Figure 2A). In contrast, *rpl-1* appeared to have a less organized RZ and the difference between the central and peripheral regions was less obvious (Figure 2B).

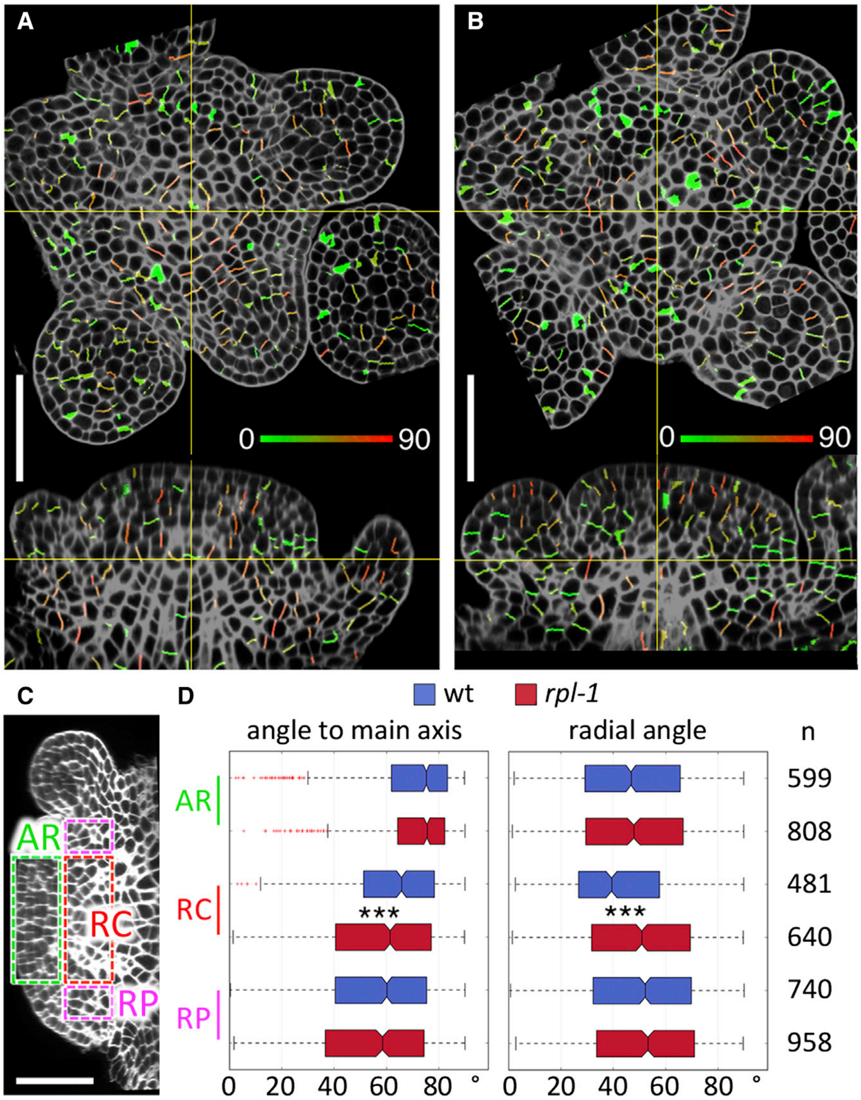

Figure 2. *rpl-1* Disrupts the Orientation of Cell Divisions in the RZ

(A and B) Orthogonal views of confocal image stacks of wild-type (wt) (L-*er*) (A) and *rpl-1* (B) inflorescence apices stained by mPS-PI, overlaid with images of segmented cell facets detected as new walls, and colored according to their radial orientation (see Figure 1E). In each image, the yellow cross-hairs mark the same point in the top and side views; note the less organized RZ in *rpl-1* and the high frequency of radially oriented divisions in the periphery of the wild-type RZ, but not in the mutant.

(C and D) Measurement of oriented divisions in wild-type and *rpl-1*. (C) Regions where new wall angles were measured (AR, apical region; RC, rib meristem core; RP, rib meristem periphery). (D) Boxplots show the distribution of new wall angles to the main stem axis or radial angles (see Figure 1E). n indicates the number of new walls in each set (combined data from four apices for each genotype); asterisks indicate statistically significant differences (***$p < 0.001$, Mann-Whitney test). In the boxplots, the box extends from the lower to upper quartile values with a line at the median; whiskers extend to 1.5 times the interquartile range, and outlier points beyond the whiskers are shown in red. Scale bars, 50 μm. See also Figure S2.

To quantify the differences between wild-type and *rpl-1*, we compared the orientation of recent cell walls in the region where the rib meristem originates (“RM core” [RC], within 30–60 μm of the meristem summit and 0–40 μm of the main axis), in the overlying CZ and PZ cells (“apical region” [AR], within 30 μm of the meristem summit and within 40 μm of the shoot main axis), and in the PZ surrounding the RM (“RM periphery” [RP], within 30–60 μm of the summit and 40–50 μm of the main axis) (Figures 2C and 2D). Significant differences were detected in the RC, where *rpl-1* showed a pattern more similar to that in the RP, with more variable angles to the main axis and more radially oriented divisions; these differences were seen not only in data from combined apices but also across individual apices (Figure S2).

The orientation of cell divisions can respond to cell geometry, which reflects principal directions of growth, or to the direction of mechanical stress, which can accumulate during growth of interconnected cells, and these physical signals can also be overruled by chemical signals (Kwiatkowska, 2004; Besson and Dumais, 2011; Yoshida et al., 2014). To test whether oriented divisions corresponded to directions of tissue growth and to obtain information about growth rates, we used a Cre-*lox*P recombination system (Gallois et al., 2004) to mark individual cells with GFP expression and track their descendants in the shoot apex (Figure 3A). To overlap marked sectors from multiple apices and analyze them in 3D, we landmarked cells within each sector manually and used custom scripts to align the images and measure the position, size, and orientation of the main axis for each sector (details in Supplemental Experimental Procedures and annotated source code in Data S1). As expected from the anticlinal cell divisions in the outer layers of the meristem, sectors in these layers were oriented tangentially to the meristem surface (Figures 3B and 3C). The wild-type sectors also confirmed the expectation that the RP originates from the overlying PZ of the meristem, where lateral organs are also initiated. Sectors in the RC grew vertically and more slowly than in the surrounding region, and based on their orientation appeared to originate from a region below the CZ progenitors that sustain the initiation of lateral organs (Aichinger et al., 2012) (Figure 3C). Based on cell number and length of sectors, growth rates were not significantly different between wild-type and *rpl-1*; in contrast, the orientation of *rpl-1* sectors was different from the wild-type specifically within the RC, and as seen in the analysis of recent cell walls, was more similar to the pattern seen in the RP region (Figures 3E–3H).

Based on the combined analyses of new cell walls and marked clones, we conclude that *RPL* regulates oriented tissue growth

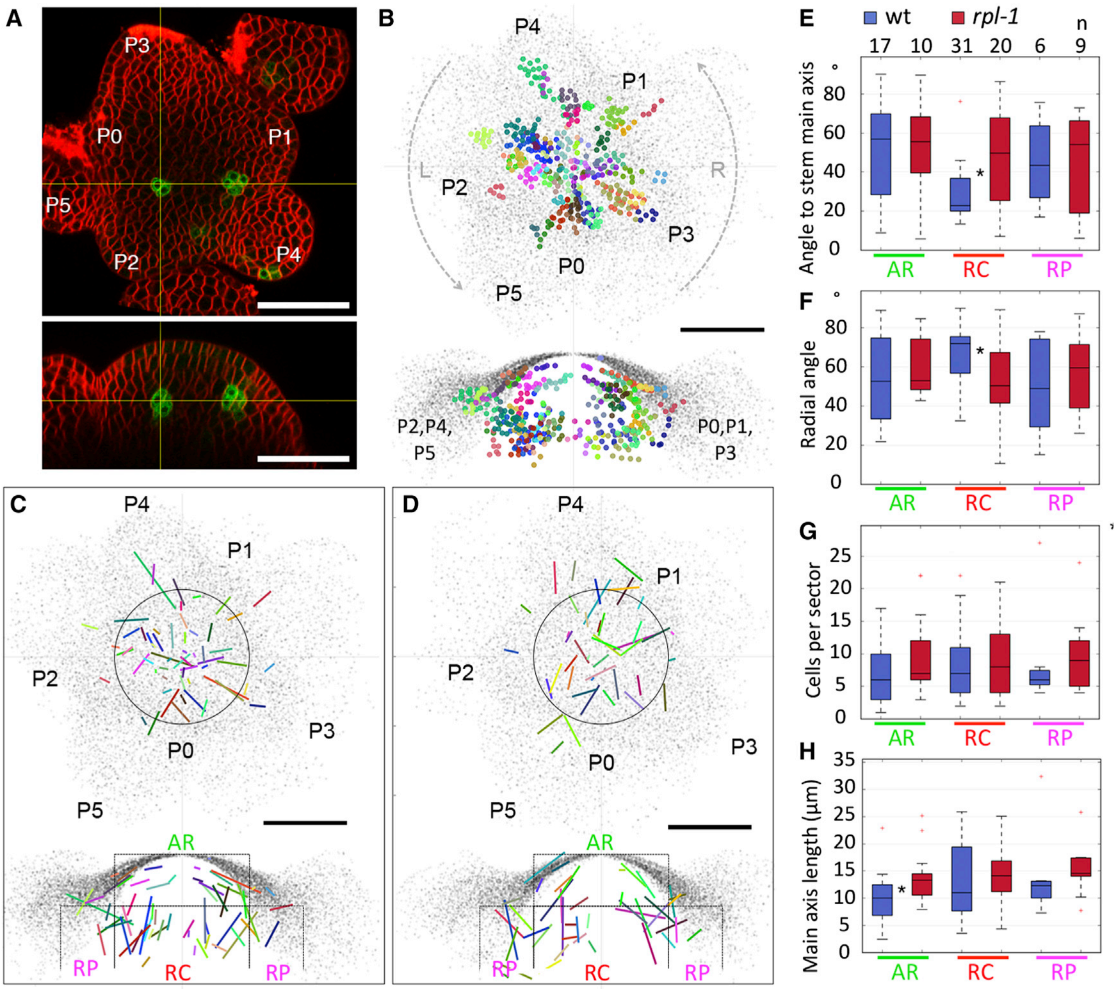

Figure 3. Clonal Analysis Shows that RPL Controls Orientation, but Not Rates of Growth, in the Central Region of the RZ

(A) Orthogonal views of a confocal images stack of a wild-type inflorescence apex with two mGFP5-ER-marked clones (green), 3 days after Cre-catalyzed recombination. P1 to P5 mark the positions of successive floral bud primordia.

(B) Vertical (top) and radial (bottom) projections of superimposed GFP-marked clones from 15 wild-type apices. Each clone is marked in a different color, with the position of individual cells indicated by dots. Each clone was projected onto a plane containing its center of mass and the stem main axis to produce the radial projection. Clones on the right and left sides of the vertical projection (arrows marked R and L) are shown, respectively, on the right and left sides of the radial projection.

(C) Vertical (top) and radial (bottom) projections of the main axes of clones shown in (B). On the radial projection, the AR, RC, and RP regions are defined as in Figure 2C.

(D) Projections of the main axes of clones from 15 superimposed *rpl-1* apices, produced as described for the wild-type clones in (B) and (C).

(E–H) Boxplots showing the vertical angles of the main axes of clones (relative to the stem main axis) (E), radial angles (relative the shortest line between the clone's center of mass and the stem main axis) (F), number of cells per clone (G), and length of the clone main axes (H). AR, RC, and RP correspond to the regions shown in (C) and (D); n indicates the number of sectors in each region; asterisks indicate statistically significant differences (*$p < 0.01$, Mann-Whitney test).

In the boxplots, the box extends from the lower to upper quartile values with a line at the median; whiskers extend to 1.5 times the interquartile range, and outlier points beyond the whiskers are shown in red. Scale bars, 50 μm.

and establishes distinct growth patterns in the central and peripheral regions of the RZ.

RPL Directly Binds to Key Genes that Regulate Meristem Function, Organ Patterning, and Growth

As a transcriptional regulator, *RPL* is expected to affect patterns of growth indirectly through its downstream target genes. To reveal genes and processes regulated by *RPL* in the RZ, we first used chromatin immunoprecipitation (ChIP) sequencing (ChIP-seq) to detect loci bound by RPL within the inflorescence apex. As internal controls, we used genes previously reported to interact with *RPL* genetically or by ChIP, including close partners or repressors of *RPL* function, such as *BREVIPEDICELLUS* (*BP*), *POUND-FOOLISH* (*PNF*), *ARABIDOPSIS THALIANA HOMEOBOX GENE 1* (*ATH1*), *KNOTTED1-LIKE HOMEOBOX GENE 6* (*KNAT6*), *BLADE ON PETIOLE 1* (*BOP1*), and *BOP2* (Smith and Hake, 2003; Smith et al., 2004; Khan et al., 2012, 2015; Ragni et al., 2008; Khan

CellPress

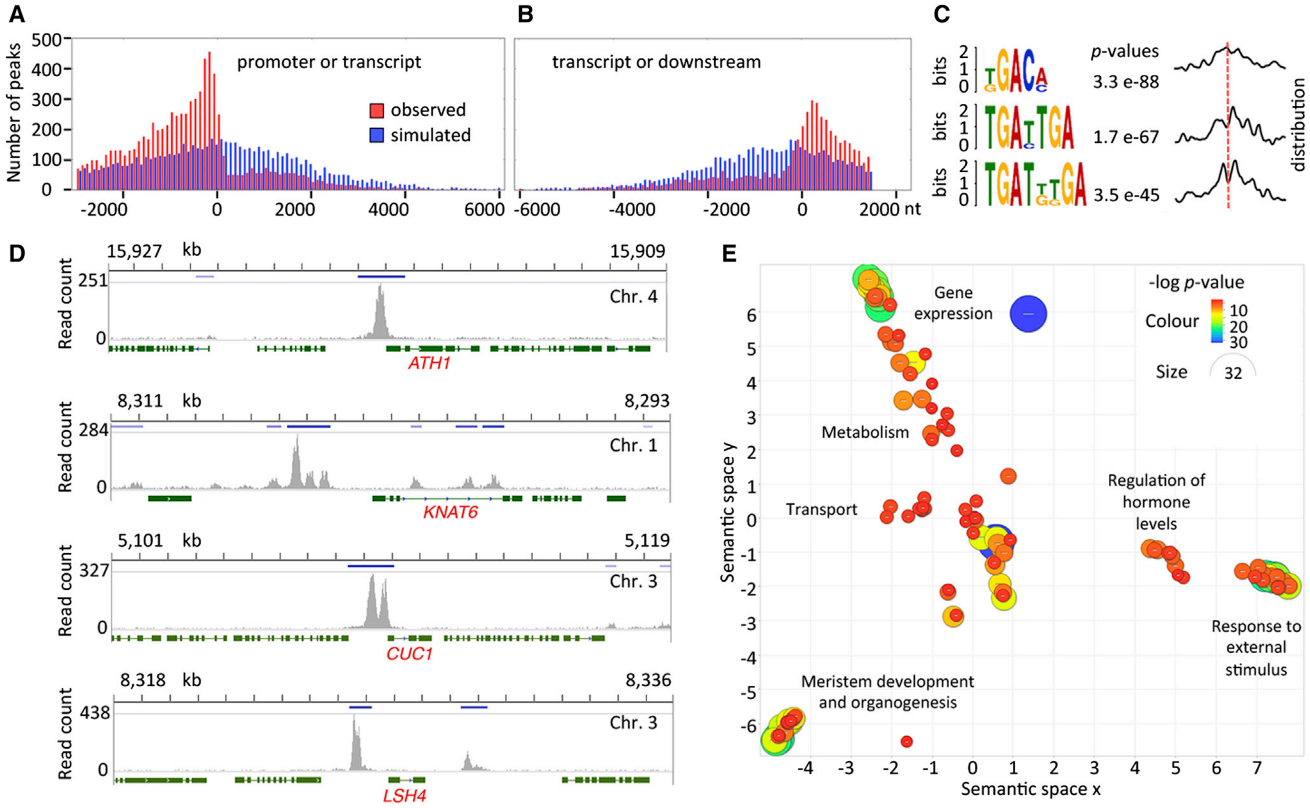

Figure 4. Genome-wide RPL Targets Identified by ChIP-Seq

(A and B) Enrichment of RPL binding sites within promoter regions (A) and downstream regions (B) compared with transcribed regions, for the 2,917 high-confidence RPL candidate targets (Table S1). Red bars show the frequency of peak regions (overlap of peaks from three ChIP-seq replicates) centered at the indicated nucleotide positions relative to transcript start (A) or end (B); blue bars show how simulated peaks at random genome positions were distributed within the set of high-confidence RPL targets. Based on 10,000 simulations, the observed RPL binding sites were significantly enriched ($p < 10^{-4}$) in both promoter and downstream regions compared with transcribed regions.

(C) Enrichment for sequence motifs within 75 nt of the RPL binding sites analyzed in (A) and (B), detected using the MEME Suite (Bailey et al., 2009). Curves on the right show the frequency distribution of enriched motifs, relative to the center of the peak region (red line).

(D) Representative raw ChIP-seq peaks (replicate 1 only) for control genes (*ATH1*, *KNAT6*) and two key organ boundary genes (*CUC1*, *LSH4*). Dark-blue bars show regions of overlap between peaks detected in three replicates (peak regions mentioned in A–C); green bars and lines are exons and introns, respectively; numbers above each graph show chromosome position in kilobases. All genes are oriented 5′ (left) to 3′ (right).

(E) Semantic clustering (Supek et al., 2011) of GO categories enriched in the set of 2,917 candidate RPL targets (Table S2). The diameter and color of each circle reflect the p value for individual GO terms within the cluster; the broad terms used to describe each cluster are attributed to specific GO terms in Table S2.

See also Figure S3; Tables S1 and S2.

et al., 2015), as well as genes that interact with *RPL* during flowering, floral organ, and fruit development, such as *LEAFY* (*LFY*), *AGAMOUS* (*AG*), *APETALA 1* (*AP1*), *SEPALLATA 3* (*SEP3*), *APETALA 2* (*AP2*), *SHATTERPROOF 1* (*SHP1*), *SHP2*, *FRUITFULL* (*FUL*), and *MIR156A*, *C*, and *E* (Lal et al., 2011; Roeder et al., 2003; Smaczniak et al., 2012; Andrés et al., 2015). Anti-GFP antibodies were used to pull down DNA bound by RPL-GFP expressed as a genomic fusion (*pRPL:RPL-GFP*) that mirrored the endogenous *RPL* expression and complemented the *rpl-1* mutant (Figure S1). ChIP-seq peaks with a false discovery rate of less than 0.001 and consistently detected in three RPL-GFP replicates but not in wild-type replicates were selected and associated with genes that contained a peak within 3 kb upstream and 1.5 kb downstream of their coding sequences (see examples in Figure 4D). From the list of genes that satisfied these conditions, we selected a set of 2,917 high-confidence candidates (Table S1) that showed a peak enrichment at least as high as the positive control gene with the lowest enrichment (*APETALA2*).

Within the high-confidence targets set, ChIP-seq peaks were depleted in transcribed regions but enriched in the immediately adjacent regions, as expected for the role of RPL as a transcriptional regulator (Figures 4A, 4B, and 4D). BLH proteins function with a KNOX homeodomain partner (Bellaoui et al., 2001), which is BP in the case of RPL (Smith and Hake, 2003). Accordingly, sequences in the vicinity of the peak summits were significantly enriched for short motifs containing TGAC/T (Figure 4C), similar to the binding sites previously described for BLH and KNOX proteins (Smith et al., 2002). Gene ontology (GO) analysis (Falcon and Gentleman, 2007) followed by semantic clustering (Supek et al., 2011) revealed clusters of highly enriched functional categories (Figure 4E and Table S2). As for other master regulatory genes (Kaufmann et al., 2009; Schiessl et al., 2014), the most highly enriched

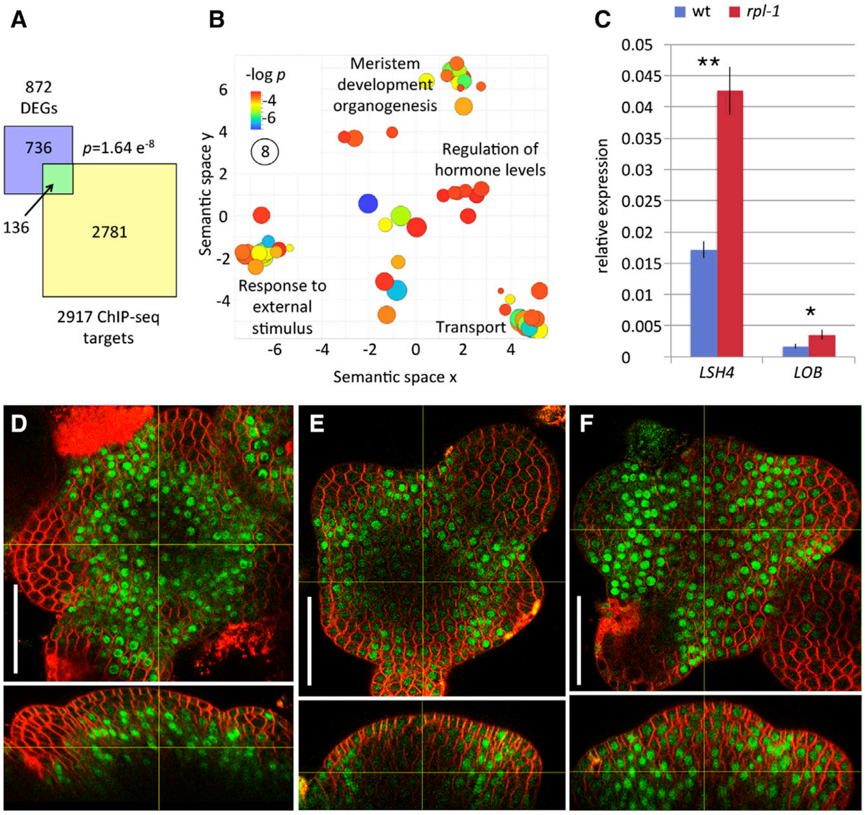

Figure 5. Genes Regulated by RPL Include the Boundary Gene *LSH4*

(A) Overlap between ChIP-seq results and genes that were differentially expressed (DEGs) in wild-type and *rpl-1* inflorescence apices (Table S3). The overlap of both sets was larger than expected by chance ($p = 1.64 \times 10^{-8}$, Fisher's exact test).

(B) Semantic clustering (Supek et al., 2011) of GO categories enriched in the set of 136 direct RPL target genes. The diameter and color of each circle reflect the p value for individual GO terms within the cluster; broad terms used to describe each cluster are attributed to specific GO terms in Table S4.

(C) qRT-PCR measurement of *LSH4* and *LOB* expression levels in wild-type and *rpl-1* inflorescence apices. Bars and lines show means and SD (three biological replicates), with asterisks indicating significant difference (Student's t test; *LSH4*: $^{**}p < 0.01$, *LOB*: $^{*}p < 0.05$).

(D–F) orthogonal views of confocal image stacks of inflorescence apices showing the expression pattern of *pRPL-RPL-GFP* (D), *pLSH4:LSH4-GFP* in wild-type (E), and *pLSH4:LSH4-GFP* in *rpl-1* (F). In each image, the yellow cross-hairs mark the same point in the top and side views. Scale bars, 50 μm.

See also Tables S3 and S4.

terms were related to transcriptional control (Table S1). The second most highly enriched cluster of GO terms corresponded to meristem functions, early organogenesis, and reproductive development, as detailed below. Additional sets of enriched terms were related to hormone metabolism and responses (particularly involving auxin, gibberellin, and jasmonic acid), ion and sugar transport, and responses to external stimuli (e.g., pathogens and light) (Figure 4E).

Genes in the ''meristem development and organogenesis'' cluster revealed direct links to many well-known players in shoot development. Reflecting the role of *RPL* in meristem establishment, its targets included genes involved in maintaining the stem cell niche: *SHOOT MERISTEMLESS*, *CLAVATA 1*, *A-TYPE RESPONSE REGULATOR 7* (*ARR7*), *ARR15*, and *ARGONAUTE 10* (Aichinger et al., 2012). Based on its antagonism with *ATH1*, *BOP1*, *BOP2*, and *KNAT6*, which are expressed at the boundary between lateral organs the stem, *RPL* has been proposed to oppose organ boundary development (Khan et al., 2015); accordingly, RPL interacted directly with the majority of known organ boundary genes, including *CUP-SHAPED COTYLEDONS 1* (*CUC1*), *CUC3*, *CUC*-repressing microRNAs (*miR164B* and *miR164C*), known downstream components of the *CUC* pathway *LATERAL ORGAN FUSION 1* (*LOF1*), *LOF2*, *LIGHT-DEPENDENT SHORT HYPOCOTYL 3* (*LSH3*), *LSH4*, and multiple members of the *LATERAL ORGAN BOUNDARIES* (*LOB*) *DOMAIN* family, including *LOB* and *JAGGED LATERAL ORGANS* (Žádníková and Simon, 2014; Hepworth and Pautot, 2015). Furthermore, the ChIP-seq results revealed links between *RPL* and a large number of genes involved in shoot organogenesis, including genes that control abaxial/adaxial identity, organ growth, cell cycle, cell-wall functions, and vascular development (Table S1). In summary, the ChIP-seq results placed *RPL* in a central hub connecting many of the key regulatory pathways in shoot development.

RPL Promotes Rib Meristem Function by Antagonizing the Organ Boundary Gene *LSH4*

Many of the target genes mentioned above are likely regulated by both *RPL* and its close homolog *PNF*, since these two genes function redundantly in meristem establishment and in the control of the floral transition. To narrow down the list of genes that could mediate the role of *RPL* in the RZ, we took advantage of the fact that some processes, such as stem elongation and fruit development, are preferentially affected in the *rpl* single mutant (Smith and Hake, 2003; Roeder et al., 2003; Byrne et al., 2003). To filter the ChIP-seq data for genes relevant to stem development, we looked for transcriptome changes in dissected inflorescence apices of *rpl-1* compared with the wild-type (Table S3). Although the majority of differences in mRNA abundance were expected to result from indirect, steady-state effects of *RPL*, the set of differentially expressed genes (DEGs) was significantly enriched for direct RPL targets based on ChIP-seq ($p = 1.64 \times 10^{-8}$, Fisher's exact test; Figure 5A). The 136 directly regulated targets included approximately equal numbers of upregulated (67) and downregulated genes (69) (Table S3), indicating that RPL can function as both a transcriptional activator and repressor. GO analysis of these genes showed clusters similar to those in the ChIP-seq experiments, highlighting meristem development and organogenesis, regulation of hormone levels, responses to hormones and external stimuli, and transport of ions and sugar (Figure 5B and

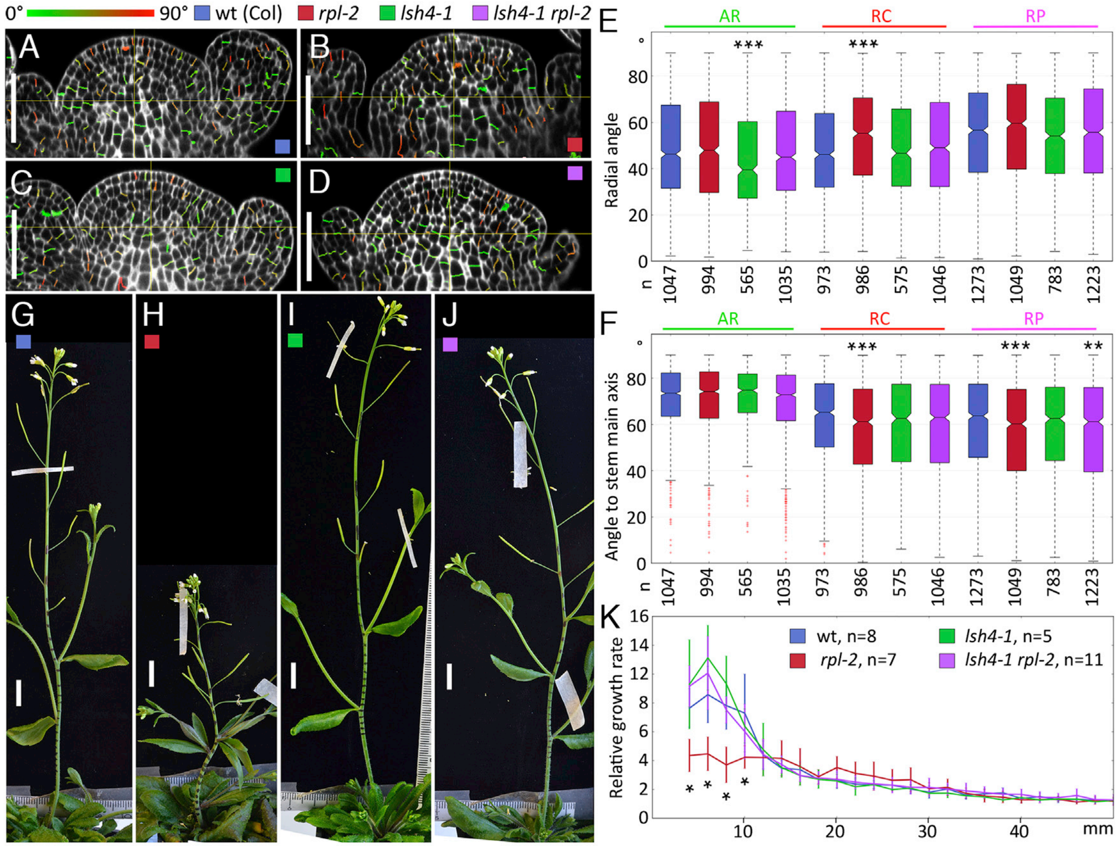

Figure 6. *LSH4* Expression Causes the *rpl* Defects in RZ Development and Stem Growth

(A–D) Longitudinal sections through confocal image stacks of inflorescence apices stained by mPS-PI, overlaid with images of segmented cell facets detected as new walls and colored (color scale above A) according to their radial orientation as in Figure 2A. (A) Wild-type (Columbia); (B) *rpl-2*; (C) *lsh4-1*; (D) *rpl-2 lsh4-1*.

(E and F) Boxplots showing the distribution of new wall radial angles (E) or angles to the main stem axis (F). Colors correspond to the genotypes indicated above (A and B). n indicates the number of new walls in each set (combined data from four apices for each genotype); asterisks indicate statistically significant differences (**$p < 0.01$, ***$p < 0.001$, Mann-Whitney test).

(G–J) Inflorescences of wild-type Columbia (G), *rpl-2* (H), *lsh4-1* (I), and *lsh4-1 rpl-2* (J), 4 days after the first flower self-pollinated and black marks were placed on the stem at 2-mm intervals to track growth rates.

(K) Relative growth of different stem regions, measured by tracking landmarks placed on the stem as in (G)–(J). The graph shows mean and SD; the number of replicates is indicated on the color legend for each genotype; asterisks indicate statistically significant differences relative to the wild-type (*$p < 0.01$, Student's t test). The horizontal axis shows the original distance of landmarks to the apex, before growth.

In the boxplots, the box extends from the lower to upper quartile values with a line at the median; whiskers extend to 1.5 times the interquartile range, and outlier points beyond the whiskers are shown in red. Scale bars, 50 μm (A–D) and 1 cm (G–J). See also Figure S6.

Table S4). The meristem and organogenesis cluster included components of the core RPL regulatory module (*ATH1*, *KNAT6*), genes that regulate meristem function (*STM*, *ARR7*, *AGO10*, *HAM3*), and genes implicated in organ boundary development (*LSH4*, *LOB*) (Table S3).

Of the known regulators of shoot development present in the set of directly regulated targets, *LSH4* showed the most significant differential expression (Table S3). We next focused on this gene, considering that *LSH4* functions downstream of *CUC* genes, which control not only organ boundary development but also stem development (Vroemen et al., 2003; Hibara et al., 2006). The higher expression of *LSH4* in the mutant, seen in the transcriptome profiling, was verified by RT-PCR, and similar results were obtained for *LOB* (Figure 5C). To determine the spatial localization of LSH4, we used a genomic fusion with GFP (*pLSH4*:*LSH4-GFP*) to visualize the expression pattern in apical meristems. In wild-type apices we observed GFP around the base of floral buds and in the peripheral region of the RZ (Figure 5D), similar to the previously described expression pattern for *LSH4* (Takeda et al., 2011). In contrast, in the *rpl-1* mutant, the region of *pLSH4*:*LSH4-GFP* expression extended into the central region of the RZ (Figure 5E). Comparable results were obtained with the *pCUC1*:*CUC1-GFP* organ boundary reporter (Baker et al., 2005), confirming that *RPL* represses a suite of organ boundary genes in the RZ (Figure S4).

To test the functional relevance of *LSH4* repression by RPL, we crossed the *rpl-2* and *lsh4-1* mutants (both strong alleles in the Columbia accession) (Takeda et al., 2011; Roeder et al., 2003). Similar to our observations for *rpl-1*, cells in the RZ appeared less ordered in *rpl-2* than in wild-type apices (Figures 6A and 6B), with significant differences in the orientation of new walls in the central region (RC), relative to both the radial

axes (Figure 6E) and the stem main axis (Figure 6F). We did not observe differences in the RC of the *lsh4-1* single mutant compared with the wild-type, although the mutant did show an increase in radially oriented divisions in the AR of the meristem (Figure 6E). In the double mutant *rpl-2 lsh4-1*, the RC was visibly more ordered than in *rpl-2* (Figure 6D) and the orientation of cell divisions was restored to the wild-type pattern in the RC, although not in the surrounding RP (Figures 6E and 6F). The defect in stem elongation of *rpl-2* was also suppressed in *rpl-2 lsh4-1* due to restoration of elongation rates close to the inflorescence apex (Figures 6G–6K), while defects in fruit development were not suppressed in the double mutant (Figure S5).

In conclusion, ectopic *LSH4* expression caused most of the defects in RZ function and stem growth seen in the *rpl* mutant. Mutations in *BOP1*, *BOP2*, *ATH1*, and *KNAT6* have been shown to restore all wild-type functions in the *rpl* mutant, including flowering time and fruit development, indicating that these genes function within the same central regulatory node as *RPL* (Khan et al., 2012, 2015; Ragni et al., 2008; Khan et al., 2015). In contrast, *lsh4-1* suppressed a subset of the *rpl-2* phenotypes, suggesting a more specialized role for organ boundary functions in the control of stem growth by *RPL*. In accordance with a role for *LSH4* downstream of the *BOP1*/*BOP2*/*ATH1*/*KNAT6* module, suppression of the *rpl-2* defects by *knat6-2* included restoration of *LSH4* repression and rescue of oriented divisions in the RZ (Figure S6). At the same time, the almost complete rescue of RZ function and stem growth in the *rpl lsh4* double mutant (Figure 6) suggests that if additional organ boundary genes are relevant to the control of RZ function by *RPL*, these genes function within a module that requires *LSH4* activity.

DISCUSSION

Our results provide insight into the 3D patterns of growth and cell division in the deep layers of the shoot apical meristem, a region crucial for the development of new stem tissues. We reveal that *RPL* controls RZ function not through the rate of cell growth and proliferation but by repressing organ boundary genes to allow the establishment of central and peripheral regions, which have characteristic patterns of oriented cell division.

Organ boundaries are considered regions of reduced growth (Hepworth and Pautot, 2015), so activation of boundary genes in the RZ could be expected to inhibit tissue growth (Žádníková and Simon, 2014; Hepworth and Pautot, 2015). However, our clonal analysis did not reveal reduced growth rates in tissues that expressed *LSH4*, i.e., in the RP region in the wild-type and *rpl-1*, or in the RC region of *rpl-1* (Figure 6). Instead, the most obvious change caused by *LSH4* was in the orientation of cell divisions. Ectopic *LSH4* expression in the *rpl* mutant may have narrowed the rib meristem because of the lower frequency of radial divisions observed in the RC region, where the rib meristem is initiated (Figures 2 and 6), or may have induced inappropriate radial divisions during subsequent growth of the rib meristem. Either way, within the region of the developing stem that overlaps the *RPL* expression domain (Smith and Hake, 2003) (Figure S3), the primary consequence of losing *RPL* function was not a reduction in overall growth, but a defect in establishing distinct central and peripheral regions. The reduced stem growth seen within a few millimeters of the apex in the *rpl* mutant is likely an indirect consequence of the early RZ defects. One possibility is that an abnormal or displaced boundary between the central and peripheral RZ might affect development of the stem vasculature and interfascicular fibers, which form at this boundary, are affected in the *rpl* mutant, and have been proposed to mechanically constrain stem elongation (Muñiz et al., 2008; Mele et al., 2003; Smith and Hake, 2003).

An important question is by which mechanism *RPL* and *LSH4* could affect oriented cell growth and division. Mechanical stress during tissue growth feeds back to influence the orientation of microtubule arrays and cell division (Hamant et al., 2008), so a possible mechanism would be that the visibly thicker cell walls of the rib meristem (e.g., Figures 2A and 6C) could impose mechanical constraints on the surrounding tissues. Alternatively, *RPL* and *LSH4* could modulate auxin signaling or transport. The orientation of cell divisions responds to auxin (Yoshida et al., 2014), perhaps through regulation of the molecular mechanism that orients the mitotic spindle, or perhaps indirectly by setting the direction of cell growth (Sassi and Traas, 2015). Furthermore, auxin transport is regulated at organ boundaries to create a low-auxin environment (Heisler et al., 2010; Wang et al., 2014; Hepworth and Pautot, 2015). A role in regulating auxin functions is also suggested by our ChIP-seq results showing direct interaction between RPL and multiple genes involved in auxin transport and signaling (Vanneste and Friml, 2009), e.g., *PIN-FORMED 5* (*PIN5*), *PIN6*, *LIKE AUXIN 1* (*LAX1*), *LAX3*, *AUXIN RESPONSIVE FACTOR 4* (*ARF4*), *ARF6*, *ARF8*, *ARF10*, *ARF11*, and *ARF17* (Table S1).

At first sight it could be expected that the rate of stem elongation would simply reflect the rate of cell growth and proliferation in the RZ, just as root elongation reflects the rate at which new cells are produced by the root meristem (Beemster and Baskin, 1998). Contrary to this expectation, our results emphasize the regulation of axial growth through orientation, rather than rates of cell growth and division. In an analogous way, it has been assumed that elongation of the vertebrate limb results from a proximodistal gradient of cell proliferation, but recent 3D imaging and mathematical modeling highlighted the role of oriented cell activities (Boehm et al., 2010). In addition to providing insight into the internal cell behavior required for growth of a 3D structure, our work opens the way to study and modify a developmental process that influences plant traits with key practical importance.

EXPERIMENTAL PROCEDURES

Plant Material

Plants were grown on JIC *Arabidopsis* Soil Mix at 16°C under continuous light (100 μE). *Arabidopsis thaliana* Landsberg-*erecta* (L-*er*) and Columbia (Col) were used as wild-types; *rpl-1* (Roeder et al., 2003), *rpl-2* (Roeder et al., 2003), *lsh4-1* (Takeda et al., 2011), *knat6-2* (Ragni et al., 2008), *pCUC1*:*CUC1-GFP* (Baker et al., 2005), and *hsp18.2*:*Cre* (Sieburth et al., 1998) have been described. Transgenic lines were generated by floral dip transformation (Clough and Bent, 1998).

PCR primers used to create DNA constructs are listed in Supplemental Experimental Procedures. For construction of *pRPL*:*RPL-GFP*, *RPL* was amplified from Col genomic DNA and fused in-frame with sGFP(S65T) (Chiu et al., 1996), and cloned into pPZP222 (Hajdukiewicz et al., 1994). For construction of *pLSH4*:*LSH4-GFP*, *LSH4* was amplified from Col genomic DNA and the sGFP(S65T) cDNA was inserted in-frame at the end of the *LSH4* coding sequence before assembly into pCambia 1300 (CAMBIA). The

35S:loxCFPloxGFP was created by Golden Gate cloning in the vector pAGM4723 (Addgene #48015) as described by Weber et al. (2011), using synthesized DNA (Lifetech) for the 35S promoter, *loxP* reverse, CFP-ER, 35S terminator, *loxP* reverse, GFP-ER, and the actin terminator (see Supplemental Experimental Procedures for sequences).

Imaging and Image Analysis

Dissection and live imaging of inflorescence apices, including time-lapse experiments, and imaging of apices stained by the modified pseudo-Schiff propidium iodide (mPS-PI) method were performed as described previously (Serrano-Mislata et al., 2015; Truernit and Haseloff, 2008). For generation of Cre-*lox*P sectors, plants hemizygous for *hsp18.2*:*Cre* and *35S*:*loxCFPloxGFP* were heat-shocked by immersing their inflorescence apices in a water bath at 38.5°C for 70 s and returned to standard growth conditions for 3 days before dissection and live imaging.

For 3D segmentation, cell measurements, and matching cells at different time points, 3D_meristem_analysis was used (Serrano-Mislata et al., 2015), with additional scripts added to detect and analyze the 3D orientation of new cell walls, to landmark, align, and measure Cre-*lox*P sectors from different apices (Data S1).

Chromatin Immunoprecipitation/High-Throughput Sequencing and Data Analysis

ChIP was performed on dissected inflorescence apices as described by Schiessl et al. (2014) (details in Supplemental Experimental Procedures). Six Illumina TruSeq ChIP-seq libraries (three *pRPL*: *RPL-GFP* replicates and three wild-type controls) were produced as described by Kaufmann et al. (2009) and sequenced (50-bp single-end reads) using a HiSeq 2500 (Rapid-Run mode) as described by the manufacturer (Illumina). Reads from three replicate treatments and three replicate controls were aligned against the TAIR10 Col-0 reference sequence with Bowtie2 (v2-2.1.0; Langmead and Salzberg, 2012), data were sorted and indexed with SAMtools (Li et al., 2009), and MACS 2.0.10 (Feng et al., 2012) was used to call peaks and calculate fold enrichments and q values, comparing the combined replicates with combined controls. For selection of peaks that were consistently detected across replicates, peak calling with MACS 2.0.10 was applied to individual replicates and overlapping peak regions were accepted if they had q values of 10^{-3} or lower in each *RPL*:*RPL-GFP* replicate, were not detected in any of the negative controls, and the overlapping region was at least 50 nt long. After this filtering step, peaks were attributed to gene models within 3 kb upstream or 1.5 kb downstream of the corresponding coding sequence, without intervening coding sequences. For peak overlaps and association to gene models, the script Overlap_MACS2_files.py was used (details in annotated source code, associated gene models, and annotation tables in Data S2). ChIP-seq data were visualized using the Integrative Genomics Viewer (Robinson et al., 2011).

To analyze the distribution of peaks within genes we used the script *peak_statistics.py*, which also includes details of the Monte Carlo method used to estimate the p value for the hypothesis that these frequencies correspond to a random distribution of peaks within genes (Data S2). For detection of enrichment for sequence motifs, MEME-ChIP (http://meme-suite.org/tools/meme-chip) (Bailey et al., 2009) was used in discriminative mode, comparing the sequences around observed peaks with a control set of sequences around a 10-fold larger number of random peaks; both sets were produced with script *peak_sequences.py* (Data S2). To test for overrepresented GO terms, we used the hypergeometric test of the GOstats package (Falcon and Gentleman, 2007) with the org.At.tair.db annotation package (Gentleman et al., 2004), and Revigo (Supek et al., 2011) was used to cluster enriched terms.

Raw and processed data have been deposited in the NCBI Gene Expression Omnibus (Edgar et al., 2002) under accession number GEO: GSE78727.

Transcriptome Analysis

Ten micrograms of RNA was extracted from inflorescence apices of wild-type L-*er* and *rpl-1* (three replicates each) with Trizol (Sigma) and purified using Qiagen RNAeasy columns (Qiagen) according to the manufacturer's instructions. AGRONOMICS1 arrays (Affymetrix) were hybridized with labeled cDNA following the manufacturer's instructions. To detect DEGs, we used the affylmGUI package (http://bioinf.wehi.edu.au/affylmGUI/about.html) to run LIMMA (Linear Models for MicroArray data) (Smyth, 2005) using a chip description format (CDF) file for the AGRONOMICS1 array and TAIR10 (Müller et al., 2012). Raw and processed data have been deposited in the NCBI Gene Expression Omnibus (Edgar et al., 2002) under accession number GEO: GSE78511.

qPCR

qRT-PCR was performed as published by Schiessl et al. (2012) (details in Supplemental Experimental Procedures).

Measurements of Stem Growth

Plants were grown as described above; when the first flower self-pollinated, ink dots were manually placed on the stem at 2-mm intervals and photographed next to a ruler. After a further 4 days of growth, the stems were photographed again. The ink marks and positions on the rules were landmarked manually on the images using the Point Picker plugin of Fiji (Schindelin et al., 2012). Distances between landmark coordinates were measured, graphs were plotted, and Mann-Whitney U tests and Student's t tests were performed using standard functions in matplotlib (http://matplotlib.org), Python 2.7, and Scientific Python (http://www.scipy.org).

ACCESSION NUMBERS

Raw and processed data have been deposited in the NCBI Gene Expression Omnibus under accession numbers GEO: GSE78727 and GSE78511.

SUPPLEMENTAL INFORMATION

Supplemental Information includes Supplemental Experimental Procedures, six figures, four tables, and two data files and can be found with this article online at http://dx.doi.org/10.1016/j.devcel.2016.08.013.

AUTHOR CONTRIBUTIONS

Conceptualization, S.B. and R.S.; Investigation, S.B., A.S.-M., and M.B.; Resources, S.F.; Software, Formal Analysis, and Data Curation, R.S.; Writing – Original Draft, R.S.; Writing – Review & Editing, S.B., S.F., A.S.-M., M.B., and R.S.; Funding Acquisition, R.S; Supervision, R.S.

ACKNOWLEDGMENTS

We thank Mitsuhiro Aida (Nara Institute of Science and Technology, Japan) for *lsh4-1* seeds, Shelley Hepworth (Carleton University, Canada) for *knat6-2 rpl-2* seeds, Martin Trick for help with the initial ChIP-seq analysis, and Katharina Schiessl, Bihai Shi, Vishmita Sethi, and Lars Østergaard for critical comments. The work was supported by BBSRC grants BB/J007056/1 and BB/J004588/1.

Received: March 3, 2016
Revised: April 14, 2016
Accepted: August 24, 2016
Published: September 22, 2016

REFERENCES

Aichinger, E., Kornet, N., Friedrich, T., and Laux, T. (2012). Plant stem cell niches. Annu. Rev. Plant Biol. *63*, 615–636.

Andrés, F., Romera-Branchat, M., Martínez-Gallegos, R., Patel, V., Schneeberger, K., Jang, S., Altmüller, J., Nürnberg, P., and Coupland, G. (2015). Floral induction in *Arabidopsis* by FLOWERING LOCUS T requires direct repression of BLADE-ON-PETIOLE genes by the homeodomain protein PENNYWISE. Plant Physiol. *169*, 2187–2199.

Arnaud, N., Lawrenson, T., Østergaard, L., and Sablowski, R. (2011). The same regulatory point mutation changed seed-dispersal structures in evolution and domestication. Curr. Biol. *21*, 1215–1219.

Bailey, T.L., Boden, M., Buske, F.A., Frith, M., Grant, C.E., Clementi, L., Ren, J., Li, W.W., and Noble, W.S. (2009). MEME suite: tools for motif discovery and searching. Nucleic Acids Res. *37*, W202.

Baker, C.C., Sieber, P., Wellmer, F., and Meyerowitz, E.M. (2005). The early extra petals1 mutant uncovers a role for microRNA miR164c in regulating petal number in arabidopsis. Curr. Biol. *15*, 303–315.

Beemster, G., and Baskin, T. (1998). Analysis of cell division and elongation underlying the developmental acceleration of root growth in *Arabidopsis thaliana*. Plant Physiol. *116*, 1515–1526.

Bellaoui, M., Pidkowich, M.S., Samach, A., Kushalappa, K., Kohalmi, S.E., Modrusan, Z., Crosby, W.L., and Haughn, G.W. (2001). The *Arabidopsis* BELL1 and KNOX TALE homeodomain proteins interact through a domain conserved between plants and animals. Plant Cell *13*, 2455–2470.

Besson, S., and Dumais, J. (2011). Universal rule for the symmetric division of plant cells. Proc. Natl. Acad. Sci. USA *108*, 6294–6299.

Boehm, B., Westerberg, H., Lesnicar-Pucko, G., Raja, S., Rautschka, M., Cotterell, J., Swoger, J., and Sharpe, J. (2010). The role of spatially controlled cell proliferation in limb bud morphogenesis. PLoS Biol. *8*, e1000420.

Byrne, M.E., Groover, A.T., Fontana, J.R., and Martienssen, R.A. (2003). Phyllotactic pattern and stem cell fate are determined by the *Arabidopsis* homeobox gene BELLRINGER. Development *130*, 3941–3950.

Chiu, W., Niwa, Y., Zeng, W., Hirano, T., Kobayashi, H., and Sheen, J. (1996). Engineered GFP as a vital reporter in plants. Curr. Biol. *6*, 325–330.

Clough, S.J., and Bent, A.F. (1998). Floral dip: a simplified method for *Agrobacterium*-mediated transformation of *Arabidopsis thaliana*. Plant J. *16*, 735–743.

Edgar, R., Domrachev, M., and Lash, A.E. (2002). Gene Expression Omnibus: NCBI gene expression and hybridization array data repository. Nucleic Acids Res. *30*, 207–210.

Falcon, S., and Gentleman, R. (2007). Using GOstats to test gene lists for GO term association. Bioinformatics *23*, 257–258.

Feng, J., Liu, T., Qin, B., Zhang, Y., and Liu, X.S. (2012). Identifying ChIP-seq enrichment using MACS. Nat. Protoc. *7*, 1728–1740.

Fletcher, J.C. (2002). Shoot and floral meristem maintenance in arabidopsis. Annu. Rev. Plant Biol. *53*, 45–66.

Gallois, J.L., Nora, F.R., Mizukami, Y., and Sablowski, R. (2004). WUSCHEL induces shoot stem cell activity and developmental plasticity in the root meristem. Genes Dev. *18*, 375.

Gentleman, R.C., Carey, V.J., Bates, D.M., Bolstad, B., Dettling, M., Dudoit, S., Ellis, B., Gautier, L., Ge, Y., and Gentry, J. (2004). Bioconductor: open software development for computational biology and bioinformatics. Genome Biol. *5*, R80.

Hajdukiewicz, P., Svab, Z., and Maliga, P. (1994). The small, versatile pPZP family of *Agrobacterium* binary vectors for plant transformation. Plant Mol. Biol. *25*, 989–994.

Hamant, O., Heisler, M., Jonsson, H., Krupinski, P., Uyttewaal, M., Bokov, P., Corson, F., Sahlin, P., Boudaoud, A., Meyerowitz, E., et al. (2008). Developmental patterning by mechanical signals in *Arabidopsis*. Science *322*, 1650–1655.

Heisler, M.G., Hamant, O., Krupinski, P., Uyttewaal, M., Ohno, C., Jönsson, H., Traas, J., and Meyerowitz, E.M. (2010). Alignment between PIN1 polarity and microtubule orientation in the shoot apical meristem reveals a tight coupling between morphogenesis and auxin transport. PLoS Biol. *8*, e1000516.

Hepworth, S., and Pautot, V. (2015). Beyond the divide: boundaries for patterning and stem cell regulation in plants. Front. Plant Sci. *6*, 1052.

Hibara, K.-I., Karim, M.R., Takada, S., Taoka, K.-I., Furutani, M., Aida, M., and Tasaka, M. (2006). *Arabidopsis* CUP-SHAPED COTYLEDON3 regulates postembryonic shoot meristem and organ boundary formation. Plant Cell *18*, 2946–2957.

Kaufmann, K., Muiño, J.M., Jauregui, R., Airoldi, C.A., Smaczniak, C., Krajewski, P., and Angenent, G.C. (2009). Target genes of the MADS transcription factor SEPALLATA3: integration of developmental and hormonal pathways in the *Arabidopsis* flower. PLoS Biol. *7*, e90.

Khan, M., Xu, M., Murmu, J., Tabb, P., Liu, Y., Storey, K., Mckim, S.M., Douglas, C.J., and Hepworth, S.R. (2012). Antagonistic interaction of BLADE-on-PETIOLE1 and 2 with BREVIPEDICELLUS and PENNYWISE regulates *Arabidopsis* inflorescence architecture. Plant Physiol. *158*, 946–960.

Khan, M., Ragni, L., Tabb, P., Salasini, B.C., Chatfield, S., Datla, R., Lock, J., Kuai, X., Després, C., and Proveniers, M. (2015). Repression of lateral organ boundary genes by PENNYWISE and POUND-FOOLISH is essential for meristem maintenance and flowering in *Arabidopsis*. Plant Physiol. *169*, 2166–2186.

Khush, G.S. (2001). Green revolution: the way forward. Nat. Rev. Genet. *2*, 815–822.

Kwiatkowska, D. (2004). Structural integration at the shoot apical meristem: models, measurements, and experiments. Am. J. Bot. *91*, 1277–1293.

Lal, S., Pacis, L.B., and Smith, H.M. (2011). Regulation of the SQUAMOSA PROMOTER-BINDING PROTEIN-LIKE genes/microRNA156 module by the homeodomain proteins PENNYWISE and POUND-FOOLISH in *Arabidopsis*. Mol. Plant *4*, 1123–1132.

Langmead, B., and Salzberg, S.L. (2012). Fast gapped-read alignment with Bowtie 2. Nat. Methods *9*, 357–359.

Li, H., Handsaker, B., Wysoker, A., Fennell, T., Ruan, J., Homer, N., Marth, G., Abecasis, G., and Durbin, R. (2009). The sequence alignment/map format and SAMtools. Bioinformatics *25*, 2078–2079.

Mele, G., Ori, N., Sato, Y., and Hake, S. (2003). The knotted1-like homeobox gene BREVIPEDICELLUS regulates cell differentiation by modulating metabolic pathways. Genes Dev. *17*, 2088–2093.

Müller, M., Patrignani, A., Rehrauer, H., Gruissem, W., and Hennig, L. (2012). Evaluation of alternative RNA labeling protocols for transcript profiling with *Arabidopsis* AGRONOMICS1 tiling arrays. Plant Methods *8*, 1.

Muñiz, L., Minguet, E.G., Singh, S.K., Pesquet, E., Vera-Sirera, F., Moreau-Courtois, C.L., Carbonell, J., Blázquez, M.A., and Tuominen, H. (2008). ACAULIS5 controls *Arabidopsis* xylem specification through the prevention of premature cell death. Development *135*, 2573–2582.

Ragni, L., Belles-Boix, E., Günl, M., and Pautot, V. (2008). Interaction of KNAT6 and KNAT2 with BREVIPEDICELLUS and PENNYWISE in *Arabidopsis* inflorescences. Plant Cell *20*, 888–900.

Robinson, J.T., Thorvaldsdóttir, H., Winckler, W., Guttman, M., Lander, E.S., Getz, G., and Mesirov, J.P. (2011). Integrative genomics viewer. Nat. Biotechnol. *29*, 24–26.

Roeder, A.H.K., Ferrandiz, C., and Yanofsky, M.F. (2003). The role of the REPLUMLESS homeodomain protein in patterning the *Arabidopsis* fruit. Curr. Biol. *13*, 1630–1635.

Sachs, R.M. (1965). Stem elongation. Annu. Rev. Plant Physiol. *16*, 73–95.

Sanchez, P., Nehlin, L., and Greb, T. (2012). From thin to thick: major transitions during stem development. Trends Plant Sci. *17*, 113–121.

Sassi, M., and Traas, J. (2015). When biochemistry meets mechanics: a systems view of growth control in plants. Curr. Opin. Plant Biol. *28*, 137–143.

Schiessl, K., Kausika, S., Southam, P., Bush, M., and Sablowski, R. (2012). JAGGED controls growth anisotropy and coordination between cell size and cell cycle during plant organogenesis. Curr. Biol. *22*, 1739–1746.

Schiessl, K., Muiño, J.M., and Sablowski, R. (2014). *Arabidopsis* JAGGED links floral organ patterning to tissue growth by repressing Kip-related cell cycle inhibitors. Proc. Natl. Acad. Sci. USA *111*, 2830–2835.

Schindelin, J., Arganda-Carreras, I., Frise, E., Kaynig, V., Longair, M., Pietzsch, T., Preibisch, S., Rueden, C., Saalfeld, S., and Schmid, B. (2012). Fiji: an open-source platform for biological-image analysis. Nat. Methods *9*, 676–682.

Serrano-Mislata, A., Schiessl, K., and Sablowski, R. (2015). Active control of cell size generates spatial detail during plant organogenesis. Curr. Biol. *25*, 2991–2996.

Sieburth, L.E., Drews, G.N., and Meyerowitz, E.M. (1998). Non-autonomy of AGAMOUS function in flower development: use of a Cre/loxP method for mosaic analysis in *Arabidopsis*. Development *125*, 4303–4312.

Smaczniak, C., Immink, R.G., Muiño, J.M., Blanvillain, R., Busscher, M., Busscher-Lange, J., Dinh, Q.P., Liu, S., Westphal, A.H., Boeren, S., et al. (2012). Characterization of MADS-domain transcription factor complexes in *Arabidopsis* flower development. Proc. Natl. Acad. Sci. USA *109*, 1560–1565.

Smith, L.G. (2001). Plant cell division: building walls in the right places. Nat. Rev. Mol. Cell Biol. *2*, 33–39.

Smith, H.M.S., and Hake, S. (2003). The interaction of two homeobox genes, BREVIPEDICELLUS and PENNYWISE, regulates internode patterning in the *Arabidopsis* inflorescence. Plant Cell *15*, 1717–1727.

Smith, H.M.S., Boschke, I., and Hake, S. (2002). Selective interaction of plant homeodomain proteins mediates high DNA-binding affinity. Proc. Natl. Acad. Sci. USA *99*, 9579–9584.

Smith, H.M.S., Campbell, B.C., and Hake, S. (2004). Competence to respond to floral inductive signals requires the homeobox genes PENNYWISE and POUND-FOOLISH. Curr. Biol. *14*, 812–817.

Smyth, G.K. (2005). Limma: linear models for microarray data. In Bioinformatics and Computational Biology Solutions Using R and Bioconductor, R. Gentleman, V. Carey, S. Dudoit, R. Irizarry, and W. Huber, eds. (New York: Springer), pp. 397–420.

Supek, F., Bošnjak, M., Škunca, N., and Šmuc, T. (2011). Revigo summarizes and visualizes long lists of gene ontology terms. PLoS One *6*, e21800.

Takeda, S., Hanano, K., Kariya, A., Shimizu, S., Zhao, L., Matsui, M., Tasaka, M., and Aida, M. (2011). CUP-SHAPED COTYLEDON1 transcription factor activates the expression of LSH4 and LSH3, two members of the ALOG gene family, in shoot organ boundary cells. Plant J. *66*, 1066–1077.

Truernit, E., and Haseloff, J. (2008). A simple way to identify non-viable cells within living plant tissue using confocal microscopy. Plant Methods *4*, 15.

Truernit, E., Bauby, H., Dubreucq, B., Grandjean, O., Runions, J., Barthelemy, J., and Palauqui, J.-C. (2008). High-resolution whole-mount imaging of three-dimensional tissue organization and gene expression enables the study of phloem development and structure in *Arabidopsis*. Plant Cell *20*, 1494–1503.

Vanneste, S., and Friml, J. (2009). Auxin: a trigger for change in plant development. Cell *136*, 1005–1016.

Vroemen, C.W., Mordhorst, A.P., Albrecht, C., Kwaaitaal, M.A., and De Vries, S.C. (2003). The CUP-SHAPED COTYLEDON3 gene is required for boundary and shoot meristem formation in *Arabidopsis*. Plant Cell *15*, 1563–1577.

Wang, Y., Wang, J., Shi, B., Yu, T., Qi, J., Meyerowitz, E.M., and Jiao, Y. (2014). The stem cell niche in leaf axils is established by auxin and cytokinin in *Arabidopsis*. Plant Cell *26*, 2055–2067.

Weber, E., Engler, C., Gruetzner, R., Werner, S., and Marillonnet, S. (2011). A modular cloning system for standardized assembly of multigene constructs. PLoS One *6*, e16765.

Yoshida, S., De Reuille, P.B., Lane, B., Bassel, G.W., Prusinkiewicz, P., Smith, R.S., and Weijers, D. (2014). Genetic control of plant development by overriding a geometric division rule. Dev. Cell *29*, 75–87.

Žádníková, P., and Simon, R. (2014). How boundaries control plant development. Curr. Opin. Plant Biol. *17*, 116–125.

Developmental Cell, Volume *39*

Supplemental Information

Control of Oriented Tissue Growth

through Repression of Organ Boundary Genes

Promotes Stem Morphogenesis

Stefano Bencivenga, Antonio Serrano-Mislata, Max Bush, Samantha Fox, and Robert Sablowski

Supplemental Information

Inventory:

Figure S1: Recent divisions in inner meristem layers detected by cell tracking and corresponding new cell walls detected based on mPS-PI signal. Overlap of confocal images and segmentation images of tracked cells and new cell walls; related to Figure 1.

Figure S2: Differences in cell division orientation in the rib zone are consistent across individual apices. Boxplots, statistical analysis of division angles and confocal images of inflorescence apices, related to Figure 2.

Figure S3 *pRPL:RPL-GFP* complements *rpl-1* and is expressed in the the RZ. Confocal images of inflorescence apices and fruit expressing *pRPL:RPL-GFP* and images of wild-type, *rpl* mutant and complemented plants; related to Figure 4.

Figure S4: The boundary marker *pCUC1:CUC1-GFP* is ectopically expressed in the RZ of *rpl-1*. Confocal images of inflorescence apices, related to Figure 6.

Figure S5: The *lsh4-1* mutation did not suppress the replum phenotype of *rpl-2* mutant. Scanning electron micrographs of developing fruits and histogram of replum width in wild-type, *rpl*, *lsh4* and *rpl lsh4* mutants; related to Figure 6.

Figure S6: Suppression of *rpl-2* by *knat6-2* includes restoration of *LSH4* repression and of cell division orientation in the rib zone. Related to Figure 6.

Supplemental Table legends

Supplemental Data File legends

Supplemental Experimental Procedures

Supplemental References

Figure S1: Recent divisions in inner meristem layers detected by cell tracking and corresponding new cell walls detected based on mPS-PI signal. Related to Figure 1.

The orthogonal views correspond to image stacks of a wild-type Arabidopsis inflorescence apex; the yellow cross-hairs mark the same point in the top and side views; size bars: 50 µm. A, B: tracking of cell divisions in the live meristem imaged at 0h (A) and 24h later (B); cells that could be tracked are shown in the same color in the two images; green asterisks indicate cells of layers 2 or 3 that divided. C, D: Matching of cells from live imaging at 0h (C) and imaged by mPS-PI at 24h (D); matching cells are shown in the same color and asterisks indicate cells that divided. E, F: images corresponding to E, F, but showing only the FM4-64 signal for the live image (E) and showing cell facets detected as recent walls (F); asterisks indicate the new walls corresponding to the cell divisions indicated in A-D.

Figure S1

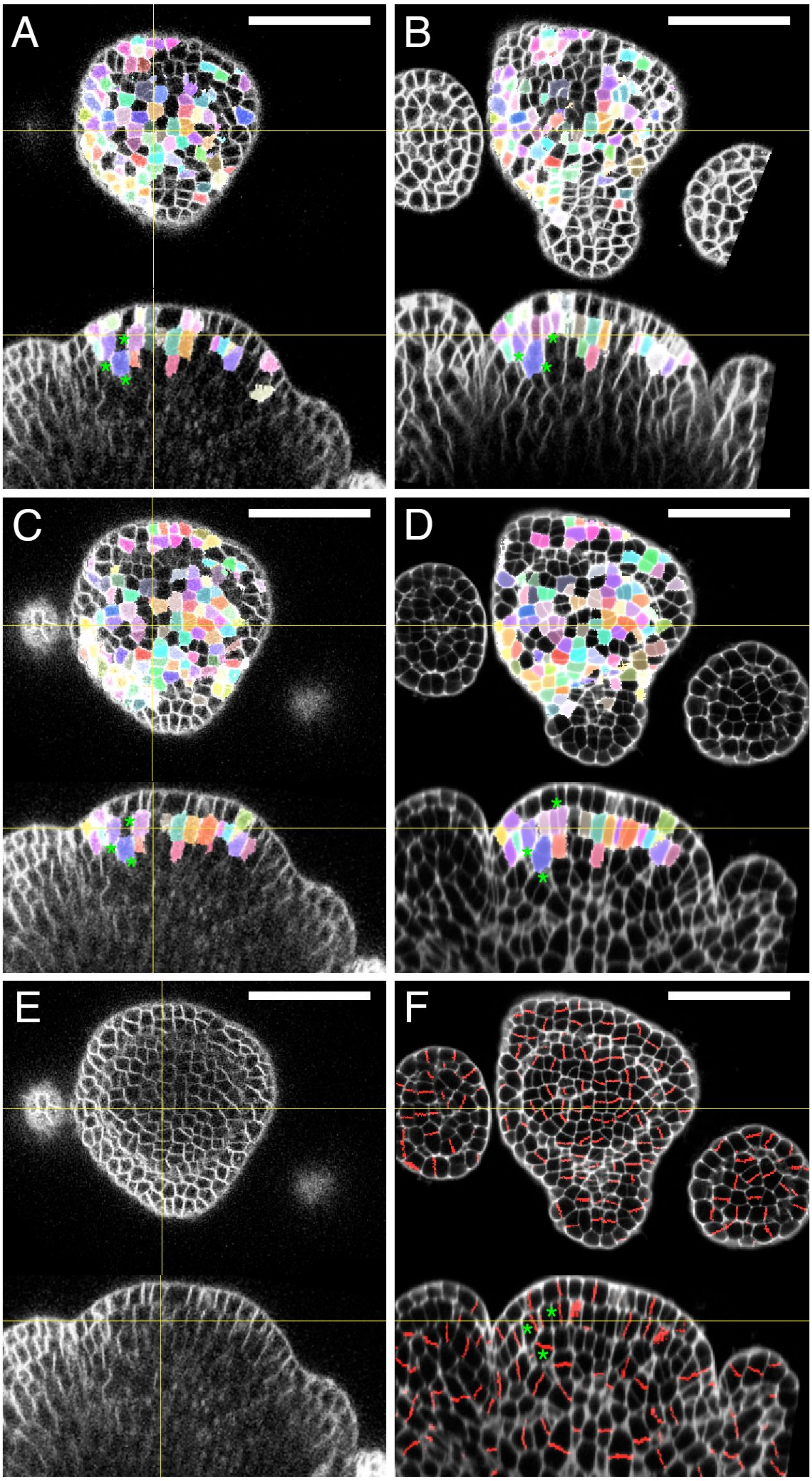

Figure S2: Differences in cell division orientation in the rib zone are consistent across individual apices. Related to Figure 2.

A: Analysis of the orientation of new cell walls in six wild-type (L-*er*) and four *rpl-1* apices were analysed. In the boxplots, the box extends from the lower to upper quartile values, with a line at the median, and whiskers extend to 1.5 times the interquartile range. The Kruskal-Wallis test *p*-values are for the null hypothesis that all apices have the same median orientation of cell divisions. The Mann-Whitney test *p*-values are for the null hypothesis that a particular replicate has the same median as the combined replicates for the other genotype (i.e., each wt was compared with the combined *rpl* data, and each *rpl* was compared with the combined wt data). AR, RC and RP correspond to the meristem regions shown in Figure 2C.

B, C: : Orthogonal views of confocal image stacks of inflorescence apices stained by mPS-PI. In each image, the yellow cross-hairs mark the same point in the top and side views. B: three apices each of wild-type (L-*er* and Col). C: three apices each of two different *rpl* alleles in the L-*er* and Col backgrounds (*rpl-1* and *rpl-2*, respectively). Note the less organized RZ (encircled region) in the *rpl* mutants compared to wt controls.

Figure S2

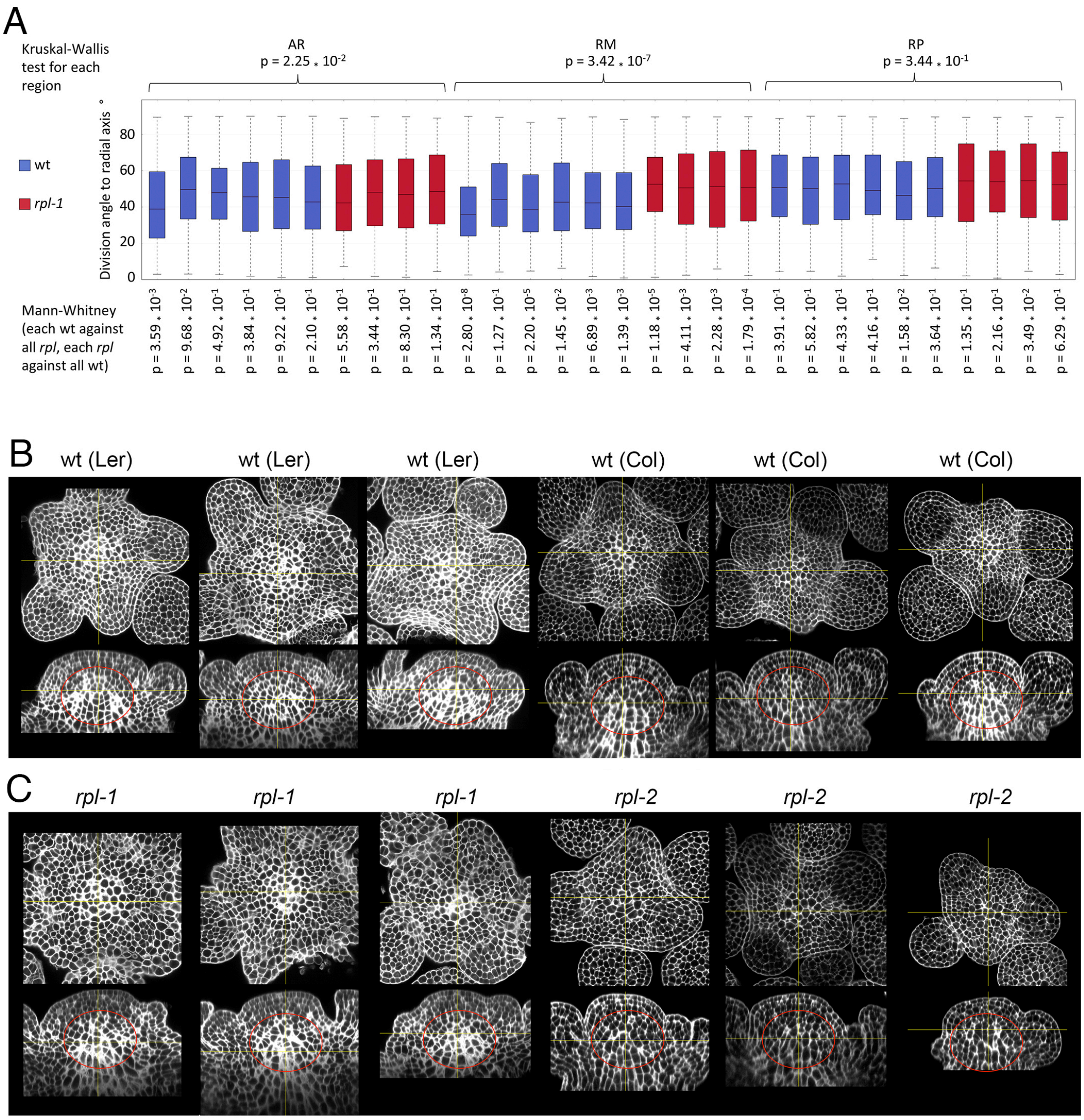
A
Kruskal-Wallis test for each region
AR
p = 2.25 * 10-2
RM
p = 3.42 * 10-7
RP
p = 3.44 * 10-1
wt
rpl-1
Division angle to radial axis °
80
60
40
20
0
Mann-Whitney (each wt against all rpl, each rpl against all wt)
p = 3.59 * 10-3
p = 9.68 * 10-2
p = 4.92 * 10-1
p = 3.84 * 10-1
p = 9.22 * 10-1
p = 2.10 * 10-1
p = 5.58 * 10-1
p = 3.44 * 10-1
p = 8.30 * 10-1
p = 1.34 * 10-1
p = 2.80 * 10-8
p = 1.27 * 10-1
p = 2.20 * 10-5
p = 1.45 * 10-2
p = 6.89 * 10-3
p = 1.39 * 10-3
p = 1.18 * 10-5
p = 4.11 * 10-3
p = 2.28 * 10-3
p = 1.79 * 10-4
p = 3.91 * 10-1
p = 5.82 * 10-1
p = 4.33 * 10-1
p = 4.16 * 10-1
p = 1.58 * 10-2
p = 3.64 * 10-1
p = 1.35 * 10-1
p = 2.16 * 10-1
p = 3.49 * 10-2
p = 6.29 * 10-1
B
wt (Ler)
wt (Ler)
wt (Ler)
wt (Col)
wt (Col)
wt (Col)
C
rpl-1
rpl-1
rpl-1
rpl-2
rpl-2
rpl-2

Figure S3:

pRPL:RPL-GFP complements *rpl-1* and is expressed in the the RZ. Related to Figure 4.

A: confocal images of *pRPL:RPL-GFP rpl-1* inflorescence apex showing expression in the subapical region of the meristem; the yellow cross-hairs mark the same point in the orthogonal views. B: expression in the replum of *pRPL:RPL-GFP rpl-1* silique.

C-E: Arabidopsis plants at the stage when the third silique has elongated; C: wild type (Landsberg-*erecta*); D: *rpl-1*; E: *RPL:RPL-GFP rpl-1*. Scale bar: 50 µm (A, B), 6mm (C-E).

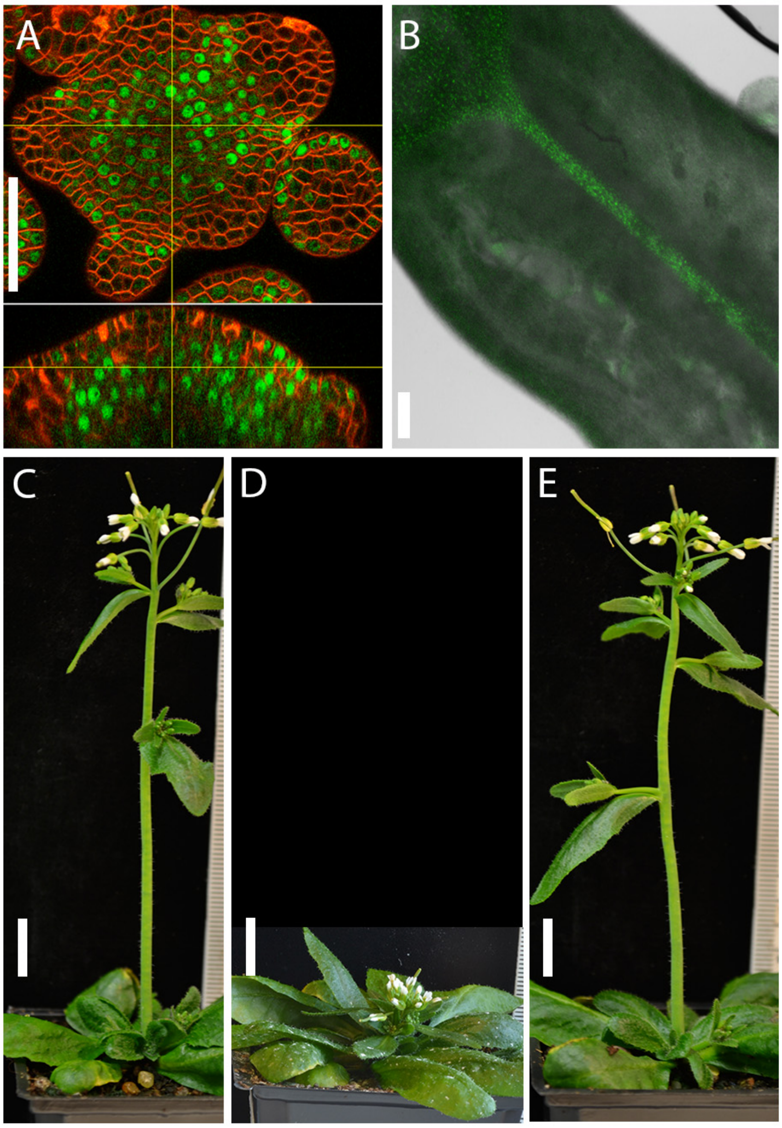

Figure S4: the boundary marker *pCUC1:CUC1-GFP* is ectopically expressed in the RZ of *rpl-1*. Related to Figure 6.

Orthogonal views of confocal image stacks showing *pCUC1:CUC1-GFP* expression in wild-type (A) and *rpl-1* (B) inflorescence apices; in each image, the yellow cross-hairs mark the same point in the top and side views. Scale bar: 50 µm

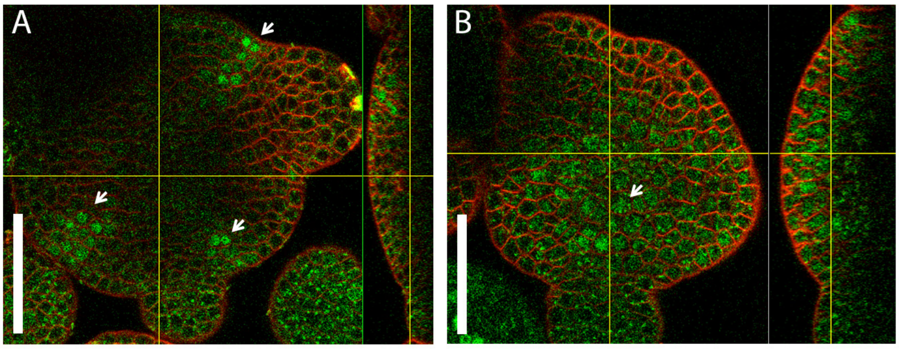

Figure S5: the *lsh4-1* mutation did not suppress the replum phenotype of *rpl-2* mutant. Related to Figure 6.

A-D: SEM images of developing siliques, showing the valves (V) and the replum (R) in the wild type (A), *rpl-2* (B), *lsh4-1* (C) and *rpl-2 lsh4-1* (D); white lines and black boxes show measurements of replum width. E: average (bars) and standard deviation (lines) for replum width in the genotypes shown in A-D. Scale bars: 20 µm.

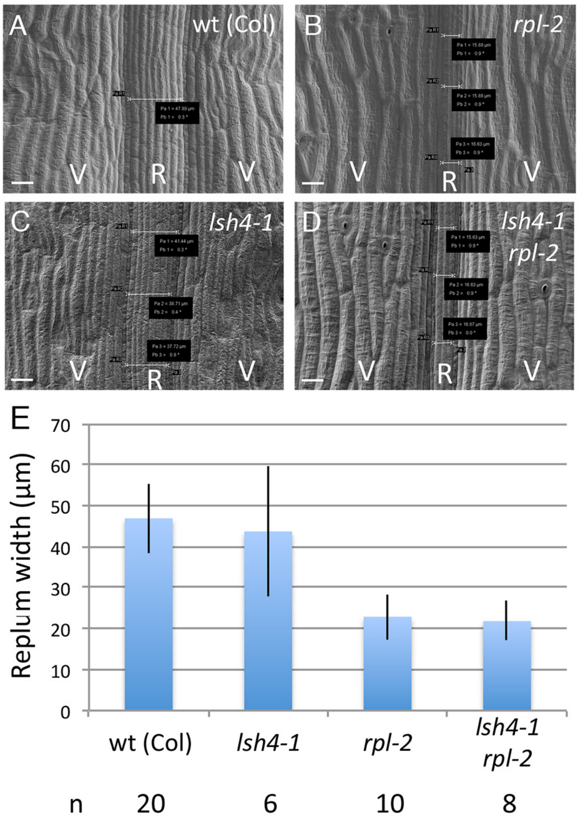

Figure S6: Suppression of *rpl-2* by *knat6-2* includes restoration of *LSH4* repression and of cell division orientation in the rib zone. Related to Figure 6.

A-B: representative inflorescences of wild type Columbia (A) and *knat6-2 rpl-2* (B) after the first 2-3 flowers matured. C-D: longitudinal sections through confocal image stacks of inflorescence apices of wild-type (Columbia)(C) and *knat6-2 rpl*-2 (D), with new walls colored according to radial orientation as in Figure 2A (compare with wt and *rpl-2* in Figure 6 A,B); E: expression of *LSH4* in inflorescence apices of *rpl-2*, wt (Columbia) and *knat6-2 rpl-2*, measured by qRT-PCR (average and standard deviation of 3 biological replicates per genotype; asterisk indicates significant difference to wt, $p < 0.05$, *t*-test). F-G: boxplots showing the distribution of new wall angles to the radial axis (F) and main stem axis (G) (compare with wt and *rpl-2* in Figure 6 E,F); colors correspond to the genotypes indicated in E; n indicates the number of new walls in each set (combined data from 4-5 apices for each genotype); asterisks indicate statistically significant differences ($p < 0.001$, Mann-Whitney test). Scale bars: 1 cm (A, B), 50 µm (C, D). In the boxplots, the box extends from the lower to upper quartile values with a line at the median; whiskers extend to 1.5 times the interquartile range and outlier points beyond the whiskers are shown in red.

Figure S6

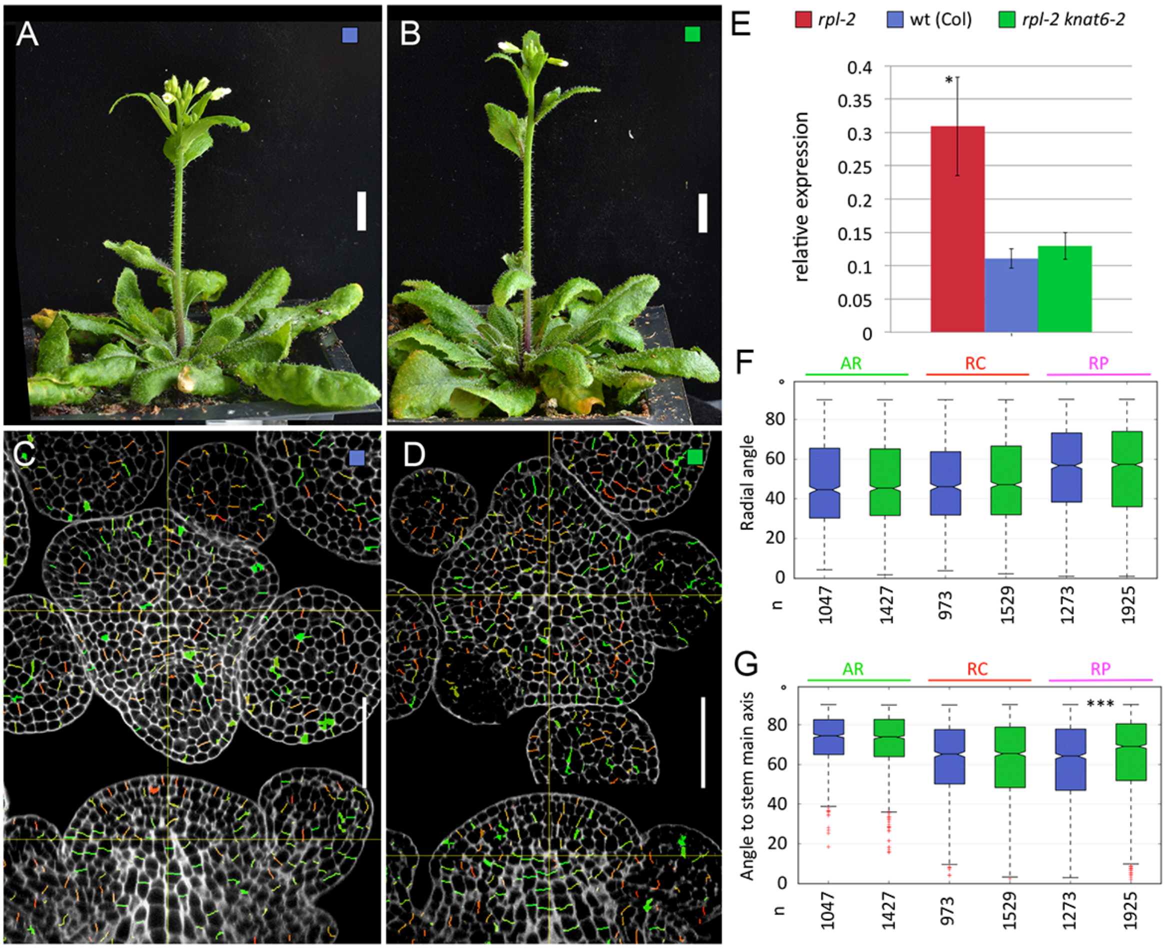

A
B
C
D
E
rpl-2
wt (Col)
rpl-2 knat6-2
relative expression
0.4
0.35
0.3
0.25
0.2
0.15
0.1
0.05
0
*
F
AR
RC
RP
Radial angle
°
80
60
40
20
0
n
1047
1427
973
1529
1273
1925
G
AR
RC
RP
Angle to stem main axis
***
°
80
60
40
20
0
n
1047
1427
973
1529
1273
1925

Supplemental Table legends

Table S1: High-confidence RPL ChIP-seq targets; genes considered as positive controls due to known interactions with RPL are marked in red (Related to Figure 4).

Table S2: Enrichment of GO terms (biological process) in the set of high-confidence RPL ChIP-seq targets (Related to Figure 4).

Table S3: Genes that were differentially expressed between wild-type and *rpl-1* inflorescences. A: Differentially expressed genes also present in the list of high-confidence ChIP-seq targets (Table S1). B: Differentially expressed genes absent from the list of high-confidence ChIP-seq targets in Table S1. (Related to Figure 5).

Table S4: Enrichment of GO terms (biological process) in the overlap between high-confidence ChIP-seq targets and genes that showed differential expression between *rpl-1* and the wild type. (Related to Figure 5).

Supplemental Data File legends

Supplemental Software 1: zip file containing annotated source code and instructions for installation and use of scripts used for image analysis; related to Experimental Procedures.

Supplemental Software 2: zip file containing annotated source code, instructions for installation and use of scripts for ChIP-seq analysis and gene annotations used; related to Experimental Procedures.

Supplemental experimental procedures

DNA sequences used for constructs

To produce *35S:loxCFPloxGFP,* the full sequence of the synthetic DNA inserted into pAGM4723 (Addgene) is shown below, with each component color-coded as follows:

35S promoter, loxP reverse, CYPET-ER, 35S terminator, GFP-ER, Actin terminator, Golden Gate scar sequences.

5’gtcaacatggtggagcacgacactctggtctactccaaaaatgtcaaagatacagtctcagaagatcaaagggctattgagactt
ttcaacaaaggataatttcgggaaacctcctcggattccattgcccagctatctgtcacttcatcgaaaggacagtagaaaaggaag
gtggctcctacaaatgccatcattgcgataaaggaaaggctatcattcaagatctctctgccgacagtggtcccaaagatggacccc
cacccacgaggagcatcgtggaaaaagaagaggttccaaccacgtctacaaagcaagtggattgatgtgacatctccactgacgt
aagggatgacgcacaatcccactatccttcgcaagacccttcctctatataaggaagttcatttcatttggagaggacacgctactga
cctaataacttcgtatagcatacattatacgaagttatattaagggttgaatgaaaactaatctttttctctttctcatcttttcacttctc
ctatcattatcctcggccgaattcggaggtgtgagcaagggagaggaactgttcggcggcatcgtgcccatcctggtggagctggag
ggcgacgtgaacggccacaagttcagcgtgagcggcgagggcgagggcgacgccacctacggcaagctgaccctgaagttcatct
gcaccaccggcaagctgcccgtgccctggcccaccctggtgaccaccctgacctggggcgtgcagtgcttcagccggtaccccgac
cacatgaagcagcacgacttcttcaagagcgtgatgcccgagggctacgtgcaggagcggaccatcttcttcaaggacgacggca
actacaagacccgggccgaggtgaagttcgagggcgacaccctggtgaaccggatcgagctgaagggcatcgacttcaaggagg
acggcaacatcctgggccacaagctggagtacaactacatcagccataacgtgtacatcaccgccgacaagcagaagaacggcat
caaggccaacttcaaggcccggcacaacatcaccgacggcagcgtgcagctggccgaccactaccagcagaacacccccatcggc
gacggccccgtgatcctgcccgacaaccactacctgagcacccagagcgccctgagcaaggaccccaacgagaagcgggaccat
atggtgctgctggagttcgtgaccgccgccggcatcacccacggcatggacgaactgtacaaaggtggccatgatgagctttaagct
tctctagctagagtcgatcgacaagctcgagtttctccataataatgtgtgagtagttcccagataagggaattagggttcctatagg

gtttcgctcatgtgttgagcatataagaaacccttagtatgtatttgtatttgtaaaatacttctatcaataaaatttctaattcctaaaa

ccaaaatccagtactaaaatccagatcgctgacctaataacttcgtatagcatacattatacgaagttatattaagggttgaatgaa

aactaatctttttctctttctcatcttttcacttctcctatcattatcctcggccgaattcggaggtatggtgagcaagggcgaggagct

gttcaccggggtggtgcccatcctggtcgagctggacggcgacgtaaacggccacaagttcagcgtgtccggcgagggcgagggc

gatgccacctacggcaagctgaccctgaagttcatctgcaccaccggcaagctgcccgtgccctggcccaccctcgtgaccaccctg

acctacggcgtgcagtgcttcagccgctaccccgaccacatgaagcagcacgacttcttcaagtccgccatgcccgaaggctacgtc

caggagcgcaccatcttcttcaaggacgacggcaactacaagacccgcgccgaggtgaagttcgagggcgacaccctggtgaacc

gcatcgagctgaagggcatcgacttcaaggaggacggcaacatcctggggcacaagctggagtacaactacaacagccacaacg

tctatatcatggccgacaagcagaagaacggcatcaaggtgaacttcaagatccgccacaacatcgaggacggcagcgtgcagct

cgccgaccactaccagcagaacacccccatcggcgacggccccgtgctgctgcccgacaaccactacctgagcacccagtccgccc

tgagcaaagaccccaacgagaagcgcgatcacatggtcctgctggagttcgtgaccgccgccgggatcactctcggcatggacga

gctgtacaagggtggccatgatgagctttaagcttgctctcaagatcaaaggcttaaaaagctggggttttatgaatgggatcaaag

tttcttttttttcttttatatttgcttctccatttgtttgtttcatttccctttttgttttcgtttctatgatgcacttgtgtgtgacaaactctctg

ggtttttacttacgtctgcgtttcaaaaaaaaaaaccgctttcgttttgcgttttagtcccattgttttgtagctctgagtgatcgaattg

atgcctctttattccttttgttccctataatttctttcaaaactcagaagaaaaaccttgaaactctttgcaatgttaatataagtattgt

ataagatttttattgatttggttattagtcttacttttgctacctccatcttcacttggaactgatattctgaatagttaaagcgttacatg

tgttccattcacaaatgaacttaaactagcacaaagtcagatattttaagatcgcaccatttcgct 3’.

To amplify the *RPL* genomic sequence described in Experimental Procedures, the primers ATCTGGATCCGTATCGATAAGCGGATCCTTATTT and AAGGTCTAGATCTTTGGACCTACAAAATCATGTAGAAACTG were used.

To amplify sGFP(S65T) for in-frame fusion with *RPL* at the BamHI site, the primers used were: ATCTGGATCCATGGTGAGCAAGGGCGAGGA and ATCTGGATCCTTACTTGTACAGCTCGTCCA.

To amplify genomic *LSH4* fragments for fusion with sGFP(S65T), the following primer pairs were used: GTGGTCTCAGGAGTTTTACCATGCCTCCTTGCTC and GTGGTCTCTCACCATTAGGGCTACTTGAAATCGC; GTGGTCTCAGCTTCATTAAGTCATGAGTAAGATGT and GTGGTCTCAAGCGTCACACGCTCTCACCGTCTC.

To amplify sGFP(S65T) for in-frame fusion with *LSH4* at the *Bsa*I site, the primers used were: GTGGTCTCAGGTGGCATGGTGAGCAAG and GTGGTCTCAAAGCTTACTTGTACAGCT.

Chromatin Immunoprecipitation (ChIP)

pRPL:RPL-GFP rpl-1 and wild-type L-*er* control plants were used. For each replicate, 1.3-1.5 g of inflorescence apices were fixed under vacuum for 20 min in 35 ml of ice-cold fixation buffer (0.4 M sucrose, 10mM Tris pH 8, 1 mM EDTA pH 8.5, 1% formaldehyde, 100 µM PMSF). 100 µM glycine was added for 10 min on ice to stop cross-linking, followed by two washes with water. The tissue was blotted dry and frozen in liquid nitrogen, Nuclei were purified (49) and re-suspended in 1 ml of sonication buffer (500 mM Hepes, 150 mM NaCl, 5 mM MgCl2, 10% TRITON X-100) with half a tablet of protease inhibitor cocktail complete Mini, EDTA-free (Roche). After sonication in a Bioruptor sonicator at 4°C (2 x 5 min high power level with 30 sec on/30sec off cycles), producing an average fragment size of 500 bp, the samples were centrifuged, the supernatant was mixed with 500 ul of immunoprecipitation buffer (0.5M Hepes, 150 mM NaCl, 5 mM MgCl2 , 10% TRITON X-100, 1 mg/ml BSA) and 25 µl of anti-GFP µMACS Microbeads (Milteyi Biotec). After 30 min on ice, the samples were loaded on a µ Column (Milteyi Biotec) equilibrated with immunoprecipitation buffer, then placed into a magnetic µMACS separator (Milteyi Biotec). After washing twice with 400 µl and twice with 200 µl of immunoprecipitation buffer, twice

with 200 µl TE (100 mM Tris pH 8, 10 mM EDTA pH 8), the DNA was eluted once with 20 µl and twice with 50 µl of preheated (96° C) elution buffer (50 mM Tris pH8, 10 mM EDTA, 50 mM DTT and 1% SDS). 100 µl of TE-buffer and 9 µl of 25 mg/ml Proteinase K (Sigma) were added to the eluted samples and to the input control samples. Crosslinking was reverted by incubation at 37°C overnight, addition of 9 µl of 25 mg/ml Proteinase K and 8 h incubation at 65° C. After phenol-chloroform extraction, precipitation with ethanol overnight at -20° C, and washing with 70% ethanol, the air-dried DNA was re-suspended in 100 µl PCR-grade water (Roche), purified using a PCR purification Kit (18104, Qiagen) and stored at -80° C.

Analysis of ChIP-seq peaks (using Supplemental Software 2)

The protocol below was used after calling ChIP-seq peaks with MACS2 as described in the Experimental Procedures to filter ChIP-seq peaks for reproducibility across replicates, to associate peaks to gene models, to analyze the distribution of peaks within associated genes and to select input sequences for MEME-ChIP.

1. Installation

To use the scripts, expand the folder *Peaks_analysis* (Supplemantal Software 2) and place it on the Desktop. The scripts were written in Python 2.7.3 on an Apple computer running MacOS X 10.9.4 - changes may be needed to install and run them on a different platform. Dependencies are Numerical Python (http://www.numpy.org), matplotlib (http://matplotlib.org) and Tcl/tk (https://www.python.org/download/mac/tcltk/).

2. Filtering peaks for reproducibility across replicates and attributing gene models

ChIP-seq reads from three replicate treatments and three replicate controls were aligned against the genome and peaks called as described in Experimental Procedures). To calculate fold enrichments and *q*-values, the combined replicates were compared with the combined controls using MACS 2.0.10 (Feng et al., 2012); this generated the files RPL-GFP_fused_peaks.narrowPeak and wt_fused_peaks.narrowPeak.gz. In addition, MACS 2.0.10 was applied to individual replicates to select for consistency across replicates; this generated files RPL-GFP1_peaks.narrowPeak, RPL-GFP2_peaks.narrowPeak, RPL-GFP3_peaks.narrowPeak, wt1_peaks.narrowPeak, wt2_peaks.narrowPeak, wt3_peaks.narrowPeak. These files are deposited at NCBI (http://www.ncbi.nlm.nih.gov/geo/query/acc.cgi?&acc=GSE78727) and need to be placed together in a local folder for the analysis below.

To filter for consistency across replicates, the script Overlap_MACS2_files.py is called by double-clicking on the shell script ~/Peaks_analysis/shell_scripts/Overlap_MACS2_files.sh. The script searches for overlaps between peak regions listed in selected replicates (GFP1_peaks.narrowPeak, RPL-GFP2_peaks.narrowPeak, RPL-GFP3_peaks.narrowPeak files), and accepts the overlaps if they are absent from all controls, have q-values of a specified value or lower in each replicate, and the overlapping region is at least of a specified length.

After the filtering step above, peaks are attributed to gene models and an annotated table is produced. For this, tables with gene coordinates and with gene annotations are selected - see ~/Peaks_analysis/TAIR10_tables/TAIR10_AGI_location.txt and TAIR10_functional_descriptions.txt. Based on these tables, peaks are associated

with gene models within specified distances upstream and downstream of the transcribed regions, without intervening genes.

Inputs are selected interactively:

path to folder containing narrowPeak files

path to table containing gene positions

path to table containing gene annotations

Parameters are set directly on the script (using a suitable editor such a IDLE):

n is the minimum width in nucleotides for accepting the overlap between MACS peaks (default n = 50)

prom is the length in nucleotides for the upstream regulatory region for each gene model (used to associate peaks with genes); default prom = 4000

utr is the length in nucleotides for the downstream regulatory region for each gene model (used to associate peaks with genes) default utr = 1500

chromosomes specifies chromosome names and lengths in nucleotides

default chromosomes = np.array([['Chr1', 30500000], ['Chr2', 19700000], ['Chr3', 23500000], ['Chr4', 18600000], ['Chr5', 27000000]])

fc_cutoff sets the position of bedgraph line for fold change; default fc_cutoff = 3

q_cutoff = 3 sets cutoff q value for overlapping peaks from different replicates; default q_cutoff = 3

Outputs are:

A tab-delimited text table with the MACS2 statistics, gene models and annotation associated with each overlapping peak region (named Overlapping_peaks_AGI.txt, saved in the same folder as the narrowPeak files).

A tab-delimited text table with the nucleotide position of the center of each overlapping peak region (named "Peak_positions.txt" and saved in the same folder as the narrowPeak files).

bedgraph files (which can be opened in the IGV browser) for peak fold changes (Overlapping_peaks_fold_change.bedgraph) and for peak q-values (Overlapping_peaks_q_values.bedgraph), both placed in the same directory as the narrowPeak files.

3. Analyzing the distribution of peaks within associated genes

To statistically analyze peak locations within genes, the script peak_statistics.py is called by double-clicking on the shell script ~/Peaks_analysis/shell_scripts/Peak_statistics.sh. This script interactively selects the file with the position of overlapping peak regions and the file with associated gene models (both produced in step 2 above), the table with gene coordinates (~/Peaks_analysis/TAIR10_tables/TAIR10_AGI_location.txt), and asks for the number of replicates used when scoring the positions of simulated, random peaks.

The script scores the frequency of observed peak regions centered on the transcribed, upstream and downstream regions, then uses a Monte Carlo method to estimate the *p*-value for the hypothesis that these frequencies correspond to a random distribution of peaks within genes. A tab-delimited text table with the results is produced, in addition to histograms showing the frequency of observed and simulated (random) peaks at different distances to the start and end of transcribed regions (Peak_position_statistics.txt, Peaks_histogram_upstream.png,

Peaks_histogram_downstream.png, all saved in the same folder as the selected file with peak positions).

Set parameters chromosomes, which specify chromosome names and lengths in nucleotides; default values are:

chromosomes = np.array([['Chr1', 30500000], ['Chr2', 19700000], ['Chr3', 23500000], ['Chr4', 18600000], ['Chr5', 27000000]])

4. Selecting input sequences for MEME-ChIP

To detect enrichment for sequence motifs within overlapping peak regions, MEME-ChIP (http://meme-suite.org/tools/meme-chip) was used in discriminative mode, comparing the sequences around observed peaks with a control set of sequences around a ten-fold larger number of random peaks (Experimental Procedures). To produce both sets of sequences, the script peak_sequences.py is called by double-clicking on the shell script ~/Peaks_analysis/shell_scripts/Peak_sequences.sh.

The script interactively selects the file with the positions of overlapping peak regions and the file with associated gene models (both produced in step 2 above), the table with gene coordinates (~/Peaks_analysis/TAIR10_tables/TAIR10_AGI_location.txt), then asks for the size of the region to include on each side of the center of each peak region. A folder is also selected with chromosome sequences. This is not included here and must be downloaded from public databases (e.g. TAIR, http://www.arabidopsis.org) - one file for each chromosome, with a single sequence in FASTA format; the files must be named "TAIR10_chr1.fas", "TAIR10_chr2.fas" etc.

To produce a control set of sequences for discriminative MEME, the script generates random peak positions in each chromosome. The size of the control set (how many times larger than the observed set) is set interactively.

The outputs are a list of sequences flanking the centers of observed peak regions (Peak_sequences.txt) and a list of sequences flanking random peak centers (Random_sequences.txt), both in FASTA format, saved in the same folder containing the input file with the positions of peak regions.

Set parameters chromosomes, which specify chromosome names and lengths in nucleotides; default values are:

chromosomes = np.array([['Chr1', 30500000], ['Chr2', 19700000], ['Chr3', 23500000], ['Chr4', 18600000], ['Chr5', 27000000]])

Quantitative reverse transcription PCR

Quantitative reverse transcription-polymerase chain reaction (qRT-PCR) was performed with the LightCycler 480 System and SYBR Green I (Roche) and the primers listed below. Enrichment from ΔCt values (Ct immunoprecipitated DNA – Ct input DNA) using the 2-ΔΔCt method as described (Livak and Schmittgen, 2001). Data were normalized to *ACTIN2* amplified with the primers below. For each biological replicate, the average value of three technical replicates was used; two-tailed Student's t-tests were used to test for statistical significance of differences between sets of biological replicates.

The following primers were used: for *LSH4*, ACCAATTCGGCAAGACTAAGGTTC and AGCAGCTCTAAGACGGCCAATG; for *LOB*, TGCGTCGGAGCCATCTCTTATC and AGTCAGCATTAGCTGCGTCGAG; for *ACTIN 2*, GCACCCTGTTCTTCTTACCG and AACCCTCGTAGATTGGCACA.

Confocal imaging

mPS-PI (modified pseudo-Schiff-propidium iodide) imaging was modified from (Truernit et al., 2008). Inflorescence tips (terminal 0.5 cm) were fixed for 15 min each in 15%, 30%, 50%, 70%, 85%, 95% and 100% ethanol. After 16h in ethanol, all floral buds older than stage 3 (Smyth et al., 1990), the samples were rehydrated through the same ethanol series, washed in water and incubated at 37°C overnight in alpha-amylase (Sigma) 0.3 mg/mL in phosphate buffer 20 mM pH7.0, 2 mM NaCl, 0.25 mM $CaCl_2$. The apices were then rinsed in water, treated 30 min in 1% periodic acid, washed twice in water and incubated 2 h in Schiff-PI reagent, cleared with chloral hydrate solution and mounted in Hoyer's medium (Truernit et al., 2008), before imaging with a Zeiss LSM780 confocal microscope with excitation at 488 nm, emission filters set to 572-625 nm, using a X40/1.0 dipping objective; image resolution was 0.42 X 0.42 X 0.5 µm.

Image analysis (using Supplemental Software 1)

The protocol below explains how to install and use a set of Python scripts and Fiji macros used to analyze the orientation of recent cell divisions in plant tissues and for the analysis of marked clones in three dimensions.

These scripts are based on a previously published set of scripts for 3D segmentation, cell measurements and cell tracking (Serrano-Mislata et al., 2015). Only new scripts are described below; for details of published scripts, please see the corresponding instructions (Serrano-Mislata et al., 2015).

Fiji (Schindelin et al., 2012) is used to visualize and interact with processed images (e.g. to select landmarks on the image) and Fiji macros are used to facilitate this. It is assumed that the user is familiar with Fiji in general and in particular with the plugins 3D

Viewer (Schmid et al., 2010) and Pointpicker. For instructions on how to use these, please refer to:

http://fiji.sc/Getting_started

http://fiji.sc/3D_Viewer

http://bigwww.epfl.ch/thevenaz/pointpicker/

1. Installation

To use the scripts, expand the folder *Rib_zone_analysis* (Supplemental Software 1) and place it on the Desktop (other locations will work, but will require editing the paths mentioned below). To read and write images in the correct location, the path to images must be edited in the Fiji macro *~/Rib_meristem_analysis/Fiji_macros/confocal_to_TIF.ijm*; open the script with Fiji and edit the path attributed to the variable "ANALYSIS_PATH" in the first line; use the path leading to the Rib_meristem_analysis folder (e.g. in my case "/Users/Author/Desktop/Rib_meristem_analysis/")

The scripts require Numerical Python (http://www.numpy.org), Scientific Python (http://www.scipy.org), matplotlib (http://matplotlib.org) and SimpleITK (http://www.simpleitk.org). To install these dependencies using MacOS X 10, open a Terminal session and type after the "$" sign (you will need an administrator password):

sudo easy_install numpy

sudo easy_install scipy

sudo easy_install matplotlib

sudo easy install SimpleITK

To check that the required dependencies are in place, open a Python session in Terminal (type “python”) and try to import the packages by typing the lines below after the “>>>” prompt; if no error message appears, you will be ready to run the image analysis scripts.

```
import numpy
import scipy
import matplotlib
import SimpleITK
```

For the Fiji macros, install the latest version of Fiji (http://fiji.sc/Fiji). Make sure that the plugin 3D Viewer is listed in the plugins menu; if not, download and install (http://fiji.sc/3D_Viewer). The plugin Pointpicker is also required; download (http://bigwww.epfl.ch/thevenaz/pointpicker/) and install. Specific lookup tables (LUT) are also required to visualize the images: go to Applications, open the Fiji folder (it may be necessary to use ctrl click, “Show package contents”), then copy into the folder “LUT” the following files:

~ */Rib_meristem_analysis/LUTs/seg.lut*

~ */Rib_meristem_analysis/LUTs/walls_hm.lut*

After copying the LUTs, re-start Fiji and check that the LUTs appear in the pull-down menu *Image>Lookup Tables.*

The scripts and macros were written in Python 2.7.3 on an Apple computer running MacOS X 10.9.4 and Java 6 - changes may be needed to install and run them on a different platform.

2. Setting up the images table

The scripts are managed through a table that specifies where the input and output images are located, and what scripts are needed for the analysis. This allows the analysis to be customized. Another advantage of using the images table is that it serves as a database of all images analyzed.

To set up the images table, use *~/Rib_meristem_analysis/processed_images/images_table.csv* as the template (~ indicates the path leading to the *Rib_meristem_analysis* folder in the computer where the scripts are installed). The scripts will look for this table path and name, so both must remain unchanged.

The table provided already contains lines to process test images. These are meant just as examples of how to fill in the table and will not work with the scripts because the corresponding image folders are absent from *~/Rib_meristem_analysis/processed_images/*. If you would like to create the required folders to test run the scripts, the test images can be found in https://open-omero.nbi.ac.uk (username "shared", password "Op3n-4cc0unt" - note that all non-image files, such as Metadata, data tables, list of landmark coordinates, will be found as attachments of the image ending with "_seg.tif")

You can use e.g. Excel or TextEdit to add new lines to the table for new images to be analyzed, but make sure that the table is saved as comma-separated values (.csv).

The following fields are filled in for each new image stack:

- Your name (optional)

- Notebook number and page for experiment (optional)

- Date (optional)

- Path to Rib_meristem_analysis folder (required - in the example lines, this is set to /Users/Author/Desktop/ Rib_meristem_analysis/processed_images/; please change this to the actual path leading to the files in the computer where the scripts will be used)

- Folder name (required - this is a unique identifier for the images, which is called rootname in the rest of this protocol)

Additional columns track the progress through the scripts mentioned below, which will be called sequentially by clicking on the shell script *~/Rib_meristem_analysis/shell scripts/Rib_meristem_analysis*. Whenever marked “0”, the image has not yet been processed by that script; after processed, the column is automatically marked “1”. If a script needs to be run again on an image, just change the value for that script back to “0” before running the batch script again. If a script is not relevant to the analysis of a particular image, the corresponding position on the table should be marked with any other character, e.g. “x”.

3. Selecting and cropping confocal images

The image analysis starts with separate stacks for each confocal channel and a metadata file with information such as voxel sizes. To create the stacks, use the Fiji macro *~/Rib_meristem_analysis/Fiji_macros/confocal_to_TIF.ijm* (select using

Fiji>Plugins>Macros>Run). Follow the instructions to open the confocal image, specify the rootname for the images, crop the image and split the channels. The macro creates a folder within *~/Rib_meristem_analysis/processed_*images, named with the given rootname and containing TIF stacks for each of the channels selected: rootname_R.tif for FM4-64/PI channel, rootname_G.tif for GFP channel, if used. In addition, a Metadata.txt file is created, with information about the file name, path to original confocal stack and voxel sizes. The Metadata file will also be updated with the specific parameters used by each subsequent script used to analyse the images. The folders with test images are provided with the output of c *confocal_to_TIF.*ijm, i.e. Metadata, _R.tif and _G.tif files.

4. Landmarking

Landmarks need to be added as reference points to select different regions (such as meristem and buds), to find the main axis of the apex etc. These landmark files need to be given specific names to be called by the Python scripts. For this, run the Fiji macro landmarks_3D.ijm (select using Fiji>Plugins>Macros>Run). Follow the instructions on the screen; typically the image to open is rootname_R.tif.

When processing images to measure cell wall orientations (see below), the main axis (e.g. of the stem) must be defined. For this, select 2 points must be selected in the order: top (e.g. inflorescence meristem summit), and bottom (e.g. center of stem near the bottom of the image). Save landmarks as rootname_axis.

When processing images to analyze Cre-loxP sectors, landmarks are placed at the boundaries of floral buds to find the main axis and to subsequently align and superimpose multiple images. For this, select a point at the center of the boundary

between the meristem and a floral bud, for the 5 youngest buds. Save landmarks as rootname_boundaries.

For the sectors analysis, it is also necessary to manually landmark the cells in each sector using the Fiji macro *mark_GFP_sectors.ijm* (Fiji>Plugins>Macros>Run). Follow the instructions on the terminal to open the image of cell outlines (rootname_R.tif) and the image with GFP signal (rootname_sectors_G.tif). Adjust its brightness of the GFP image on the B&C window, click on "Apply", say "Yes" to applying to the whole stack, then click on the "OK" button in the window "Please adjust brightness and contrast". You will see a Point Picker window with the images fused and a yellow square on the top left corner. Scroll up and down the image, choose a sector and click only once in each cell of the sector; you will see crosses appearing on the marked cells. Before moving to the next sector, click on the square in the corner - this is important to create a marker point to separate each sector. Save points as rootname_sectors (use the button with a page icon on the Fiji menu bar), then click on "Click OK when done".

5. Segmentation and cell measurements

Once the rootname_R.tif, rootname_G.tif (if applicable), Metadata.txt and landmarks (.points) files are in place, the images can be segmented and the cells measured. For segmentation, measurement of cell volumes and position relative to the main axis and to the apex, the scripts *watershed_segmentation.py, cell_data_table.py* and *rib_zone.py* are called. Details of these scripts, input parameters and output are given in the instructions for 3D_meristem_analysis (Serrano-Mislata et al., 2015). To run these scripts and the new cell wall scripts

below, set the corresponding columns in the images table to "0" and double click on the shell script *~/Rib_meristem_analysis/shell scripts/Rib_meristem_analysis.*

To visualize segmented images, open them in Fiji and select the LUT “seg” on the pull down menu *Images>Lookup tables*. If the colors are not displayed correctly, it is necessary to re-set the LUT; for this, run the Fiji macro reset_LUT (select using Fiji>Plugins>Macros>Run).

6. Finding new walls and their orientation

After running *watershed_segmentation.py, cell_data_table.py* and *rib_zone.py*, it is possible to identify newly deposited cell walls and their orientation by calling the scripts:

new_walls.py

This script detects newly deposited cell walls in images of tissue stained by mPS-PI (Truernit and Haseloff, 2008), assuming that mPS-PI signal is proportional to wall thickness. A new wall is flagged when two neighboring cells share the same wall as their wall with lowest intensity. To correct for diminishing intensity with increasing depth in the confocal stack, the mPS-PI image in each plane is normalized using the average signal within the segmented cells. The normalized cell wall intensities are then corrected for the bias introduced by the fact that walls parallel to the imaging plane appear weaker; for this, a corrective function is obtained by plotting all wall intensities as a function of their angle to the imaging plane. The corrected cell wall intensities are added to the walls data table and an image of new walls is saved.

Inputs are the mPS-PI confocal stack (_R.tif) and the corresponding segmented image (_seg.tif). If available, an image of cell walls and the corresponding walls data table are read, otherwise they are created.

Default parameters are: cs = 20 (size of the image cropped around each cell wall during processing); wmin = 165 (minimum number of voxels in accepted cell walls); wmax = 2000 (maximum number of voxels in accepted cell walls). If needed, these parameters can be changed directly on the script with a standard script editor.

Outputs are the walls data csv table containing corrected cell wall intensities and with new walls marked, and a 16 bit TIF image stack of the new walls.

cell_wall_orientation.py

This script takes an image of newly deposited cell walls (produced by the script "new_walls.py") and calculates the orientation of each new wall in relation to the image main axis. "Angle to the main axis" is the angle between the given vector for the image main axis and the vector normal to plane best fitting the wall; "Angle to the radial axis" is the angle between the vector normal to best fitting plane and a vector perpendicular to the main axis that crosses the wall's center of mass.

Input files are the segmented image, corresponding image of cell walls, image or landmarks for the main axis, cell data table and cell walls table. The calculated angles are added to the walls data table (produced by the script "new_walls.py") and the cell data tables. Images are produced in which the new walls are given values proportional to the angles to the main axis or radial axis (heat map images"_new_wall_angles_main_axis.tif" and"_new_wall_angles_radial_axis.tif").

Images are also saved in which the two cells flanking each new wall are given values proportional to the angles to the main axis or radial axis (heat map images"_new_cell_angles_main_axis.tif" and"_new_cell_angles_radial_axis.tif"). Default parameters are: cs = 20 (size of the image cropped around each cell wall during processing) bar_min = 0 (minimum values for angle in heat map images) bar_max = 90 (maximum values for angle in heat map images). If necessary, these parameters can be changed directly on the script with a standard script editor.

To visualize the heat map images, open in Fiji and choose the LUT "walls_hm" on the pull-down menu *Image>Lookup Tables.* To visualize the image of segmented cell walls, open in Fiji and select the LUT "seg" on the pull down menu *Images>Lookup tables*; if the colors are not displayed correctly, run the Fiji macro reset_LUT (select in Fiji>Plugins>Macros>Run).

7. Analysis of Cre-loxP sectors

To detect Cre-loxP sectors and their 3D orientation, it is necessary first to landmark the organ boundaries, manually mark the cells in each sector, segment and measure the cells (see steps 4 and 5 above). After this is done, make sure that the columns for the scripts below are set to "0". Select only the images that you want to merge if different genotypes are compared, each group of images has to be overlapped and analyzed in a different run. After the images table is set up and saved, run the shell script *~/Rib_meristem_analysis/shell scripts/Clonal_analysis.* This will call the following scripts:

cell_layers.py

This script attributes cells to tissue layers. This is necessary because the centers of mass of epidermal cells are used to produce an outline of the superimposed apices with marked sectors. This script is described in detail in the instructions for 3D_meristem_analysis (Serrano-Mislata et al., 2015).

sectors_merge.py

This script will save in each rootname folder an image (rootname_sector_landmarks.tif), in which dots labeled with a unique number are placed in the coordinates of the landmarks found in the file rootname_sectors.txt. The centers of epidermal cells are also marked to produce an outline of the apex. A similar image is produced in which the same landmark dots are labelled with sector numbers. A table is produced (rootname_landmarks_data.csv), listing the landmarks, their sector numbers, coordinates, distance to the apex and main axis, and measurements of the corresponding cells listed in the table (rootname_cell_data.csv) produced by *cell_data_table.py*.

In addition, three new images and a table are placed in the path *~/Rib_meristem_analysis/processed_images/*. In the image Merged_landmarks.tif, each of the rootname_sector_landmarks.tif image was rotated to align the main axis of the stem with the central axis of the image (running vertically through the centre), shifted to place the meristem summit point on plane 10 and rotated around the main axis to align the bud landmarks found in the file rootname_boundaries.points (bud P0 is placed at position 12 o'clock and older buds are aligned clockwise).

Merged_landmarks.tif is a similar overlap of all the aligned

rootname_sector_landmarks.tif images. Merged_boundaries.tif is another overlap of aligned images, but showing the positions of the landmarks placed on the bud boundaries in each image (to check how well the alignment worked). The table Merged_sectors_data.csv lists all the landmarks in Merged_landmarks.tif, the rootname of the image from which they originated, their coordinates, and the corresponding data from each of the rootname_cell_data.csv tables.

Inputs are:

images_table.csv; segmented image (rootname_seg.tif); image of main axis of the stem (rootname_main_axis.tif, produced by script rib_zone.py); file with coordinates of landmarks placed on each cell in sectors (rootname_sectors.txt); file with coordinates of landmarks placed on bud boundaries (rootname_boundaries.points)

Default parameters are:

radius = 2 (size in micrometers of sphere used to label landmarks on image); cs = 20 (defines the size of the image cropped around each sector during processing); sm = 1000 (defines value to label summit point; has to be more than the total number of objects in the merged sectors image).

sectors_analyse.py

This script uses the coordinates of sector landmarks in the merged image produced by *sectors_merge.py* to calculate the main axis of each sector and its orientation. To calculate the vector of the main axis, the linalg.svd (single value decomposition) function of Numpy is used. To calculate the size of the main axis, a plane perpendicular to the vector is placed on the center of mass of the sector landmarks; the distance between each landmark and the plane is calculated, and the length of

the main axis on each side of the plane equals the maximum landmark distance for that side. The orientation of the sector main axes is calculated as angle to the main axis (central z on the merged images) and to a vector perpendicular to the main axis and crossing the center of mass of the sector landmarks.

Images are produced of the sector landmarks, labeled with the corresponding sector number (Sector_landmarks.tif), of the corresponding sector axes (Sector_axes.tif) and of the sector axes overlapped with sector landmarks, to verify that the axes have been calculated correctly (Sector_axes_and_landmarks.tif). A table is also produced (Sectors_analysis.csv) with the coordinates for the center of mass, main axis extremities, main axis orientation and size.

In addition, the script produces the images "Sectors_landmarks_radial.png", Sectors_landmarks_top.png ", "Sector_3D_axes_radial.png", "Sector_3D_axes_top.png". These correspond to top projections (along the z axis) and radial projections of the landmark and axis images. To make the radial projection, each sector/axis was projected onto a plane containing its center of mass and the main image axis; sectors/axes on the right and left sides of the vertical projection are placed respectively on the right and left sides of the radial projection.

Finally, a lut table is saved, listing the RGB colors attributed to each sector/axis on the projections. This lut table can be used imported by Fiji (Plugins/LUT_editing/LUT importer) to visualize the landmarks and axes in the images Sector_landmarks.tif, Sector_axes.tif and Sector_axes_and_landmarks.tif.

Inputs are:

table "Merged_sectors_data.csv" (produced by sectors_merge.py); image "Merged_sectors.tif" (produced by sectors_merge.py); text file "Merged_images_metadata.txt" (produced by sectors_merge.py)

Default parameters are:

sm = 1000 (value attributed to summit point when aligning images; must match the value used in sectors_merge.py); radius = 2 (size in micrometers of sphere used to label landmarks on images); cs = 20 (defines the size of the image cropped around each sector during processing)

After running these scripts for one set of images, remember to save the files with a different name or move them to a different folder, or they will be overwritten by the subsequent run.

Supplemental References

Feng, J., Liu, T., Qin, B., Zhang, Y. & Liu, X. S. 2012. Identifying ChIP-seq enrichment using MACS. *Nature Protocols,* 7**,** 1728-1740.

Livak, K. J. & Schmittgen, T. D. 2001. Analysis of relative gene expression data using real-time quantitative PCR and the 2(T)(-Delta Delta C) method. *Methods,* 25**,** 402-408.

Schindelin, J., Arganda-Carreras, I., Frise, E., Kaynig, V., Longair, M., Pietzsch, T., Preibisch, S., Rueden, C., Saalfeld, S. & Schmid, B. 2012. Fiji: an open-source platform for biological-image analysis. *Nat. Methods,* 9**,** 676-682.

Schmid, B., Schindelin, J., Cardona, A., Longair, M. & Heisenberg, M. 2010. A high-level 3D visualization API for Java and ImageJ. *BMC Bioinformatics,* 11**,** 274.

Serrano-Mislata, A., Schiessl, K. & Sablowski, R. 2015. Active control of cell size generates spatial detail during plant organogenesis. *Curr. Biol.,* 25**,** 2991-2996.

Smyth, D. R., Bowman, J. L. & Meyerowitz, E. M. 1990. Early flower development in Arabidopsis. *Plant Cell,* 2**,** 755-67.

Truernit, E., Bauby, H., Dubreucq, B., Grandjean, O., Runions, J., Barthelemy, J. & Palauqui, J.-C. 2008. High-resolution whole-mount imaging of three-dimensional tissue organization and gene expression enables the study of phloem development and structure in Arabidopsis. *Plant Cell,* 20**,** 1494-1503.

Truernit, E. & Haseloff, J. 2008. A simple way to identify non-viable cells within living plant tissue using confocal microscopy. *Plant Methods,* 4**,** 15.
